# Internet-Based Assessment of Oncology Health Care Professional Learning Style and Optimization of Materials for Web-Based Learning: Controlled Trial With Concealed Allocation

**DOI:** 10.2196/jmir.7506

**Published:** 2017-07-25

**Authors:** Christine M Micheel, Ingrid A Anderson, Patricia Lee, Sheau-Chiann Chen, Katy Justiss, Nunzia B Giuse, Fei Ye, Sheila V Kusnoor, Mia A Levy

**Affiliations:** ^1^ Division of Hematology and Oncology Department of Medicine Vanderbilt University Medical Center Nashville, TN United States; ^2^ Vanderbilt-Ingram Cancer Center Vanderbilt University Medical Center Nashville, TN United States; ^3^ Center for Knowledge Management Vanderbilt University Medical Center Nashville, TN United States; ^4^ Department of Cancer Biology Vanderbilt University Nashville, TN United States; ^5^ Department of Biomedical Informatics Vanderbilt University Medical Center Nashville, TN United States; ^6^ Department of Biostatistics Vanderbilt University Medical Center Nashville, TN United States

**Keywords:** e-learning, Web-based Instruction, learning, teaching materials, information dissemination, online systems, education, distance, continuing education, medical oncology/education

## Abstract

**Background:**

Precision medicine has resulted in increasing complexity in the treatment of cancer. Web-based educational materials can help address the needs of oncology health care professionals seeking to understand up-to-date treatment strategies.

**Objective:**

This study aimed to assess learning styles of oncology health care professionals and to determine whether learning style-tailored educational materials lead to enhanced learning.

**Methods:**

In all, 21,465 oncology health care professionals were invited by email to participate in the fully automated, parallel group study. Enrollment and follow-up occurred between July 13 and September 7, 2015. Self-enrolled participants took a learning style survey and were assigned to the intervention or control arm using concealed alternating allocation. Participants in the intervention group viewed educational materials consistent with their preferences for learning (reading, listening, and/or watching); participants in the control group viewed educational materials typical of the My Cancer Genome website. Educational materials covered the topic of treatment of metastatic estrogen receptor-positive (ER+) breast cancer using cyclin-dependent kinases 4/6 (CDK4/6) inhibitors. Participant knowledge was assessed immediately before (pretest), immediately after (posttest), and 2 weeks after (follow-up test) review of the educational materials. Study statisticians were blinded to group assignment.

**Results:**

A total of 751 participants enrolled in the study. Of these, 367 (48.9%) were allocated to the intervention arm and 384 (51.1%) were allocated to the control arm. Of those allocated to the intervention arm, 256 (69.8%) completed all assessments. Of those allocated to the control arm, 296 (77.1%) completed all assessments. An additional 12 participants were deemed ineligible and one withdrew. Of the 552 participants, 438 (79.3%) self-identified as multimodal learners. The intervention arm showed greater improvement in posttest score compared to the control group (0.4 points or 4.0% more improvement on average; *P*=.004) and a higher follow-up test score than the control group (0.3 points or 3.3% more improvement on average; *P*=.02).

**Conclusions:**

Although the study demonstrated more learning with learning style-tailored educational materials, the magnitude of increased learning and the largely multimodal learning styles preferred by the study participants lead us to conclude that future content-creation efforts should focus on multimodal educational materials rather than learning style-tailored content.

## Introduction

Precision medicine is the use of a patient’s molecular characteristics to determine disease risk, make a precise diagnosis, determine disease prognosis, and to select the best treatment plan for the patient. In the field of cancer, researchers have been working to develop new drugs and therapeutic strategies tailored to cancers harboring particular biomarkers. Breast cancer has a long history of biomarker-driven prediction of sensitivity to targeted therapies. This study used educational materials on inhibition of cyclin-dependent kinases 4/6 (CDK4/6) to block cell growth as a therapeutic strategy being investigated in patients with hormone receptor-positive breast cancer, including those who have developed resistance to endocrine therapy. These materials were used to investigate oncology health care professional learning styles and optimization of materials for Web-based learning.

Rapidly evolving information about precision cancer medicine creates a knowledge gap in the education of oncology health care professionals regarding complex and important precision cancer medicine concepts, along with approaches for identifying therapeutic strategies for individual patients [1-4]. For example, a large survey in 2011 showed that oncology nurses did not discuss mutation testing with patients because they felt they lacked the knowledge to do so [1]. More recently, an international survey demonstrated that a majority of lung cancer oncologists understand that improved survival is associated with therapies selected after epidermal growth factor receptor (*EGFR*) mutation testing, but a quarter of lung cancer oncologists do not consider the specific *EGFR* mutation detected in making therapeutic decisions [4]. This knowledge gap needs to be addressed quickly and effectively to bring the promise of precision cancer medicine to all cancer patients. The topic of CDK4/6 inhibitors in breast cancer was chosen because it was a timely, representative topic in the field of precision cancer medicine with an active knowledge gap.

Web-based tools provide an important platform for oncology health care professionals to address this knowledge gap. Websites such as UpToDate [5] are heavily used by physicians of all types. A wealth of clinical trial information can be found at ClinicalTrials.gov [6] or on the National Cancer Institute’s website [7]. We have developed the My Cancer Genome website [8] as a publicly accessible knowledge resource targeted at oncology health care providers. My Cancer Genome provides up-to-date information to oncology health care providers on the clinical relevance of mutations in cancers and gene-specific clinical trials. Launched in 2011, My Cancer Genome receives more than 10,000 site visits per week, from 211 countries and territories across the world, from an audience of health care providers, researchers, and patients/caregivers (usage statistics current as of January 2017). My Cancer Genome provides content pages on 23 cancer types, 823 genes, and 456 disease gene-variant relationships (content statistics current as of May 2017). Breast cancer educational content includes information on the therapeutic implications of alterations in several genes, including links to relevant clinical trials. A companion mobile app has also been available for Apple iOS devices since 2013.

An individual’s learning style refers to how that individual prefers to gather, interpret, organize, and think about information [9]. For example, individuals may vary in their preferences for how they receive educational information. Examples include preferences for learning through visual (eg, pictures, graphs, diagrams, charts), auditory, and text-based (eg, lists, bullets, or hierarchically organized text) formats [10]. Several learning style assessments have been developed that evaluate preferences according to different learning style models [11]. A person’s learning style preferences may change throughout their lifetime, and cultural factors and previous experiences may contribute to differences in preferences [11-14].

The literature on learning styles in the medical setting has predominantly focused on medical students and residents [13-18]. Learning style preferences may vary based on level of training [19,20]. For example, one small study reported differences in the prevalence of multimodal learners among Australian rural general practice registrars compared to rural medical students [19].

Tailoring education based on learning style may facilitate comprehension of the information. In a study evaluating the effect of providing medical students with instruction matched according to learning styles or in a standard format, tailored instruction was found to result in enhanced understanding of the material based on improvement in test scores, with statistically significant differences seen for kinesthetic learners [13]. Similarly, prior research has demonstrated that providing patients with health educational information customized to their health literacy and learning style preferences increased understanding and retention of the material [21-24]. However, in an updated systematic review and meta-analysis, Cook [25] found that evidence was lacking to support the use of adaptation to cognitive and learning styles in computer-assisted instruction. More rigorous studies are needed to better understand the effectiveness of tailored instruction on Web-based learning. To the best of our knowledge, no studies have been published reporting the learning style preferences of oncology health care professionals. Considering health care providers’ learning style preferences when developing educational content for Web-based tools may help accelerate understanding and retention of the information.

### Objectives

In this study, we developed educational information tailored to different learning styles on the topic of treatment of metastatic estrogen receptor-positive (ER+) breast cancer using CDK4/6 inhibitors. The objectives of this study were to (1) assess learning style preferences of oncology health care professionals and (2) evaluate the effectiveness of providing educational materials customized to learning style preferences using a fully automated, controlled study design with concealed allocation.

## Methods

The CONSORT-EHEALTH checklist for this study can be found in Multimedia Appendix 1. Technical details and changes after study commencement can be found in Multimedia Appendix 2.

### Ethics

This study was approved by the Vanderbilt University Medical Center institutional review board (IRB). This study received a waiver of consent from the IRB. In place of the traditional consent process, when participants clicked the link in their invitation email, they arrived at the welcome page. This page provided participants with information about the study’s purpose, mechanics, and risks, with contact information for those with further questions. The welcome page also provided an estimate of the time commitment needed to participate in the study. Participants then entered their email address, personal identification number (PIN) as provided in their invitation email, and answered yes or no to the statement, “I agree to participate in this study.” We did not receive any phone calls or emails from participants prior to their agreement to participate.

### Study Design

**Figure 1:**
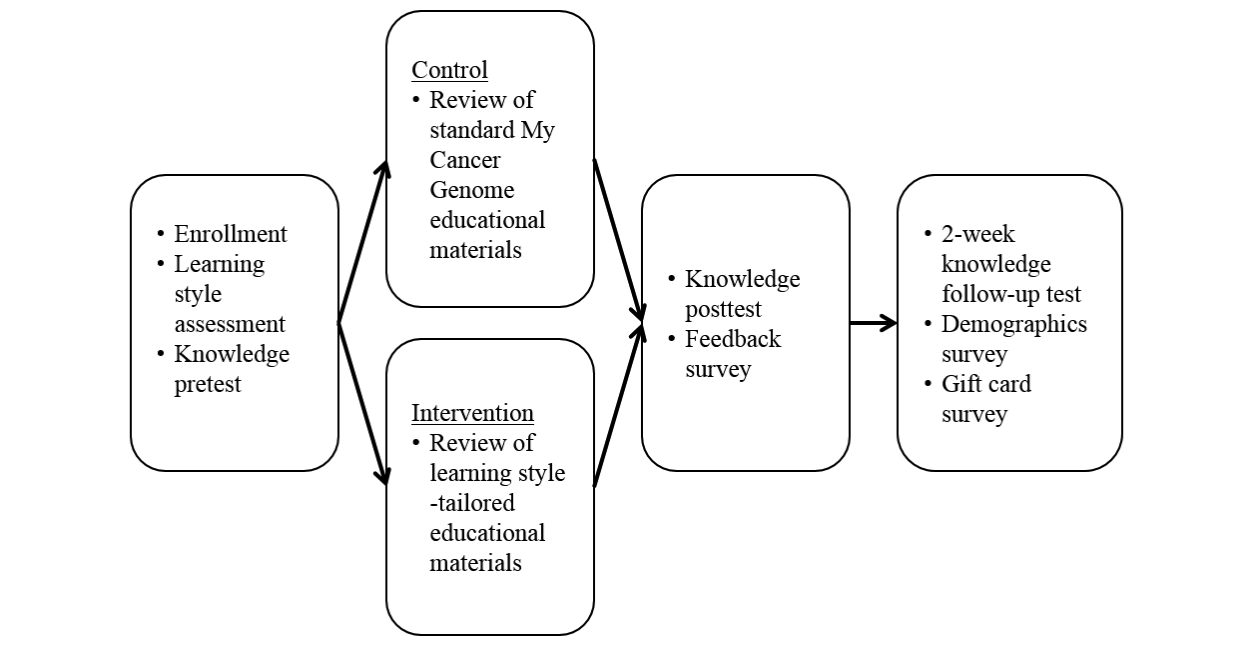
Study design.

A parallel study design was used to examine the effectiveness of providing learning style-tailored materials compared to control materials (Figure 1). The study opened on July 13, 2015, and closed on September 7, 2015. Participants were allocated 1:1 to the intervention or control arms when they landed on the enrollment screen. Participants were asked to complete a learning style assessment and a knowledge pretest. Participants allocated to the intervention arm viewed materials consistent with their learning style preferences, and participants allocated to the control arm viewed materials in the format used on My Cancer Genome. Following review of the educational materials, all participants were asked to complete a knowledge posttest and a feedback survey. After 2 weeks, participants who had completed the posttest were asked to take the knowledge follow-up test. The 2-week interval was chosen for consistency with the authors’ related research [21,24]. After taking the knowledge follow-up test, participants could provide demographic information and fill out a form to provide the information needed to send the US $100 Amazon.com Gift Card incentive.

### Data Security

Safety and security of participant data were ensured through use of REDCap (Research Electronic Data Capture) for administration of the study [26]. All study data were collected and managed using REDCap electronic data capture tools hosted at Vanderbilt University Medical Center. REDCap is a secure, Web-based app designed to support data capture for research studies, providing (1) an intuitive interface for validated data entry, (2) audit trails for tracking data manipulation and export procedures, (3) automated export procedures for seamless data downloads to common statistical packages, and (4) procedures for importing data from external sources.

### Participants

The email marketing service Medical Marketing Service, Inc was used to recruit physicians, nurse practitioners, and physician assistants. The lists were comprised of professional society members who had indicated oncology as an area of focus. Participants were assumed to be computer and Internet literate. Prior to enrollment, participants received the invitation email and could view the welcome page in REDCap. Copies of both are provided in Multimedia Appendix 3. To ensure that participants had been invited to take part in the study, each invitation email contained a unique 10-character alphanumeric PIN. Details related to the PINs are described in Multimedia Appendix 2. Following agreement to participate in the study, participants answered an eligibility question; participants were required to be in active practice in an oncology setting.

### Recruitment

Potential volunteers were sent email invitations to participate in the study on July 13 and 20, 2015. The second email was sent regardless of whether they had enrolled in the study or not. In order to protect participant privacy, Medical Marketing Service was not given the list of participants who had enrolled in the study. Enrollment and follow-up occurred through study close on September 7, 2015. The trial closed on September 7 because enrollments had dropped to almost zero and we believed there were no remaining active participants. After study close, we found one remaining active participant. This participant was given the gift card incentive, but the data from this participant were not used in the analysis. It was possible for participants to enroll more than once; for more information, see Multimedia Appendix 2.

### Interventions and Outcomes

The codebook for all surveys is provided in Multimedia Appendix 4. Multimedia Appendix 5 contains information about participant tracking and related analyses.

#### Learning Style Assessment

Participants were asked to self-report their learning style preferences by selecting one of the following responses to complete the statement, “I am likely to remember something a year from now if: (1) I learn it by reading, (2) I learn it by listening, (3) I learn it by watching, (4) I learn it by reading and listening, (5) I learn it by reading and watching, (6) I learn it by listening and watching, (7) I learn it by reading, listening, and watching” (see also Multimedia Appendix 4). This question was modeled after a single-item self-report measure, which was used by the authors in previous research [23]. The model self-report measure determined learning style preferences based on participants’ responses to whether they would recall how to do something a year from now if they learned it by reading, listening, watching, or trying things on their own; participants could select all that applied [23]. The revised question wording used in our study reflects that our educational content does not involve skill-based or kinesthetic learning.

#### Knowledge Tests

A 10-question test was developed to evaluate participant knowledge of the educational materials. The questions were multiple choice or true/false, and each question included a response option of “don’t know.” Participants were asked to complete the test immediately before (pretest) and after (posttest) viewing the information and 2 weeks later (follow-up test). The same questions were used for all three surveys, although the order of the questions and the order of multiple-choice answers were changed for each survey. The order of true/false answers was not changed. The surveys were loaded into REDCap and tested before study commencement. Each set of educational materials contained the answers to all 10 questions.

#### Educational Materials

New content was developed for the project for both the control and intervention arms. Copies of the educational materials are shown in Multimedia Appendix 6. Educational materials were developed by the authors, who together brought experience in the adaptation of health information according to learning preferences and in-depth knowledge of oncology [21-24,27,28]. The materials were also reviewed by two experts in breast cancer research to further ensure accuracy of the information. Intervention materials with a watching component included slides with figures, captions, and limited bulleted text. Intervention materials with a listening component included an audio recording. Intervention materials with a reading component included text. The control materials included text, figures, and tables.

#### Feedback, Demographics, and Gift Card Surveys

The feedback survey was presented to participants following the knowledge posttest. The feedback survey evaluated whether participants thought the information in the learning materials was easy to understand and if they learned something new. The survey consisted of five questions (see Multimedia Appendixes 4 and 7). Options for four of the questions were on a rating scale from strongly agree to strongly disagree (strongly agree, disagree, neither agree or disagree, agree, or strongly agree). For the fifth question, participants could share any other thoughts about the materials in an open-text field. Answering questions in this survey was optional.

The demographics survey was presented to participants following the knowledge follow-up test. For all participants, the survey consisted of six questions about their practice type (academic, community, both, or other), percentage of patients seen with breast cancer (<25%, 25%-50%, >50%, or unknown), age, gender, race, and ethnicity. Answering questions in this survey was optional.

The gift card survey followed the demographics survey. This survey collected information required for institutional financial reporting requirements and US federal statutory requirements. Although completing this survey was optional, participants could not receive the gift card incentive without completing it.

### Study Sample

The primary objective of this study was to examine the improvement of the knowledge test score (pretest vs posttest) between experimental group (matched learning materials according to learning style) and the control group (standard learning materials). According to the prospective sample size calculation, a sample size of 250 per group would provide at least 90% power to detect a conservative effect size of 0.3 with two-sided type I error of 5%. The effect size is defined as the ratio of mean difference of test score between study groups to the standard deviation.

From Medical Marketing Service, we learned that a good open rate—the likelihood that a recipient will open and view the email—for a marketing email to oncologists is 13% (Jane Stormzand, personal communication, February 24, 2015). We used this number to estimate our open rate for the invitation email. We did not have data on the level of attrition to expect during the course of the study itself, so we estimated using high and low attrition rates at 80% and 40% for each of four study events: (1) agreeing to participate in the study, (2) viewing the educational materials, (3) taking the knowledge posttest, and (4) taking the knowledge follow-up test. With a high attrition rate, we would have expected only five participants to complete the study; with a low attrition rate, we would have expected 438 to complete the study. As a result, we did not limit enrollment, and we had an IRB-approved upper limit of enrollment set at 1200. Participants who did not complete required portions of the study (learning style survey, pretest, posttest, and follow-up test) were excluded.

### Allocation Concealment and Blinding

The study was fully automated; therefore, the study personnel did not have access to the list of individuals invited to participate in the study and the study personnel had no control over when any individual clicked on the survey link in the recruitment email (the action that creates a numbered record in REDCap). For these reasons, we decided to use alternating ABAB allocation rather than randomization. Although not randomized, the allocation was concealed. Participants with odd-numbered records were allocated to the intervention arm, whereas even-numbered records were allocated to the control arm.

Although no expectations were set about the content of the educational materials before allocation or review of educational materials, we do not consider the participants to have been blinded because after clicking the submit button on the REDCap page with the link to the educational materials, participants were shown the survey queue, which listed whether the surveys were on the control or intervention arm. Study personnel were not blinded; this was deemed unnecessary because the outcome measures were objective measures—test scores—and because the study was fully automated. The study statisticians were blinded to group assignment.

### Statistical Methods

Multiple imputation was performed using the *rms* R package to account for missing data (practice type: 2.0%, 11/552; percent of breast cancer patients seen: 1.4%, 8/552; age 4.5%, 25/552; gender: 4.7%, 26/552; race: 13.9%, 77/552; physician specialty: 7.6%, 42/552). Multivariable linear regression was used to estimate the intervention effect on (1) knowledge posttest score and (2) knowledge follow-up test score, adjusted for knowledge pretest score (baseline assessment), as well as other covariates (practice type, percentage of breast cancer patients seen, age, gender, race, and physician specialty). Hierarchical cluster analysis and redundancy analysis were performed for data reduction. Years since completing residency/fellowship was dropped from the model because it could be predicted from other variables in the model. Residual analysis was used to check the linear regression assumptions of homogeneity for variance, normality, and linearity. For each individual learning style, the Wilcoxon signed rank test was performed to test for differences between (1) knowledge pretest score and knowledge posttest score, and (2) knowledge pretest score and knowledge follow-up test score. Multiple comparisons were corrected using the Bonferroni method. All tests were significant at the overall two-sided 5% level. All statistical analyses were performed in R version 3.1.2.

## Results

### Participant Flow

**Figure 2:**
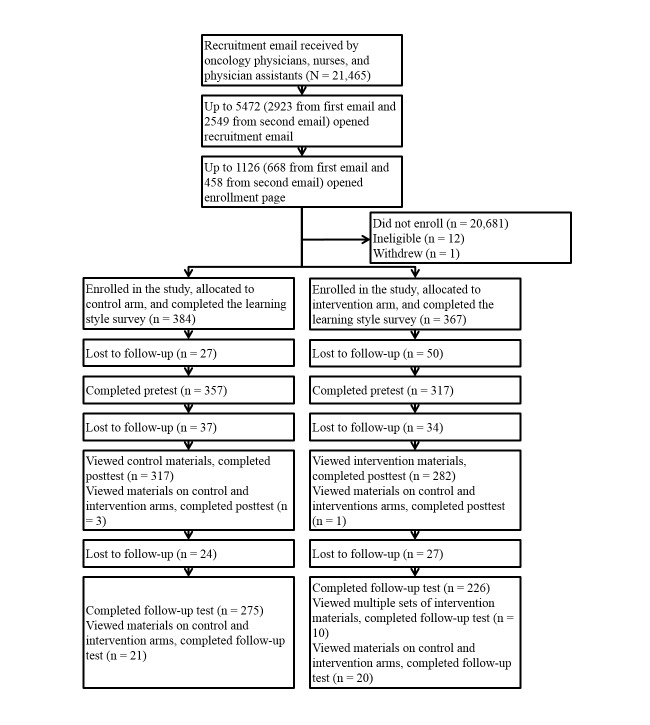
Participant flow diagram.

A total of 751 participants enrolled and completed the learning style survey (Figure 2). Of those, 384 were allocated to the control arm and 367 were allocated to the intervention arm. A total of 296 on the control arm and 256 on the intervention arm completed all required portions of the study, for a total of 552. Of those who completed the study on the control arm, 19 viewed educational materials on the control arm and the intervention arm due to multiple enrollments. Of those who completed the study on the intervention arm, 22 viewed educational materials on the control arm and the intervention arm, and 10 viewed multiple intervention arm educational materials due to multiple enrollments. Because participants could view any nonstudy materials they desired, in electronic or hard copy, and because participants were not prevented from re-enrolling to view additional on-study educational materials, participants who viewed multiple sets of educational materials were not excluded from analysis.

**Figure 3:**
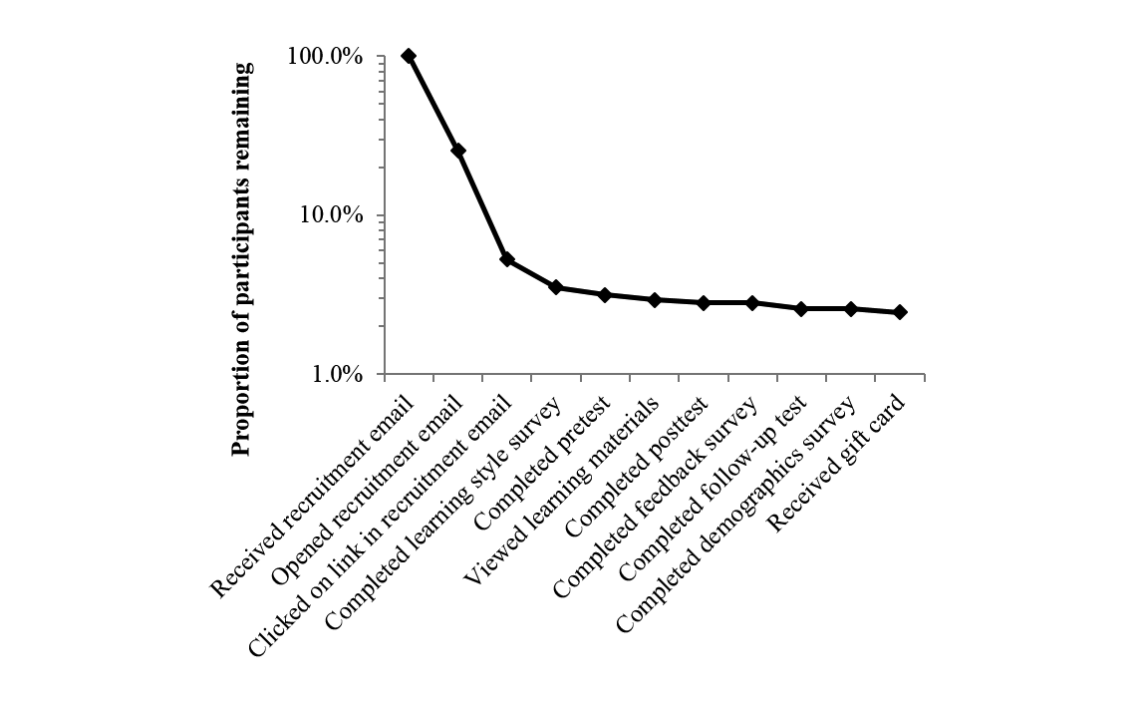
Attrition diagram.

Based on the first recruitment email, the view rate of the recruitment email itself was 13.62% (2923/21,465), and the subsequent view rate of the welcome page of the study was 22.85% (668/2923). Participation rate, determined by the ratio of unique participants completing the learning style survey to those who clicked on a link in a recruitment email, was 66.70% (751/1126). Note that this underestimates the participation rate because some participants clicked links in both recruitment emails. Completion rate, determined by the ratio of participants completing the knowledge follow-up test to those who completed the learning style survey, was 73.50% (552/751). The attrition rate decreased at each step (see Figure 3), particularly following enrollment in the study. Note that the second two events underestimate attrition; these numbers represent the sum of individuals opening and clicking links in the two recruitment emails. Because some individuals opened or clicked links in both emails, the sums overestimate participation.

We found that several PINs were used for multiple study enrollments: 552 participants enrolled once, 172 participants enrolled twice, 23 participants enrolled three times, and 4 participants enrolled four times.

### Baseline Data

Participant characteristics are shown in Table 1. Overall, the arms were well balanced for participant characteristics, with 296 participants in the control arm and 256 in the intervention arm. The majority of participants were physicians, with slightly more in the control arm versus the intervention arm (274 vs 237). There were four fewer physician assistants and one more nurse practitioner and other participant types in the intervention arm than the control arm. Half (50.6%, 274/541) of the participants in both arms worked in an academic setting, with 39.89% (215/541) working in a community setting, and 9.6% (52/541) reporting that their practice type was both academic and community based. For the majority of participants in both arms, less than 25% of patients in their practices were breast cancer patients, and for just under a third of participants, 25% to 50% of patients in their practices were breast cancer patients.

The demographics were fairly well balanced between arms; all *P* values were insignificant at the two-sided 5% significance level, as shown in Table 1. There were more men in the control arm (202 vs 163). The majority of participants in both arms were white (68%, 323/475), with an additional 25% (119/475) of participants reporting their race as Asian. The mean age was 42.8 (SD 9.8) years for the control arm and 43.3 (SD 9.9) years for the intervention arm.

More than half the participants were medical oncologists, with more in the control arm (164 vs 133). Radiation oncologists made up 15.9% (81/510) of the participants, with more in the intervention arm (35 vs 46). The number of years since physicians had completed residency/fellowship was similar in both arms, with one-third reporting less than 5 years and one-third more than 15 years.

Learning styles were also balanced between the intervention arm and the control arm. The largest group was the watching plus listening plus reading group, making up 38.8% (214/552) of participants. The next largest group was the watching plus reading group, with 18.7% (103/552) of participants selecting this group. More participants chose listening or listening plus reading in the control arm than the intervention arm (9 vs 3 for listening, 38 vs 29 for listening plus reading).

The median follow-up interval between posttest and follow-up test was equivalent between arms at 14.2 (IQR 14.0-15.8 for control arm and IQR 14.0-15.5 for intervention arm) days.

**Table 1:** Participant characteristics (N=552).

Participant characteristics		N^a^	Control (n=296)	Intervention (n=256)	*P*
**Participant type, n (%)**		552			.81^b^
	Physician		274 (92.6)	237 (92.6)	
	Physician assistant		16 (5.4)	12 (4.7)	
	Nurse and other		6 (2.0)	7 (2.7)	
**Practice type, n (%)**		541			.42^b^
	Academic	541	151 (51.9)	123 (49.2)	
	Community		109 (37.5)	106 (42.4)	
	Both		31 (10.6)	21 (8.4)	
**Percentage of patients with breast cancer seen in participant’s practice, n (%)**		544			.88^b^
	<25%		170 (58.4)	141 (55.7)	
	25%-50%		78 (26.8)	74 (29.2)	
	>50%		37 (12.7)	34 (13.4)	
	Unknown		6 (2.1)	4 (1.6)	
**Age (years)**					
	Mean (SD)	527	42.8 (9.8)	43.3 (9.9)	.57^c^
	Median (IQR)		40 (35-50)	40 (35-50)	
**Gender, n (%)**		526			.42^b^
	Male		202 (70.9)	163 (67.6)	
	Female		83 (29.1)	78 (32.4)	
**Race, n (%)**		475			.95^b^
	White		173 (67.8)	150 (68.2)	
	Asian		65 (25.5)	54 (24.6)	
	Other/multiracial		17 (6.7)	16 (7.3)	
**Hispanic, Latino, or Spanish origin, n (%)**		476			.32^b^
	Hispanic or Latino		8 (3.2)	11 (4.9)	
	Non-Hispanic		245 (96.8)	212 (95.1)	
**Physician specialty, n (%)**		510			.11^b^
	Resident		6 (2.2)	11 (4.7)	
	Fellow		39 (14.2)	20 (8.5)	
	Medical oncologist and/or hematologist		164 (59.9)	133 (56.4)	
	Surgical oncologist		10 (3.6)	9 (3.8)	
	Radiation oncologist		35 (12.8)	46 (19.5)	
	Pathologist		13 (4.7)	13 (5.5)	
	None of the above		7 (2.5)	4 (1.7)	
**Years since completed residency/fellowship, n (%)**		510			.10^b^
	<5		85 (31.1)	81 (34.2)	
	5-9		51 (18.7)	56 (23.6)	
	10-15		50 (18.3)	29 (12.2)	
	>15		64 (23.4)	60 (25.3)	
	Other		23 (8.4)	11 (4.6)	
**Follow-up interval (days)**					
	Mean (SD)	552	15.3 (3.0)	15.2 (2.5)	.58^c^
	Median (IQR)		14.2 (14.0-15.8)	14.2 (14.0-15.5)	

^a^ N is the number of nonmissing observations.

^b^ The test used was Pearson chi-square test.

^c^ The test used was Wilcoxon rank-sum test.

### Outcomes and Estimation

Analysis was conducted by original assigned groups. Almost 80% (438/552) of oncology health care professionals were multimodal, with the most common learning style being watching plus listening plus reading (Table 2).

There was a significant difference in both knowledge posttest score and knowledge follow-up test scores between the control arm and the intervention arm. The intervention arm showed a greater improvement of 0.4 points in the knowledge posttest score compared to the control group (the adjusted mean posttest scores were 7.861, SE 0.408 and 7.461, SE 0.414 for intervention arm and control arm, respectively; *P*=.004), and on average 0.3 points higher follow-up test score than the control group (the adjusted mean follow-up scores were 7.177, SE 0.400 and 6.805, SE 0.406 for the intervention arm and control arm, respectively; *P*=.02). Both analyses were adjusted for knowledge pretest score and other covariates. Variance analysis and parameter estimates for the knowledge posttest and knowledge follow-up test score regression models are shown in Multimedia Appendix 8. Among the seven individual learning styles, we detected a significant improvement in knowledge posttest score and a significant improvement in knowledge follow-up test score in all learning styles (adjusted *P* values <.001), except for watching and listening styles.

### Participant and Investigator Feedback

Table 3 shows the results of the feedback survey by study arm. Multimedia Appendix 7 shows tables and diverging stacked bar charts of the results for each question of the feedback survey by study arm and by learning style. Of the participants who completed the feedback survey, 89.7% (489/545) agreed or strongly agreed that the information was new to them, 79.9% (436/546) found it satisfying, and 78.0% (425/545) found it easy to understand. Of the 543 participants who completed the question, 174 (32.0%) agreed or strongly agreed that the information was confusing.

**Table 2:** Learning styles of oncology health care professionals.

Learning style	All, n (%) (N=552)	Control, n (%) (n=296)	Intervention, n (%) (n=256)
Watching	19 (3.4)	10 (3.4)	9 (3.5)
Listening	12 (2.2)	9 (3.0)	3 (1.2)
Reading	83 (15.0)	43 (14.5)	40 (15.6)
Watching & listening	54 (9.8)	23 (7.8)	31 (12.1)
Watching & reading	103 (18.7)	63 (21.3)	40 (15.6)
Listening & reading	67 (12.1)	38 (12.8)	29 (11.3)
Watching & listening & reading	214 (38.8)	110 (37.2)	104 (40.6)

**Table 3:** Feedback survey results.

Survey questions and responses		N	Control, n (%) (n=296)	Intervention, n (%) (n=256)
**Was the information easy to understand?**		545		
	Strongly disagree		10 (3.4)	18 (7.1)
	Disagree		19 (6.5)	13 (5.1)
	Neither disagree nor agree		41 (14.0)	19 (7.5)
	Agree		170 (58.2)	127 (50.2)
	Strongly agree		52 (17.8)	76 (30.0)
**Was the information confusing?**		543		
	Strongly disagree		20 (6.9)	39 (15.5)
	Disagree		97 (33.3)	105 (41.7)
	Neither disagree nor agree		61 (21.0)	47 (18.6)
	Agree		105 (36.1)	55 (21.8)
	Strongly agree		8 (2.8)	6 (2.4)
**Was the information satisfying?**		546		
	Strongly disagree		4 (1.4)	13 (5.2)
	Disagree		15 (5.1)	6 (2.4)
	Neither disagree nor agree		54 (18.4)	18 (7.1)
	Agree		175 (59.5)	150 (59.5)
	Strongly agree		46 (15.6)	65 (25.8)
**Was the information new?**		545		
	Strongly disagree		5 (1.7)	10 (4.0)
	Disagree		1 (0.3)	4 (1.6)
	Neither disagree nor agree		25 (8.5)	11 (4.4)
	Agree		173 (59.0)	127 (50.4)
	Strongly agree		89 (30.4)	100 (39.7)

Most positive comments on the feedback survey dealt with the educational materials: participants found them to be good, useful, organized, evidence-based, important, well designed, succinct, easy to understand, interesting, enjoyable, and exciting. Others appreciated the provision of a knowledge pretest or said that the information would help them explain treatment regimens to patients. We received an email from a colleague of a participant expressing interest in related studies. We received requests for the educational materials to be made available to participants following the study.

Most negative comments on the feedback survey also dealt with the educational materials: some found them to be poorly organized, busy, or confusing. Eighteen respondents suggested adding figures, tables, or videos. Of these, 14 participants had been allocated to the control arm. These participants were generally multimodal learners who perceived My Cancer Genome content as being text-based and thus lacking in graphical elements. The other four participants were allocated to the intervention arm. These participants were listening, reading, and listening plus reading learners; they each requested visual educational materials. Others felt the information was not up to date.

## Discussion

The first objective of this study was to measure learning styles of oncology health care professionals. Compared to assessments of learning styles of medical students and allied health students, this cohort has a higher percentage of multimodal learners at 79.3% (436/552); those studies found rates of 61% (61/100) and 66% (90/137), respectively. The comparison is not direct because this study did not include kinesthetic as a learning style option and because this study used self-reporting to assess learning style [15,29].

The second objective of the study was to determine whether learning style-tailored learning materials fostered greater learning and retention than typical My Cancer Genome content. Although participants did learn more when viewing educational materials tailored to learning style, the mean benefit was only 0.40 points on the knowledge test. An opportunity for future research that would permit a conclusion as to whether the use of learning style-tailored materials facilitate learning in general would use materials inconsistent with learning style preferences rather than the standard format used in our study as the control [30]. However, care would need to be taken to account for demand characteristics, especially due to the higher level of scientific literacy of participants and the likelihood that the learning style survey at the beginning would provide a cue regarding the hypothesis being tested [31]. Instead, by providing control arm participants with access to My Cancer Genome standard content, some participants on the control arm received matched-style content, whereas others received mismatched style content. This may have reduced demand characteristics that would skew the control arm to poorer performance. Finally, a limitation of the study was that we did not assess how the quality of the materials may have impacted learning.

Several participants requested more visual elements to be incorporated into the control educational materials of typical My Cancer Genome content and into nonvisual educational materials in the intervention arm, even when those participants did not identify themselves as watching learners. Together, these participant comments, the high percentage (79.3%, 436/552) of multimodal learners, and the small but significant improvement in knowledge scores when presented with materials tailored to learning style highlight the need for Web-based educational materials to address watching, listening, and reading learners. Therefore, it will be important for My Cancer Genome to incorporate more watching and listening elements, including graphics and videos, in particular.

Given the relatively small improvement in test scores and the heavy resource demand required to create separate content for each learning style, it is probably a poor use of resources to generate new content for each learning style. Instead, the creation and embedding of graphical and video content to address multimodal learning styles may enhance current My Cancer Genome pages, address the learning gap for watching, listening, and multimodal learners, and provide the oncology community with resources for presentations and additional dissemination of learning materials. To this end, since the completion of this study, My Cancer Genome has begun to enhance its website with multimodal content. My Cancer Genome has improved its visual representations of molecular pathways involved in cancer, embedded graphics in new content related to diseases such as chronic lymphocytic leukemia and in new content related to new genetic testing modalities, such as digital droplet polymerase chain reaction, and embedded graphical-audio “knowledge pearls” explaining key concepts such as “mutation” [27]. Additionally, as a result of this study, My Cancer Genome is in the process of creating a 2.0 version of the My Cancer Genome website, which will heavily rely on multimodal data presentation to convey curated knowledge content.

In conclusion, the results of this study imply that Web-based educational materials should be multimodal: (1) most oncology health care professionals are multimodal learners, (2) the increase in learning when learning style-tailored educational materials were used is small, and (3) multiple requests for more multimodal materials were received from participants in both the control and intervention arms. These conclusions are corroborated by a significant body of evidence and confirmed by this work for oncology health care professionals [30].

## Appendix

Multimedia Appendix 1 CONSORT-EHEALTH checklist.

Multimedia Appendix 2 Technical Details and Changes After Study Commencement.

Multimedia Appendix 3 Emails Sent to Participants.

Multimedia Appendix 4 Codebook for All Surveys.

Multimedia Appendix 5 Participant Tracking and Related Analyses.

Multimedia Appendix 6 Educational Materials.

Multimedia Appendix 7 Feedback Survey Results.

Multimedia Appendix 8 Variance Analysis and Parameter Estimates.

## Abbreviations

CDK4: cyclin-dependent kinase 4 protein
CDK6: cyclin-dependent kinase 6 protein
CONSORT: Consolidated Standards of Reporting Trials
EGFR: epidermal growth factor receptor
ER+: estrogen receptor positive
IRB: institutional review board
PIN: personal identification number
REDCap: Research Electronic Data Capture
