# Supplementary material for: Internet-Based Assessment of Oncology Health Care Professional Learning Style and Optimization of Materials for Web-Based Learning: Controlled Trial With Concealed Allocation
Source: J Med Internet Res. 2017 Jul 25;19(7):e265. doi: 10.2196/jmir.7506 (PMC5548983; doi:10.2196/jmir.7506)
Supplement: Multimedia Appendix 2 [file jmir_v19i7e265_app2.pdf]

Multimedia Appendix 2

Technical Details and Changes After Study Commencement

Technical Details

Each participant who completed the study viewed eight survey instruments and one set of educational materials. All surveys were developed by project team members SK, IA, PL, KJ, CM, and ML. The project team included members from Knowledge Management and My Cancer Genome at Vanderbilt University Medical Center. The Knowledge Management team provided expertise in the customization of health information according to learning styles [1-3]. Three members from Knowledge Management contributed to the project: two with masters-level training in information and/or library science, of which one had more than 10 years of experience providing evidence for complex clinical questions and quality reviews for the Breast Tumor Board, and one member with a PhD in the basic sciences. The My Cancer Genome team provided cancer content expertise and included three members from My Cancer Genome: two with PhDs in the basic sciences and one with an MD/PhD specializing in oncology and informatics.

Use of personal identification numbers (PINs) ensured integrity of the invitation and participation processes. PINs were generated using the Random String Generator at the Randomness and Integrity Services Ltd. website [4]. By appending URL parameters to the link to the study's welcome page, we pre-filled the PINs into the initial form so that the risk of copying errors was minimized. REDCap is not able to pre-populate participant records by PIN. Instead, the study was accessed by a public link; the disadvantage of this mechanism was that participants could enroll with the same PIN more than once. However, the PIN enabled us to determine not only whether participants had been invited to participate, but also whether a single invitation was used for multiple enrollments into the study. Participant IP addresses were not tracked, and log files were not analyzed.

Everyone recruited received two invitation emails. Participants who completed the study received between four and seven emails (Table 1). If participants enrolled more than once, they received emails associated with each enrollment. Participants may also have elected to receive an email from REDCap if they chose to save and return to the survey later; we did not track these emails. Help and other support were available only through the study personnel via email or phone. Communications of this type are described in the results. Study personnel did not interact with the participants in any way except as contacted by participants via email or phone as described in the results. Only CMM responded to participants inquiries. CMM and IAA sent out the Amazon.com Gift Cards. CMM was the study coordinator and is the managing editor for My Cancer Genome. IAA is the program coordinator for My Cancer Genome. Beyond the steps triggered by the emails just described, REDCap directed participants from one survey to the next. Table 2 describes how participants were directed through the study by REDCap.

Table 1. Emails Sent to Study Participants.

Email Purpose	Timing	Reminders

Backup link to educational materials and invitation to complete knowledge posttest	Sent upon completion of knowledge pretest	None
Invitation to knowledge follow-up test	Sent 14 days after completion of knowledge posttest	Once every 2 days up to 3 times
Confirmation of completion of the Amazon.com Gift Card survey	Sent upon completion of the Amazon.com Gift Card survey	None
Email with the Amazon.com Gift Card	Sent manually following confirmation of unique participant and NPI, if necessary	None

Table 2. Mechanics of REDCap Used to Direct Participants Through Study.

Instrument	Mechanism	Trigger	Result
Learning Style Survey	Public URL with parameters to pre-fill participant PIN	Upon clicking the link in one of the recruitment emails	Learning style survey is displayed
	End survey command	Answer of "no" to question, "I agree to participate in this study" (consent)	Goodbye message and study end
	Branching logic	Answer of "no" to question, "Are you actively practicing in an oncology setting?" (active practice)	Ineligibility message and study end
Knowledge Pretest	Survey queue[a]	Upon completion of learning style survey, answer of "yes" to consent and active practice questions	If record ID was even, allocation to control arm. If record ID was odd, allocation to intervention arm. In both cases, participants were automatically directed to the knowledge pretest
Link to Educational Materials	Survey queue	Upon completion of the knowledge pretest	Participants automatically directed to display of link to educational materials.
	Survey completion	Upon clicking the submit button in the link to	Survey completion text with back-up link to educational

	text and survey queue	educational materials instrument	materials and directions on how to access knowledge posttest are provided, along with a list of the instruments viewed so far. A link is provided to the knowledge posttest
Knowledge Posttest	Automated invitations	Upon completion of knowledge pretest	Email with link to educational materials and to the knowledge posttest
	Participant action	Upon clicking the link to the knowledge posttest in the link to educational materials survey completion text or the automated invitation to the knowledge posttest	Knowledge posttest is displayed
Feedback Survey	Survey queue	Upon completion of the knowledge posttest	Participants automatically directed to display of feedback survey
	Survey completion text	Upon clicking the submit button in the feedback survey	Survey completion text explaining that the link to the knowledge follow-up test would arrive by email in two weeks is displayed
Knowledge Follow-Up Test	Automated invitations	Two weeks after completion of knowledge posttest	Email with link to knowledge follow-up test
Demographics Survey	Survey queue	Upon completion of knowledge follow-up test	Participants automatically directed to display of demographics survey
Amazon Gift Card Survey	Survey queue	Upon completion of demographics survey	Participants automatically directed to display of Amazon gift card survey

NOTE: [a] In REDCap, the survey queue tool is a list of surveys, grouped by study arm, with the ability to define conditions upon which a survey appears, as well as whether the survey starts automatically or not.

Adaptive questioning was used in the learning style survey, demographics survey, and Amazon gift card survey. These surveys used branching logic to limit the number of questions posed to participants on the basis of their eligibility and practice type. So, for example, only physicians were asked for specialty, years since residency, and for National Provider Identifier (NPI). Table 3 shows the number of pages and the number of items per instrument, based on an

eligible physician participant. Emails sent during the course of the study are not included in Table 3. An item is defined as asking for a response from the participant, including submit buttons; text-only components are not counted in Table 3. Some items were required, and REDCap would not permit surveys to be submitted until all required items were completed. Participants could change answers until they submitted each page. Participants could save and return to the surveys later; while we did not allow participants to change previously saved answers per REDCap settings, we found during testing that it was generally possible. This bug was rarely if ever exploited, based on the low number of return codes generated. Participants wanting to change answers could also re-enroll in the study; subsequent submissions of each survey were not evaluated, however.

Table 3. Number of Pages and Items Per Page Based on an Eligible Physician Participant.

Instrument	Page # (cumulative)	Items (cumulative)
Learning Style Survey	1 (1)	4 (4)
	2 (2)	2 (6)
	3 (3)	4 (10)
	4 (4)	2 (12)
Knowledge Pretest	1 (5)	11 (23)
Link to Educational Materials	1 (6)	1 (24)
	2 (7)	1 (25)
Knowledge Posttest	1 (8)	11 (36)
Feedback Survey	1 (9)	6 (42)
	2 (10)	0 (42)
Knowledge Follow-Up Test	1 (11)	11 (53)
Demographics Survey	1 (12)	7 (60)
Amazon Gift Card Survey	1 (13)	14 (74)
	2 (14)	0 (74)

Use of the study surveys and educational materials was monitored via user records in REDCap and via Google Analytics. For the educational materials, cookies were used to track participant usage.

All data were input into REDCap by individual participants. Study personnel did not interact with participants during the study unless the participant called or emailed study personnel. Most email and phone communication that occurred prior to a participant's study completion related to whether and when the invitation to the follow-up test would arrive; some of these were participants confused by the second recruitment email. Most email and phone communication that occurred after a participant's study completion related to whether and when the Amazon.com Gift Card incentive would arrive. All queries were resolved to the satisfaction of the study participants.

In addition to the knowledge tests, qualitative feedback was obtained from participants following the knowledge posttest. Demographic information was collected from participants during 1) the initial survey as part of determining eligibility and 2) the demographics survey. Finally, identifying information was collected from participants who elected to receive the $100 Amazon.com Gift Card incentive.

No changes were made to study outcome measures after study commencement.

No major secular events such FDA approval of a CDK4/6 inhibitor, release of a major new internet educational resource on CDK4/6 inhibitors, or major changes to delivery of internet hardware or delivery resources [5], fell in the study period.

Changes After Study Commencement

There was no downtime of the study between study opening and study closing. The study experienced a few minor bugs. An image and link to Vanderbilt University in the footer of the read/write learning style educational material became non-functional while the study was open. It was discovered on July 28, 2015 and logged in our technical project management system. The issue was repaired and verified in production on August 5, 2015. Also, there was a bug in REDCap that caused the first approximately 200 participants' follow-up invitations and reminders to be lost. The bug was fixed by the REDCap team and did not affect the remaining participants. For those affected, invitations and reminders were rescheduled manually. A change we made also resulted in the need to manually reschedule follow-up survey invitations and reminders: we revised the wording of the follow-up survey invitation, to indicate that if the study accrual limit was reached it would be closed. To make this change, we had to manually delete and reschedule the follow-up survey invitations and reminders for the first approximately 530 participant enrollments (out of 1,032 total enrollments). In both cases, the effect that this had was that the invitations (sent two weeks after completion of the knowledge posttest) and reminders (every other day following the follow-up invitation until the follow-up survey was completed or after 6 days) were sent at noon instead of at the time the knowledge posttest was completed. There were eight revisions to the project between July 13 and September 7; of these, six were for the addition of and changes to a data form to track gift card information, for use by study personnel. The form tracked PINs, duplicate entries, NPI verification, and the ability to add the business address from the NPI database (some participants provided their home addresses so for Sunshine Act reporting purposes we needed to record the publicly reported business address). These revisions did not affect any part of the surveys taken by the participants. For the remaining two changes, the first added questions to the gift card survey asking whether the participant was a Vanderbilt employee. We were required by Vanderbilt to collect social security numbers from Vanderbilt employees. For the second, we added questions to collect addresses from participants as required for tracking by the Vanderbilt University Finance Department. Both of these changes were made before any participants viewed the gift card survey, and so all participants saw the same survey.

Development of Educational Materials

Development of Intervention Materials

Literature was identified using PubMed, searches of abstracts from professional meetings—including the San Antonio Breast Cancer Symposium—and searches of pathway-specific clinical trials using the ClinicalTrials.gov website. As appropriate, publicly available data sets were used, including the NCBI Gene database [6], Uniprot [7], Drugs@FDA [8], the NCI's Physician Data Query database [9], ClinicalTrials.gov [10], GeneNames.org [11,12], RefSeq [13,14], Ensembl [15,16], and others.

An initial written draft of the content was created, and images were developed based on this draft. The team consulted the literature to identify standardized ways that biological processes, pathways, and drug mechanisms were depicted. The primary literature, review

articles, and the gray literature, including accredited CME presentations, were consulted. Based on this review, images were created in PowerPoint.

The information was then adapted according to preferences for learning via different sensory modalities [17-19]. For reading learners, a text document was created using Word. The watching version of the information was developed using PowerPoint in order to facilitate the incorporation of images. An audio version of the information was developed for listening learners using a Sony ICD-UX533 recorder and edited in Audacity 2.1.0 sound editing software. These materials were then combined to address multimodal learning styles, and some elements of the educational modules were modified for specific learning styles (Table 4).

Table 4. Learning Style-Tailored Educational Material Format.

Learning preference	Format
Watching	Slides (v.1)
Listening	Audio (v.1)
Reading	Text (v.1)
Watching & listening	Slides (v.1) with audio (v.2)
Watching & reading	Slides (v.1) with text (v.2)
Listening & reading	Audio (v.1) with text (v.1)
Watching & listening & reading	Slides (v.1) with audio (v.2) and text (v.2)

Participants could access the materials using two routes: through a link provided on a screen that appeared after completion of the knowledge pretest or through a link provided in an automated email sent after completion of the knowledge pretest. The REDCap website is responsive, and so participants could participate on any computer, tablet, or mobile device. The educational materials were also hosted on a responsive platform, so every part of the study could be completed from any device.

Our intention was for the educational materials to be viewed by the participants for about 10-15 minutes. The only information participants received about how much time to expect to use the materials was the estimated time commitment for the study on the welcome screen of the study.

Participants directed to the control materials on My Cancer Genome and to the reading and reading plus listening intervention materials were provided with a banner under the header stating that when they were finished reviewing the materials, they should return to REDCap to take the knowledge posttest. Participants directed to the listening, watching, listening plus watching, reading plus watching, and listening plus reading plus watching intervention materials received an initial slide explaining the program navigation buttons (e.g., play, skip, back, etc.), and a closing slide reminding participants to return to REDCap to take the knowledge posttest.

Development of Control Materials: My Cancer Genome Pages

Two new page types were developed for My Cancer Genome webpages (Multimedia Appendix 5). The first was a drug-class-in-disease page type; specific pages created included pages on CDK4/6 inhibition and CDK4/6 inhibitors in breast cancer and on mTOR inhibition and mTOR inhibitors in breast cancer. These pages included information about the mechanism of

action of the drug class and identified drugs with FDA approval or in clinical trials. The second type of new page was a drug-in-disease page. These pages each contained information about a single drug in breast cancer. These drugs were the CDK4/6 inhibitors palbociclib, ribociclib, and abemaciclib and the mTOR inhibitor everolimus. For each drug-in-disease page, information about the developmental status, information about the drug approval status, reported results from clinical trials (abstracts or publications), and ongoing and recruiting clinical trials with the drugs in breast cancer were summarized.

Technical Design, Testing, and Display of Learning Style-Tailored Educational Content

The seven learning-style-tailored sets of educational materials were sent to Danny Wenner, a developer in the Vanderbilt-Ingram Cancer Center Research Informatics Core, in the form of PowerPoint presentation slides, MP3 audio, and text files. DW, using Adobe Captivate, then combined the necessary components for each learning style, ensuring to normalize audio levels and match up slides to voice-over where necessary. He then exported each presentation as a JavaScript project wherein the slides were converted to JPG images, and the normalized audio was exported as MP3 files.

DW then gave the exported project files to Ross Oreto, also a developer within Vanderbilt-Ingram Cancer Center Research Informatics Core, who implemented a new controller in the My Cancer Genome PHP web application to serve each presentation to client browsers. Some manipulation of timestamps and event listeners were required to ensure compatibility with as many different browsers as possible. RO also implemented a cookie system that would allow participant traffic and click-through tracking using the Event system in Google Analytics.

My Cancer Genome is hosted on Red Hat Enterprise Linux 5 virtual machines. The Vanderbilt-Ingram Cancer Center team used an iterative release process of short sprints in order to develop, test, deploy, and verify that the presentations were accurate and easy to access by the end-user.

Abbreviations

CDK4: cyclin-dependent kinase 4 protein
CDK6: cyclin-dependent kinase 6 protein
CME: continuing medical education
FDA: U.S. Food and Drug Administration
JPG: Joint Photographic Experts Group
MP3: Moving Picture Experts audio layer III
mTOR: serine/threonine-protein kinase mTOR
NCBI: National Center for Biotechnology Information
NCI: National Cancer Institute
NPI: National Provider Identifier
PIN: personal identification number
PHP: PHP: Hypertext Preprocessor
REDCap: Research Electronic Data Capture

References

1. Giuse NB, Koonce TY, Storrow AB, Kusnoor SV, Ye F. Using health literacy and learning style preferences to optimize the delivery of health information. J Health Commun; 2012;17 Suppl 3:122-140. doi:10.1080/10810730.2012.712610
2. Koonce TY, Giuse NB, Choemprayong S, Martin SL, Epelbaum M, Kusnoor SV. Using personalized education delivery to improve community clinic patients' knowledge of hypertension. Special Libraries Association Annual Meeting, Biomedical & Life Sciences Division. 2013; San Diego, CA.
3. Koonce TY, Giuse NB, Kusnoor SV, Hurley S, Ye F. A personalized approach to deliver health care information to diabetic patients in community care clinics. J Med Libr Assoc; 2015;103(3):123-130. doi:10.3163/1536-5050.103.3.004
4. Haahr M, Haahr S. 2015. Random string generator. https://www.random.org/strings/. Archived at: http://www.webcitation.org/6gQJOrhAS
5. Baker M, Gustafson DH, Shaw B, Hawkins R, Pingree S, Roberts L, Strecher V. Relevance of CONSORT reporting criteria for research on eHealth interventions. Patient Educ Couns; 2010; 81S1:S77-S86. doi:10.1016/j.pec.2010.07.040
6. National Center for Biotechnology Information, U.S. National Library of Medicine, National Institutes of Health. 2016. Gene database. https://www.ncbi.nlm.nih.gov/gene. Archived at: http://www.webcitation.org/6gcdjzkBq
7. UniProt Consortium. 2016. UniProt. http://www.uniprot.org/. Archived at: http://www.webcitation.org/6gce1fimJ
8. Center for Drug Evaluation and Research, U.S. Food and Drug Administration. 2016. Drugs@FDA. https://www.accessdata.fda.gov/scripts/cder/drugsatfda/. Archived at: http://www.webcitation.org/6gce7prJQ
9. National Cancer Institute. 2016. Physician Data Query Database. http://www.cancer.gov/publications/pdq. Archived at: http://www.webcitation.org/6gcer2SXH
10. U.S. National Library of Medicine, National Institutes of Health. 2016. ClinicalTrials.gov: A service of the U.S. National Institutes of Health. https://clinicaltrials.gov/. Archived at: http://www.webcitation.org/6gQMWz70I
11. HUGO Gene Nomenclature Committee. 2016. genenames.org. http://www.genenames.org/. Archived at: http://www.webcitation.org/6gceHYhLM
12. Gray KA, Yates B, Seal RL, Wright MW, Bruford EA. Genenames.org: the HGNC resources in 2015. Nucleic Acids Res; 2015;43(Database issue):D1079-1085. doi:10.1093/nar/gku1071
13. National Center for Biotechnology Information, U.S. National Library of Medicine. 2016. RefSeq: NCBI Reference Sequence Database. http://www.ncbi.nlm.nih.gov/refseq/. Archived at: http://www.webcitation.org/6gceQ7v0l
14. Pruitt KD, Brown GR, Hiatt SM, Thibaud-Nissen F, Astashyn A, Ermolaeva O, Farrell CM, Hart J, Landrum MJ, McGarvey KM, Murphy MR, O'Leary NA, Pujar S, Rajput B, Rangwala SH, Riddick LD, Shkeda A, Sun H, Tamez P, Tully RE, Wallin C, Webb D, Weber J, Wu W, DiCuccio M, Kitts P, Maglott DR, Murphy TD, Ostell JM. RefSeq: an update on mammalian reference sequences. Nucleic Acids Res; 2014;42(Database issue):D756-763. doi:10.1093/nar/gkt1114

15. EMBL-EBI, Wellcome Trust Sanger Institute. 2016. Ensembl. http://useast.ensembl.org/index.html. Archived at: http://www.webcitation.org/6gcebwEhU
16. Herrero J, Muffato M, Beal K, Fitzgerald S, Gordon L, Pignatelli M, Vilella AJ, Searle SM, Amode R, Brent S, Spooner W, Kulesha E, Yates A, Flicek P. Ensembl comparative genomics resources. Database (Oxford); 2016;2016. doi:10.1093/database/bav096
17. Good JP, Ramos D, D'Amore DC. Learning style preferences and academic success of preclinical allied health students. J Allied Health; 2013;42(4):e81-90.
18. Kharb P, Samanta PP, Jindal M, Singh V. The learning styles and the preferred teaching-learning strategies of first year medical students. J Clin Diagn Res; 2013;7(6):1089-1092. doi:10.7860/JCDR/2013/5809.3090
19. Prithishkumar IJ, Michael SA. Understanding your study: Using the VARK model. Education Forum; 2014;60(2):183-186. doi:10.4103/0022-3859.132337
