# Supplementary material for: Internet-Based Assessment of Oncology Health Care Professional Learning Style and Optimization of Materials for Web-Based Learning: Controlled Trial With Concealed Allocation
Source: J Med Internet Res. 2017 Jul 25;19(7):e265. doi: 10.2196/jmir.7506 (PMC5548983; doi:10.2196/jmir.7506)
Supplement: Multimedia Appendix 4 [file jmir_v19i7e265_app4.pdf]

Multimedia Appendix 4

Codebook for all surveys

Includes all branching logic.

#	Variable / Field Name	Field Label<br>*Field Note*	Field Attributes (Field Type, Validation, Choices, Calculations, etc.)
Instrument: **Learning Style Survey**			
1	record_id	Record ID	text
2	introduction	The My Cancer Genome team at Vanderbilt-Ingram Cancer Center (VICC) is conducting a study to improve cancer genetics web pages for oncology physicians and nurses. You are invited to participate in this oncology education program and survey about the concepts of sensitivity and resistance to targeted therapies for ER+ breast cancer.<br><br>Here's how it works:<br>-- Answer a question about your preferred learning format and take a 10-question pre-test.<br>-- Take a look at the educational materials and answer a few questions.<br>-- In two weeks, answer a few follow-up questions, and fill out a form to receive your $100 Amazon.com Gift Card.*<br><br>Participating will take about 30 minutes. Your participation is totally voluntary and all responses will be confidential. There is very low risk to participating in this survey. You can choose not to answer all of the questions.<br><br>Thank you so much for participating. If you have questions or comments contact us via email at mialevy.mycancergenome@vanderbilt.edu or by phone by calling Christine Micheel, Ph.D. (study coordinator and My	descriptive

		Cancer Genome managing editor), at 615-933-9368.<br><br>Thank you,<br><br>Mia Levy, MD, PhD<br><br>Co-Editor in Chief, My Cancer Genome<br>Assistant Professor of Biomedical Informatics and Medicine<br>Vanderbilt University Medical Center<br>2220 Pierce Avenue<br>691 Preston Research Building<br>Nashville, TN 37232-6838	
3	pin_number	What is the PIN number from your invitation email?<br>*This 10-character code can be found in your invitation email. It is case sensitive. Please note that entries associated with incorrect PIN numbers will be ineligible for the $100 Amazon gift card.*	text, Required
4	email	What is your email address?<br>*Your email is required to complete the study and for your gift card. Your email will not be used for anything other than the study or after completion of the study.*	text (email), Required, Identifier
5	consent	I agree to participate in this study.	yesno, Required<br>1 Yes<br>0 No<br><br>Stop actions on 0
6	amazon_disclaimer_1	*Amazon.com is not a sponsor of this promotion. Except as required by law, Amazon.com Gift Cards ("GCs") cannot be transferred for value or redeemed for cash. GCs may be used only for purchases of eligible goods at Amazon.com or certain of its affiliated websites. For complete terms and conditions, see www.amazon.com/gc-legal. GCs are issued by ACI Gift	descriptive

		Cards, Inc., a Washington corporation. All Amazon ®, ™ & © are IP of Amazon.com, Inc. or its affiliates. No expiration date or service fees.	
7	active_practice	Are you actively practicing in an oncology setting?	yesno, Required<br>1 Yes<br>0 No
8	goodbye<br>Show the field ONLY if:<br>[active_practice] = '0'	Only individuals actively practicing in an oncology setting are eligible to participate in this study. Thank you for your interest.	descriptive
9	role_description<br>Show the field ONLY if:<br>[active_practice] = '1'	Which of the following best describes you?	radio<br>1 Physician<br>2 Physician Assistant<br>3 Nurse<br>4 Genetic Counselor<br>5 Allied Health Provider<br>6 Other
10	other_role<br>Show the field ONLY if:<br>[role_description] = '6'	Please explain:	text
11	physician_specialty<br>Show the field ONLY if:<br>[role_description] = '1'	Which of the following best describes you?	radio<br>1 Resident<br>2 Fellow<br>3 Medical oncologist and/or hematologist<br>4 Surgical oncologist<br>5 Radiation oncologist<br>6 Pathologist<br>7 None of the above
12	md_none_of_the_above<br>Show the field ONLY if:<br>[physician_specialty] = '7'	If you marked none of the above, please explain.	text
13	residency_fellowship<br>Show the field ONLY if:<br>[role_description] = '1'	How many years has it been since you completed residency/fellowship?	radio<br>1 Less than 5<br>2 5-9<br>3 10-15<br>4 Greater than 15

			5 N/A
14	learning_styles<br>Show the field ONLY if:<br>[active_practice] = '1'	I am likely to remember something a year from now if:	radio, Required<br>100 I learn it by reading<br>010 I learn it by listening<br>001 I learn it by watching<br>110 I learn it by reading and listening<br>101 I learn it by reading and watching<br>011 I learn it by listening and watching<br>111 I learn it by reading, listening, and watching
15	learning_style_survey_complete	Complete?	dropdown<br>0 Incomplete<br>1 Unverified<br>2 Complete
Instrument: **Knowledge Pre-Test**			
16	pretest_rb1	Phosphorylation of RB1 facilitates which of the following:	radio, Required<br>20 Tumor suppression<br>21 Inhibition of cell cycle progression<br>22 Entry into the S phase of the cell cycle<br>23 Formation of a Cyclin D1-CDK4/6 complex<br>100 Don't know
17	pretest_acquired_definition	Acquired resistance to endocrine therapy in ER+ breast cancer is defined as recurrence at least 6-12 months after completion of adjuvant therapy or disease progression > 6 months after endocrine therapy initiated in the metastatic setting.	radio, Required<br>1 True<br>0 False<br>100 Don't know
18	pretest_primary_definition	Please fill in the blank with the correct answer. Primary resistance to endocrine therapy in ER+ breast cancer has been defined as disease progression less than ____ months after treatment in the metastatic setting.	radio, Required<br>12 12<br>10 10<br>8 8<br>6 6

<table>
<tr><td></td><td></td><td></td><td><table><tr><td>100</td><td>Don't know</td></tr></table></td></tr>
<tr><td>19</td><td>pretest_estrogen</td><td>Estrogen is a steroid hormone that controls cell processes like cell division, growth, differentiation, and proliferation.</td><td>radio, Required<table><tr><td>1</td><td>True</td></tr><tr><td>0</td><td>False</td></tr><tr><td>100</td><td>Don't know</td></tr></table></td></tr>
<tr><td>20</td><td>pretest_primary_mechanisms</td><td>Which of the following is a way primary resistance to endocrine therapy in estrogen receptor-positive (ER+) breast cancer may occur?</td><td>radio, Required<table><tr><td>30</td><td>Estrogen receptor (ER) amplification</td></tr><tr><td>31</td><td>Cyclin D1 amplification</td></tr><tr><td>32</td><td>RB1 amplification</td></tr><tr><td>300</td><td>None of the above</td></tr><tr><td>100</td><td>Don't know</td></tr></table></td></tr>
<tr><td>21</td><td>pretest_acquired_mechanisms</td><td>Which of the following are ways acquired resistance to endocrine therapy in ER+ breast may occur?</td><td>radio, Required<table><tr><td>40</td><td>Activation of growth factor signaling pathways</td></tr><tr><td>41</td><td>Changes in the tumor microenvironment</td></tr><tr><td>42</td><td>Estrogen receptor mutations</td></tr><tr><td>200</td><td>All of the above</td></tr><tr><td>100</td><td>Don't know</td></tr></table></td></tr>
<tr><td>22</td><td>pretest_fda_approved</td><td>Which drug combination is FDA-approved to treat ER+ metastatic breast cancer resistant to letrozole or anastrazole?</td><td>radio, Required<table><tr><td>50</td><td>Everolimus + exemestane</td></tr><tr><td>51</td><td>Erlotinib + exemestane</td></tr><tr><td>52</td><td>Palbociclib + exemestane</td></tr><tr><td>53</td><td>Bevacizumab + exemestane</td></tr><tr><td>100</td><td>Don't know</td></tr></table></td></tr>
<tr><td>23</td><td>pretest_cdk46_settings</td><td>CDK4/6 inhibitors have only been evaluated in the first line setting in breast cancer.</td><td>radio, Required<table><tr><td>1</td><td>True</td></tr><tr><td>0</td><td>False</td></tr><tr><td>100</td><td>Don't know</td></tr></table></td></tr>
<tr><td>24</td><td>pretest_fda_accelerated</td><td>The FDA granted accelerated approval of which of the following drug combinations for first line or initial endocrine therapy in postmenopausal women with ER+/HER2-negative advanced breast cancer?</td><td>radio, Required<table><tr><td>60</td><td>Palbociclib + letrozole</td></tr><tr><td>61</td><td>Erlotinib + letrozole</td></tr><tr><td>62</td><td>Bevacizumab + letrozole</td></tr><tr><td>63</td><td>Everolimus + letrozole</td></tr></table></td></tr>
</table>

			100 Don't know
25	pretest_palbociclib	Palbociclib is in which of the following drug classes?	radio, Required<br>70 mTOR inhibitors<br>71 CDK4/6 inhibitors<br>72 PI3K inhibitors<br>73 EGFR inhibitors<br>100 Don't know
26	prelearning_material_review_survey_complete	Complete?	dropdown<br>0 Incomplete<br>1 Unverified<br>2 Complete
Instrument: **Link to Learning Materials**			
27	learning_materials_link	Please click on the link below to view your learning materials:<br><br>Link to Learning Materials (http://www.mycancergenome.org/learning-material/?record=[enrollment_arm_1][record_id]&ls=[enrollment_arm_1][learning_styles]&pin=[enrollment_arm_1][pin_number])<br><br>When you are finished reviewing your learning materials, click the submit button below. You will be taken to a page with a link to the "Post-Learning Material Review Survey."<br><br>You should also be receiving this link and a link to the next survey by email.	descriptive
28	link_to_learning_materials_complete	Complete?	dropdown<br>0 Incomplete<br>1 Unverified<br>2 Complete
Instrument: **Knowledge Post-Test**			
29	posttest_palbociclib	Palbociclib is in which of the following drug classes?	radio, Required<br>72 PI3K inhibitors

			70 mTOR inhibitors<br>71 CDK4/6 inhibitors<br>73 EGFR inhibitors<br>100 Don't know
30	posttest_cdk46_settings	CDK4/6 inhibitors have only been evaluated in the first line setting in breast cancer.	radio, Required<br>1 True<br>0 False<br>100 Don't know
31	posttest_acquired_mechanisms	Which of the following are ways acquired resistance to endocrine therapy in ER+ breast may occur?	radio, Required<br>42 Estrogen receptor mutations<br>40 Activation of growth factor signaling pathways<br>41 Changes in the tumor microenvironment<br>200 All of the above<br>100 Don't know
32	posttest_acquired_definition	Acquired resistance to endocrine therapy in ER+ breast cancer is defined as recurrence at least 6-12 months after completion of adjuvant therapy or disease progression > 6 months after endocrine therapy initiated in the metastatic setting.	radio, Required<br>1 True<br>0 False<br>100 Don't know
33	posttest_fda_approved	Which drug combination is FDA-approved to treat ER+ metastatic breast cancer resistant to letrozole or anastrazole?	radio, Required<br>53 Bevacizumab + exemestane<br>50 Everolimus + exemestane<br>52 Palbociclib + exemestane<br>51 Erlotinib + exemestane<br>100 Don't know
34	posttest_fda_accelerated	The FDA granted accelerated approval of which of the following drug combinations for first line or initial endocrine therapy in postmenopausal women with ER+/HER2-negative advanced breast cancer?	radio, Required<br>63 Everolimus + letrozole<br>60 Palbociclib + letrozole<br>62 Bevacizumab + letrozole<br>61 Erlotinib + letrozole<br>100 Don't know
35	posttest_primary_mechanisms	Which of the following is a way primary resistance to endocrine	radio, Required<br>30 Estrogen receptor (ER)

		therapy in estrogen receptor-positive (ER+) breast cancer may occur?	| amplification<br>32 | RB1 amplification<br>31 | Cyclin D1 amplification<br>300 | None of the above<br>100 | Don't know
36	posttest_estrogen	Estrogen is a steroid hormone that controls cell processes like cell division, growth, differentiation, and proliferation.	radio, Required<br>1 | True<br>0 | False<br>100 | Don't know
37	posttest_primary_definition	Please fill in the blank with the correct answer. Primary resistance to endocrine therapy in ER+ breast cancer has been defined as disease progression less than ____ months after treatment in the metastatic setting.	radio, Required<br>8 | 8<br>6 | 6<br>12 | 12<br>10 | 10<br>100 | Don't know
38	posttest_rb1	Phosphorylation of RB1 facilitates which of the following:	radio<br>20 | Tumor suppression<br>22 | Entry into the S phase of the cell cycle<br>21 | Inhibition of cell cycle progression<br>23 | Formation of a cyclin D1-CDK4/6 complex<br>100 | Don't know
39	submit_posttest	Fourteen days after you click the submit survey button below you will receive an email with a link to a 5-minute follow-up survey. Upon completion, you will receive your $100 Amazon.com Gift Card.*<br><br>On the next screen, we will ask you for feedback on the learning materials.<br><br>*Amazon.com is not a sponsor of this promotion. Except as required by law, Amazon.com Gift Cards ("GCs") cannot be transferred for value or redeemed for cash. GCs may be	descriptive

		used only for purchases of eligible goods at Amazon.com or certain of its affiliated websites. For complete terms and conditions, see www.amazon.com/gc-legal. GCs are issued by ACI Gift Cards, Inc., a Washington corporation. All Amazon ®, ™ & © are IP of Amazon.com, Inc. or its affiliates. No expiration date or service fees.	
40	postlearning_material_review_survey_complete	Complete?	dropdown<br>0 Incomplete<br>1 Unverified<br>2 Complete
Instrument: **Feedback Survey**			
41	easy_understanding	The information I viewed was easy to understand.	radio<br>1 Strongly disagree<br>2 Disagree<br>3 Neither agree or disagree<br>4 Agree<br>5 Strongly agree
42	information_confusing	Some of the information I viewed was confusing.	radio<br>1 Strongly disagree<br>2 Disagree<br>3 Neither agree or disagree<br>4 Agree<br>5 Strongly agree
43	information_satisfaction	I am satisfied with the information I viewed.	radio<br>1 Strongly disagree<br>2 Disagree<br>3 Neither agree or disagree<br>4 Agree<br>5 Strongly agree
44	information_new	The information I viewed taught me something new.	radio<br>1 Strongly disagree<br>2 Disagree

			3 Neither agree or disagree<br>4 Agree<br>5 Strongly agree
45	information_openended	Do you have any other thoughts on this material that you would like to share with us?	notes
46	feedback_survey_complete	Complete?	dropdown<br>0 Incomplete<br>1 Unverified<br>2 Complete
Instrument: **Knowledge Follow-up Test**			
47	followup_fda_approved	Which drug combination is FDA-approved to treat ER+ metastatic breast cancer resistant to letrozole or anastrazole?	radio, Required<br>51 Erlotinib + exemestane<br>52 Palbociclib + exemestane<br>53 Bevacizumab + exemestane<br>50 Everolimus + exemestane<br>100 Don't know
48	followup_rb1	Phosphorylation of RB1 facilitates which of the following:	radio, Required<br>22 Entry into the S phase of the cell cycle<br>21 Inhibition of cell cycle progression<br>23 Formation of a Cyclin D1-CDK4/6 complex<br>20 Tumor suppression<br>100 Don't know
49	followup_palbociclib	Palbociclib is in which of the following drug classes?	radio, Required<br>71 CDK4/6 inhibitors<br>73 EGFR inhibitors<br>70 mTOR inhibitors<br>72 PI3K inhibitors<br>100 Don't know
50	followup_acquired_mechanisms	Which of the following are ways acquired resistance to endocrine therapy in ER+ breast may occur?	radio, Required<br>41 Changes in the tumor microenvironment<br>42 Estrogen receptor mutations

			40 Activation of growth factor signaling pathways<br>200 All of the above<br>100 Don't know
51	followup_estrogen	Estrogen is a steroid hormone that controls cell processes like cell division, growth, differentiation, and proliferation.	radio, Required<br>1 True<br>0 False<br>100 Don't know
52	followup_fda_accelerated	The FDA granted accelerated approval of which of the following drug combinations for first line or initial endocrine therapy in postmenopausal women with ER+/HER2-negative advanced breast cancer?	radio, Required<br>63 Everolimus + letrozole<br>62 Bevacizumab + letrozole<br>61 Erlotinib + letrozole<br>60 Palbociclib + letrozole<br>100 Don't know
53	followup_primary_definition	Please fill in the blank with the correct answer. Primary resistance to endocrine therapy in ER+ breast cancer has been defined as disease progression less than ____ months after treatment in the metastatic setting.	radio, Required<br>10 10<br>8 8<br>6 6<br>12 12<br>100 Don't know
54	followup_primary_mechanisms	Which of the following is a way primary resistance to endocrine therapy in estrogen receptor-positive (ER+) breast cancer may occur?	radio, Required<br>32 RB1 amplification<br>30 Estrogen receptor (ER) amplification<br>31 Cyclin D1 amplification<br>300 None of the above<br>100 Don't know
55	followup_cdk46_settings	CDK4/6 inhibitors have only been evaluated in the first line setting in breast cancer.	radio, Required<br>1 True<br>0 False<br>100 Don't know
56	followup_acquired_definition	Acquired resistance to endocrine therapy in ER+ breast cancer is defined as recurrence at least 6-12 months after completion of adjuvant therapy or disease progression > 6 months after	radio, Required<br>1 True<br>0 False<br>100 Don't know

		endocrine therapy initiated in the metastatic setting.	
57	followup_survey_complete	Complete?	dropdown<br>0 Incomplete<br>1 Unverified<br>2 Complete
Instrument: **Demographics Survey**			
58	practice_type	What is your practice type?	radio<br>1 Academic<br>3 Community<br>4 Both<br>5 Other
59	other_practicetype<br>Show the field ONLY if:<br>[practice_type] = '5'	Other	text
60	percentage_breastpatients	What is the percentage of patients seen with breast cancer in your practice?	radio<br>1 Less than 25%<br>2 25%-50%<br>3 Greater than 50%<br>4 Unknown
61	age_years	What is your age (years)?	text (integer, Min: 18, Max: 120)
62	gender	What is your gender?	radio<br>1 Male<br>2 Female<br>3 Prefer not to say
63	ethnicity	Ethnicity:	radio<br>1 Hispanic or Latino<br>2 Non-Hispanic<br>3 Prefer not to say
64	race	Race:<br>*Check all that apply.*	checkbox<br>1 ethnicity___1 White<br>3 ethnicity___3 Black or African American<br>4 ethnicity___4 Native Hawaiian or Other Pacific

			| | | Islander | | 5 | ethnicity___5 | Asian | | 6 | ethnicity___6 | American Indian or Alaska Native | | 97 | ethnicity___97 | Other | | 99 | ethnicity___99 | Unknown | | 98 | ethnicity___98 | Prefer not to say |
65	demographics_survey_complete	Complete?	dropdown<br>0 Incomplete<br>1 Unverified<br>2 Complete
Instrument: **Amazon Gift Card Survey**			
66	send_my_gift_card	I want to receive the $100 Amazon Gift Card.	yesno, Required<br>1 Yes<br>0 No
67	vanderbilt_employee<br>Show the field ONLY if:<br>[send_my_gift_card] = '1'	Are you a Vanderbilt University or Vanderbilt University Medical Center employee (faculty, staff, or other)?	yesno, Required<br>1 Yes<br>0 No
68	ssn<br>Show the field ONLY if:<br>[vanderbilt_employee] = '1'	Please provide your social security number.	text, Required, Identifier
69	vu_finance<br>Show the field ONLY if:<br>[send_my_gift_card] = '1'	The Vanderbilt University Finance Department requires us to collect your name and address to issue your gift card. Your information will not be used for anything other than to send the gift card, and it will not be used after completion of the study.	descriptive
70	first_name<br>Show the field ONLY if:<br>[send_my_gift_card] = '1'	First Name	text, Required, Identifier
71	middle_initial<br>Show the field ONLY if:<br>[send_my_gift_card] = '1'	Middle Initial	text, Identifier
72	last_name<br>Show the field ONLY if:<br>[send_my_gift_card] = '1'	Last Name	text, Required, Identifier
73	address_1	Address Line 1	text, Required, Identifier

<table>
<tr><td></td><td>Show the field ONLY if:
[send_my_gift_card] = '1'</td><td></td><td></td></tr>
<tr><td>74</td><td>address_2
Show the field ONLY if:
[send_my_gift_card] = '1'</td><td>Address Line 2</td><td>text, Identifier</td></tr>
<tr><td>75</td><td>city
Show the field ONLY if:
[send_my_gift_card] = '1'</td><td>City</td><td>text, Required, Identifier</td></tr>
<tr><td>76</td><td>state
Show the field ONLY if:
[send_my_gift_card] = '1'</td><td>State</td><td>dropdown, Required
<table>
<tr><td>1</td><td>Alabama</td></tr>
<tr><td>2</td><td>Alaska</td></tr>
<tr><td>3</td><td>American Samoa</td></tr>
<tr><td>4</td><td>Arizona</td></tr>
<tr><td>5</td><td>Arkansas</td></tr>
<tr><td>6</td><td>California</td></tr>
<tr><td>7</td><td>Colorado</td></tr>
<tr><td>8</td><td>Connecticut</td></tr>
<tr><td>9</td><td>Delaware</td></tr>
<tr><td>10</td><td>District of Columbia</td></tr>
<tr><td>11</td><td>Florida</td></tr>
<tr><td>12</td><td>Georgia</td></tr>
<tr><td>13</td><td>Guam</td></tr>
<tr><td>14</td><td>Hawaii</td></tr>
<tr><td>15</td><td>Idaho</td></tr>
<tr><td>16</td><td>Illinois</td></tr>
<tr><td>17</td><td>Indiana</td></tr>
<tr><td>18</td><td>Iowa</td></tr>
<tr><td>19</td><td>Kansas</td></tr>
<tr><td>20</td><td>Kentucky</td></tr>
<tr><td>21</td><td>Louisiana</td></tr>
<tr><td>22</td><td>Maine</td></tr>
<tr><td>23</td><td>Maryland</td></tr>
<tr><td>24</td><td>Massachusetts</td></tr>
<tr><td>25</td><td>Michigan</td></tr>
<tr><td>26</td><td>Minnesota</td></tr>
<tr><td>27</td><td>Mississippi</td></tr>
<tr><td>28</td><td>Missouri</td></tr>
</table></td></tr>
</table>

			29 Montana<br>30 Nebraska<br>31 Nevada<br>32 New Hampshire<br>33 New Jersey<br>34 New Mexico<br>35 New York<br>36 North Carolina<br>37 North Dakota<br>38 Northern Marianas Islands<br>39 Ohio<br>40 Oklahoma<br>41 Oregon<br>42 Pennsylvania<br>43 Puerto Rico<br>44 Rhode Island<br>45 South Carolina<br>46 South Dakota<br>47 Tennessee<br>48 Texas<br>49 Utah<br>50 Vermont<br>51 Virginia<br>52 Virgin Islands<br>53 Washington<br>54 West Virginia<br>55 Wisconsin<br>56 Wyoming
77	zip<br>Show the field ONLY if:<br>[send_my_gift_card] = '1'	ZIP Code	text, Identifier
78	md_nonphysician<br>Show the field ONLY if:<br>[send_my_gift_card] = '1' and ([enrollment_arm_1][role_description] = '2' or [enrollment_arm_1]	I have an M.D. or equivalent degree (e.g., D.O., M.B.B.S)	yesno, Required<br>1 Yes<br>0 No

	[role_description] = '3' or [enrollment_arm_1] [role_description] = '4' or [enrollment_arm_1] [role_description] = '5' or [enrollment_arm_1] [role_description] = '6')		
79	country<br>Show the field ONLY if:<br>[send_my_gift_card] = '1' and ([enrollment_arm_1] [role_description] = '1' or [md_nonphysician] = '1')	Are you legally authorized to practice medicine in the United States?	yesno, Required<br>1 Yes<br>0 No
80	sunshine_act<br>Show the field ONLY if:<br>[country] = '1' AND [send_my_gift_card] = '1' AND ([enrollment_arm_1] [role_description] = '1' or [md_nonphysician] = '1')	Because this study is funded by Pfizer, we are required to collect information from physicians to fulfill the Sunshine Act reporting requirements to send out the Amazon gift cards. Please fill out the information below to receive your card. Your information will not be used for anything other than to send the gift card, and it will not be used after completion of the study.	descriptive
81	credential<br>Show the field ONLY if:<br>[country] = '1' and ([enrollment_arm_1] [role_description] = '1' or [md_nonphysician] = '1')	Credential	radio, Required<br>1 M.D.<br>2 D.O.<br>3 M.B.B.S.<br>4 Other
82	other_credential<br>Show the field ONLY if:<br>[credential] = '4'	If you selected other, please explain:	text, Required
83	npi_id<br>Show the field ONLY if:<br>[country] = '1' and ([enrollment_arm_1] [role_description] = '1' or [md_nonphysician] = '1')	NPI ID<br>*Please note that we will not be able to send out the $100 Amazon gift card without your NPI ID. If you do not remember your NPI ID, please look it up using this link: NPI Registry Search*	text (integer), Required, Identifier
84	amazon_disclaimer_2	*Amazon.com is not a sponsor of this promotion. Except as required by law, Amazon.com Gift Cards ("GCs") cannot be transferred for value or redeemed for cash. GCs may be used only for purchases of eligible goods at Amazon.com	descriptive

		or certain of its affiliated websites. For complete terms and conditions, see www.amazon.com/gc-legal. GCs are issued by ACI Gift Cards, Inc., a Washington corporation. All Amazon ®, ™ & © are IP of Amazon.com, Inc. or its affiliates. No expiration date or service fees.	
85	amazon_gift_card_complete	Complete?	dropdown<br>0 Incomplete<br>1 Unverified<br>2 Complete
