# Supplementary material for: Internet-Based Assessment of Oncology Health Care Professional Learning Style and Optimization of Materials for Web-Based Learning: Controlled Trial With Concealed Allocation
Source: J Med Internet Res. 2017 Jul 25;19(7):e265. doi: 10.2196/jmir.7506 (PMC5548983; doi:10.2196/jmir.7506)
Supplement: Multimedia Appendix 5 [file jmir_v19i7e265_app5.pdf]

Multimedia Appendix 5

Participant Tracking and Related Analyses

Participant Tracking Methods

Google Analytics revealed information about participant interaction with the educational materials. For this study, unique site visitors were distinguished using cookies tracking the participant personal identification numbers (PINs) sent with the recruitment emails. Analytics recorded included time of day, pages visited, length of visit excluding the last page visited, technology used (i.e., desktop, mobile, or tablet), and others.

Results of Ancillary Analyses

Google Analytics were gathered on 627 unique participants; participants who were excluded from the outcomes analysis because they did not complete all three knowledge assessments were not excluded from analytics-based analyses. We determined that there was an average of 1.3 sessions per PIN and an average of 1.91 pages per session. Average session duration for sessions with measurements was 18 minutes 43 seconds. Only sessions where the participant clicked on a link within the website recorded durations; these durations are underestimates by definition, because the last page visited is not counted. Average session duration was 17 minutes 26 seconds, 27 minutes 9 seconds, and 19 minutes 59 seconds for desktop, mobile, and tablet technologies, respectively.

Figure 1 displays the central time zone stamp of participants viewing the control or intervention materials in a total of 845 distinct sessions. Participant time zone is not recorded by Google. The business addresses recorded for physician participants in the national provider index (NPI) database [1] reflect 42 U.S. states and the District of Columbia, so we expect that each participant accessed the study from at least one of at least four time zones. When grouped in two-hour blocks, usage peaked from 8:00-9:59 am and 6:00-7:59 pm central daylight time (CDT), despite the bulk of the emails being sent at noon (Figure 1). The participation nadir time period (n=8) was 2:00-3:59 am CDT.

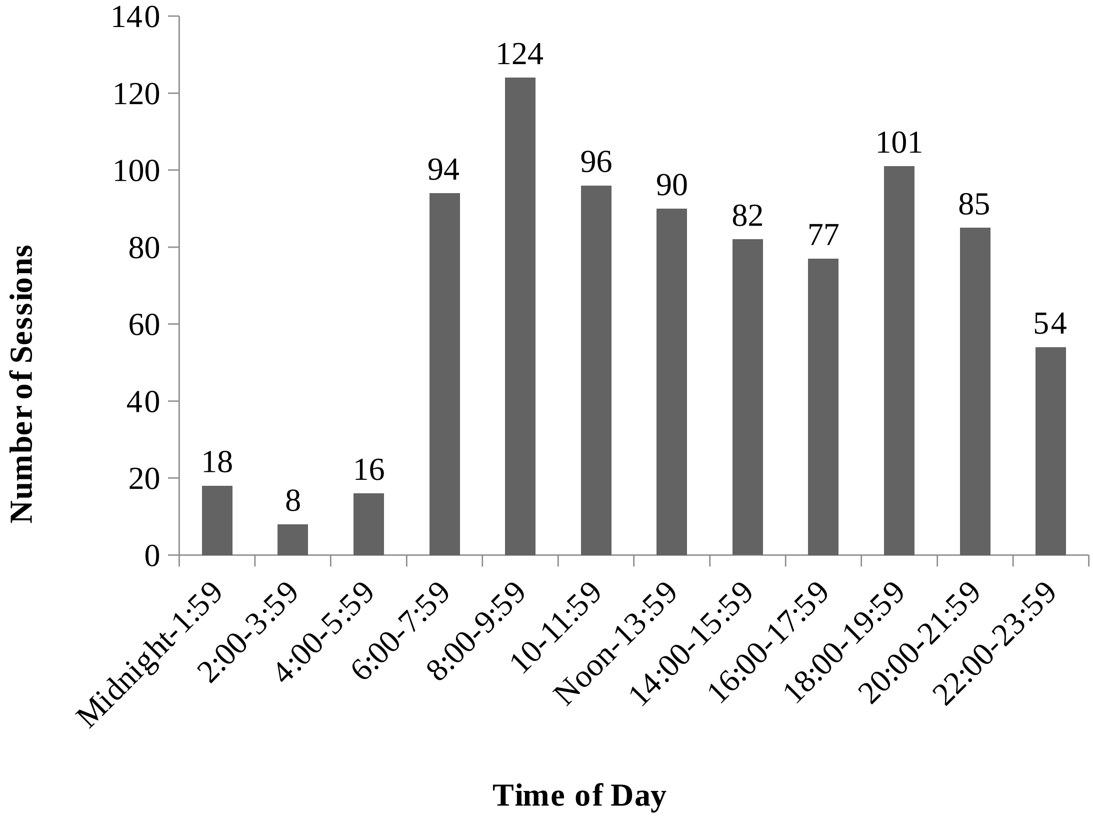

Figure 1. Time of day (recorded as CDT, regardless of participant time zone) of viewing of educational materials.

For the control materials, participants were unlikely to click hyperlinks leading to other pages with knowledge test answers, although 8 of 10 answers were available on the landing page, "ER (ESR1) in Breast Cancer" (Table 1).

Table 1. Number of pageviews of pages with knowledge test answers in control educational materials.

Page Title	Number of Pageviews	Number of Knowledge Test Answers on Page
ER (ESR1) in Breast Cancer	600	8 (questions 2-9)
ER (ESR1) Expression in Breast Cancer	23	2 (questions 6 and 8)
ER (ESR1)	11	1 (question 1)
CDK4/CDK6 Inhibition and CDK4/CDK6 Inhibitors in Breast Cancer	7	3 (questions 7, 8, and 10)
Palbociclib in Breast Cancer	5	1 (question 8)
MTOR Inhibition and MTOR Inhibitors in Breast Cancer	1	1 (question 6)
Everolimus in Breast Cancer	1	1 (question 6)

Discussion of Ancillary Analyses

In addition to the primary objectives of the study, we analyzed the time of day during which learning materials were accessed. However, interpretation of these analytics is difficult as participants could participate in the study from any domestic or international time zone. It is a reasonable assumption that most learning materials were accessed from one of the four continental US time zones. Despite emails being sent at noon CDT, participation peaked at 8:00-9:59 CDT and again at 18:00-17:59 CDT. Very few viewing instances occurred during what would be considered predominantly night hours in the US, with the participation nadir occurring at 2:00-3:59 CDT. A steady stream of viewing instances occurred during non-peak daytime hours. Based on hours of peak response, peak viewing of learning materials occurred in the early morning or in the later evening hours, and future efforts to engage medical providers via email contact should consider this timing.

Further, we analyzed the time it took our participants to complete the study. Our estimate (10-15 minutes) of the time it would take to participate in the study may have underestimated the time required. The time spent accessing the learning materials on a desktop or tablet was roughly equivalent, while the time spent by participants on mobile devices was higher. Because Google Analytics only records visit times for participants who clicked on a link during their session, the data oversampled control arm participants, since intervention arm participants do not need to visit more than one page to access all answers to the knowledge assessments. While our materials were presented on responsive websites, meaning they were adaptable to a wide variety of screen sizes, these time results indicate it may be beneficial to create abbreviated content for access on mobile devices.

Abbreviations

CDK4: cyclin-dependent kinase 4 protein
CDK6: cyclin-dependent kinase 6 protein
CDT: central daylight time
ER: estrogen receptor protein
ESR1: estrogen receptor 1 gene
MTOR: mechanistic target of rapamycin gene
NPI: National Provider Identifier
PIN: personal identification number
US: United States

References

1. National Plan and Provider Enumeration System, Centers for Medicare and Medicaid Services, U.S. Department of Health and Human Services. 2016. NPPES NPI Registry. https://npiregistry.cms.hhs.gov/. Archived at: http://www.webcitation.org/6kECvzXa5
