# Supplementary material for: Internet-Based Assessment of Oncology Health Care Professional Learning Style and Optimization of Materials for Web-Based Learning: Controlled Trial With Concealed Allocation
Source: J Med Internet Res. 2017 Jul 25;19(7):e265. doi: 10.2196/jmir.7506 (PMC5548983; doi:10.2196/jmir.7506)
Supplement: Multimedia Appendix 6 [file jmir_v19i7e265_app6.pdf]

Multimedia Appendix 6

Educational Materials

Table of Contents

Intervention Educational Materials 1
Audio Script (version 1) 1
Text (version 1) 5
Slides (version 1) 17
Audio Script (version 2) 68
Text (version 2) 73
Control Educational Materials 77

Intervention Educational Materials

Audio Script (version 1)

Overcoming Resistance to Endocrine Therapy in ER-positive Breast Cancer: CDK4/6 Inhibition as a Novel Treatment Strategy

This is a Presentation Prepared by My Cancer Genome at Vanderbilt University Medical Center in the summer of 2015 with funding from Pfizer Independent Grants for Learning and Change

We will begin the presentation with an Introduction to Estrogen Receptor Signaling and Endocrine Therapy in ER-positive Breast Cancer

Estrogen is a steroid hormone that controls cellular processes such as cell division, growth, differentiation, and proliferation in tissues such as breast and ovaries. Estrogen is converted from androgen precursors by the aromatase enzyme. Estrogen acts as a ligand and binds to the estrogen receptor, known as *ER*, which results in changes in gene expression and the activation of signaling pathways that regulate cell growth processes, such as the cell cycle.

Estrogen signaling can drive cell growth in several types of cancer, including breast, ovarian, endometrial, and prostate cancer. Seventy percent of breast cancers express the estrogen receptor, which is referred to as estrogen receptor positive, or ER-positive breast cancer.

There are three classes of endocrine agents, which are used to treat ER-positive breast cancer:

Steroidal and nonsteroidal aromatase inhibitors, such as anastrozole, letrozole, and exemestane, inhibit the enzyme aromatase, which prevents the synthesis of estrogen. These agents are used in postmenopausal women in adjuvant and metastatic settings.

Selective estrogen receptor modulators, or SERMs, such as tamoxifen, compete with estrogen as a ligand for the estrogen receptor, inhibiting the pathway. These agents are used in premenopausal and postmenopausal women in adjuvant and metastatic settings.

Selective estrogen receptor modulators, or SERMs, such as tamoxifen, compete with estrogen as a ligand for the estrogen receptor, inhibiting the pathway. These agents are used in premenopausal and postmenopausal women in adjuvant and metastatic settings.

Selective estrogen receptor degraders or downregulators, or SERDs, such as fulvestrant, bind to the estrogen receptor and cause it to change shape, which inhibits the receptor's ability to bind estrogen and sometimes downregulates the estrogen receptor itself. These agents are used in postmenopausal women in the metastatic setting.

We will now review Resistance to Endocrine Agents

Despite the presence of estrogen receptor expression in breast cancer tissues, not all ER-positive breast cancers respond to endocrine therapy. Molecular alterations in ER-positive breast cancers can lead to primary or acquired resistance to endocrine therapy.

Primary resistance is defined as recurrence, either within adjuvant therapy or within 6 to 12 months of completion of adjuvant therapy or as disease progression less than 6 months after treatment.

Primary resistance may occur through changes that affect the estrogen receptor, gene expression, or the cell cycle and include the following:

- FGFR amplifications,
- Loss of Erα,
- Post-translational modification of Erα,
- ER mutations,
- Expression of ER coactivation/corepression factors,
- MYC amplification and overexpression, or
- Cyclin D1 amplification or expression.

Acquired resistance is defined as recurrence at least 6 to 12 months after completion of adjuvant therapy or as disease progression more than 6 months after endocrine therapy was initiated in the metastatic setting.

Acquired resistance to endocrine therapy can occur in several ways, including:

- Activation of growth factor signaling pathways
- PI3K/AKT/MTOR or
- MAPK/ERK,
- ER mutations, or
- Changes in the tumor microenvironment.

We will now discuss several Approaches to Addressing Resistance to Endocrine Therapy

In recent years, there has been an increase in the number of clinical trials using targeted therapeutic strategies in endocrine resistant ER-positive breast cancer, with varying levels of evidence for improvement in outcomes:

- Everolimus is an MTOR inhibitor that is FDA approved for use in combination with exemestane in second line therapy;

- Palbociclib is a CDK4/6 inhibitor that is FDA approved for use in combination with letrozole; there is also strong evidence for use of palbociclib in combination with fulvestrant. Evidence is promising for additional CDK4/6 inhibitors ribociclib and abemaciclib;
- Entinostat in an HDAC inhibitor for which evidence is promising;
- Results of clinical trials of EGFR/HER2 inhibitors have thus far been mixed;
- Efficacy of IGF-1R, FGFR, and PI3K/AKT inhibitors is not known at this time; and
- Finally, evidence does not support efficacy of antiangiogenesis inhibitors.

In the next sections, we will first summarize the use of MTOR inhibitors and then focus on the use of CDK4/6 inhibitors to overcome resistance to endocrine agents.

We will now discuss the Use of MTOR Inhibitors to Overcome Resistance to Endocrine Agents

As shown earlier, one mechanism of resistance to endocrine agents is activation of the PI3K/AKT/mTOR signaling pathway. In patients whose tumors have demonstrated resistance to nonsteroidal aromatase inhibitors, dual inhibition of the PI3K/AKT1/MTOR signaling pathway and the estrogen signaling pathway provides additional improvement in efficacy in clinical studies. Everolimus is a kinase inhibitor targeting MTOR approved in combination with exemestane for the treatment of postmenopausal women with HER2-negative hormone receptor positive breast cancer after failure of treatment with letrozole or anastrozole, based on results from the BOLERO-2 trial.

We will now discuss the Use of Cyclin-Dependent Kinase, also known as CDK4/6, Inhibitors to Overcome Resistance to Endocrine Agents

Use of CDK4/6 inhibitors is being explored as a strategy to overcome resistance to endocrine agents.

ER regulates expression of cyclin D1. Cyclin D1 is involved in regulating entry into the synthesis phase, or S phase, of the cell cycle. It binds to cyclin-dependent kinases 4 and 6, which are collectively referred to as CDK4/6. The cyclin D1–CDK4/6 complex phosphorylates the retinoblastoma tumor suppressor protein, also called RB1. RB1 releases the transcription factors required for entry into the S phase of the cell cycle.

Secondary signals in the estrogen signaling pathway can become activated through cyclin D1 overexpression or amplification, CDK4/6 gain of function, and RB1 alterations causing loss of function. This activation can cause resistance to endocrine agents.

Combining ER-targeting agents, such as aromatase inhibitors, with agents that target downstream components of the estrogen receptor pathway, such as CDK 4/6 inhibitors, may improve therapeutic benefit compared to standard of care. Inhibition of CDK4/6 is being investigated in patients with hormone receptor positive breast cancer in the adjuvant and neoadjuvant settings, as first-line therapy, and after resistance develops to endocrine therapy. Note that in the subset of patients whose tumors demonstrate RB1 mutation or loss, CDK4/6 inhibitors may not be effective, because the RB1 alteration occurs downstream of that signaling node.

Several cell cycle inhibitors have been developed which target CDK4/6. The CDK4/6 inhibitors include:

1. Palbociclib: Phase 3 studies include PALOMA-2; PALOMA-3; PEARL; and PENELOPE-B;
2. Ribociclib: the Phase 3 study is MONALEESA-2; and
3. Abemaciclib: Phase 3 studies include MONARCH 2 and MONARCH 3.

We will summarize the current status of clinical trials for palbociclib, ribociclib, and abemaciclib.

Palbociclib is the first CDK4/6 inhibitor we will discuss; results of several clinical trials have been reported. One phase one and two phase two trials of palbociclib, either alone or in combination with letrozole, showed clinical benefit. In the PALOMA-1 randomized Phase 2 trial, patients with metastatic ER-positive/HER2-negative breast cancer treated with first-line palbociclib in combination with the aromatase inhibitor letrozole demonstrated an improvement in median progression-free survival compared with patients treated with letrozole alone. Based on the outcomes from this trial, the combination of palbociclib and letrozole received accelerated FDA approval for ER-positive/HER2-negative first-line metastatic breast cancer. The Phase 3 trial of this combination is still ongoing.

On April 15, 2015, the PALOMA–3 phase three trial of palbociclib plus fulvestrant was stopped after the interim analysis showed efficacy in metastatic estrogen-receptor positive, HER2 negative breast cancer "following disease progression during or after endocrine therapy".

There are several ongoing phase one, two, and three clinical trials evaluating palbociclib in combination with other endocrine therapies as well as anti-HER2 therapies. These trials cover the neoadjuvant and adjuvant setting as well as the first, second, and third line therapies in the metastatic setting.

Ribociclib is the second CDK4/6 inhibitor we will discuss; results of several phase 1 clinical trials have been reported. In preliminary results from a phase Ib study, 6 patients with ER-positive, HER2-negative, locally advanced or metastatic breast cancer were treated with the CDK4/6 inhibitor ribociclib, also known as LEE011, in combination with letrozole with one dose-limiting toxicity (neutropenia) reported. A 67% clinical benefit rate was reported in 6 patients.

Ribociclib is being studied in the MONALEESA-2 Phase 3 trial in combination with letrozole in first-line ER-positive metastatic breast cancer. Patients must not have experienced progression within 12 months of completing adjuvant letrozole or anastrozole. A phase 2 trial, called MONALEESA-1, is closed to accrual, but the data are not yet reported. There are several ongoing phase one, two, and three clinical trials evaluating ribociclib in combination with other endocrine therapies as well as anti-HER2 therapies. These trials cover first, second, and greater line therapies in the metastatic setting.

Abemaciclib is the third CDK4/6 inhibitor we will discuss; it is under evaluation for use in estrogen-receptor-positive metastatic breast cancer In a Phase 1 trial with several expansion cohorts, the CDK4/6 inhibitor abemaciclib showed activity in several tumor types, including lung cancer and breast cancer, in preliminary reports. In an ER-positive metastatic breast cancer cohort, 36 patients treated with the combination of abemaciclib and fulvestrant demonstrated a clinical benefit rate of 81%.

Abemaciclib is being evaluated in two Phase 3 clinical trials in combination with endocrine therapies: MONARCH 2 and MONARCH 3. These trials cover the first, second, and greater line therapies in the metastatic setting.

In Summary,

CDK4/6 inhibitors are a promising therapy for ER-positive metastatic breast cancer. The FDA has granted accelerated approval to palbociclib for use in combination with letrozole for first-line or initial treatment in postmenopausal women with ER-positive/HER2-negative advanced breast cancer. Ongoing trials are evaluating safety and efficacy of CDK4/6 inhibitors alone and in combination with other therapies.

Text (version 1)

Overcoming Resistance to Endocrine Therapy in ER+ Breast Cancer: CDK 4/6 Inhibition as a Novel Treatment Strategy
Prepared by My Cancer Genome at Vanderbilt University Medical Center with funding from Pfizer Independent Grants for Learning & Change
Summer 2015

Introduction to Estrogen Receptor Signaling and Endocrine Therapy in ER+ Breast Cancer

Estrogen is a steroid hormone that controls cellular processes such as cell division, growth, differentiation, and proliferation in tissues such as breast and ovaries. Estrogen is converted from androgen precursors by the aromatase enzyme. Estrogen acts as a ligand and binds to the estrogen receptor (*ESR1*, commonly known as *ER*), which results in changes in gene expression and the activation of signaling pathways that regulate cell growth processes, such as the cell cycle.

Estrogen signaling can drive cell growth in several types of cancer, including breast, ovarian, endometrial, and prostate cancer. Seventy percent of breast cancers express the estrogen receptor, which is referred to as estrogen receptor positive (ER+) breast cancer (Allred et al. 2009; Colditz et al. 2004).

There are three classes of endocrine agents, which are used to treat ER+ breast cancer:

- Steroidal and nonsteroidal aromatase inhibitors, such as anastrozole, letrozole, and exemestane, inhibit the enzyme aromatase, which prevents the synthesis of estrogen. These agents are used in postmenopausal women in adjuvant and metastatic settings.
- Selective estrogen receptor modulators, or SERMs, such as tamoxifen, compete with estrogen as a ligand for the estrogen receptor, inhibiting the pathway. These agents are used in premenopausal and postmenopausal women in adjuvant and metastatic settings.
- Selective estrogen receptor degraders or downregulators, or SERDs, such as fulvestrant, bind to the estrogen receptor and cause it to change shape, which inhibits the receptor's ability to bind estrogen and sometimes downregulates the estrogen receptor itself. These agents are used in postmenopausal women in the metastatic setting.

Resistance to Endocrine Agents

Despite the presence of estrogen receptor expression in breast cancer tissues, not all ER+ breast cancers respond to endocrine therapy. Molecular alterations in ER+ breast cancers can lead to primary or acquired resistance to endocrine therapy.

Primary resistance is defined as recurrence, either within adjuvant therapy or within 6 to 12 months of completion of adjuvant therapy or as disease progression less than 6 months after treatment (Bachelot et al. 2012).

Primary resistance may occur through changes that affect the estrogen receptor, gene expression, or the cell cycle (Bachelot et al. 2012; Osborne and Schiff 2011) and include the following:

- FGFR amplifications

- Loss of ERα
- Post-translational modification of ERα
- ER mutations
- Expression of ER coactivation/corepression factors
- MYC amplification and overexpression
- Cyclin D1 amplification or expression

Acquired resistance is defined as recurrence at least 6 to 12 months after completion of adjuvant therapy or as disease progression more than 6 months after endocrine therapy was initiated in the metastatic setting (Bachelot et al. 2012).

Acquired resistance to endocrine therapy can occur in several ways, including activation of growth factor signaling pathways or changes in the tumor microenvironment (Bachelot et al. 2012; Osborne and Schiff 2011).

Some ways acquired resistance may occur include:

- Activation of growth factor signaling pathways
  - PI3K/AKT/MTOR
  - MAPK/ERK
- ER mutations
- Changes in the tumor microenvironment

Approaches to Addressing Resistance to Endocrine Therapy

In recent years, there has been an increase in the number of clinical trials using targeted therapeutic strategies in endocrine resistant ER+ breast cancer, with varying levels of evidence for improvement in outcomes (Table 1). In the next sections, we will first summarize the use of MTOR inhibitors and then focus on the use of CDK 4/6 inhibitors to overcome resistance to endocrine agents.

Table 1. Approaches to Addressing Resistance to Endocrine Therapy.

Drug Class	Strength of Clinical Data	Evidence	Clinical Trials
MTOR Inhibitors	1 positive phase 3 in second line<br>1 negative phase 3 in first line	Strong evidence for everolimus	Everolimus (Afinitor) approved (+ exemestane)<br>~53 trials with everolimus
CDK 4/6 Inhibitors	1 positive phase 2 RCT in first line<br>1 positive phase 3 RCT in second line	Strong evidence for palbociclib / Promising for ribociclib and abemaciclib	Palbociclib (Ibrance) approved (+ letrozole)<br>Palbociclib + Fulvestrant trial stopped at interim for efficacy

			Palbociclib, ribociclib, and abemaciclib in phase 3 trials
HDAC Inhibitors	1 positive phase 2 in second line	Promising	Entinostat phase 3 trial initiated
EGFR/HER2 Inhibitors	1 negative phase 2 RCT in second line (gefitinib)<br>1 promising phase 2 (erlotinib 1st-line)	Mixed	Erlotinib phase 2 trial ongoing (+ fulvestrant)
IGF-1R Inhibitors	1 negative phase 2 in second line (agent: OSI-906)	Not known	Cixutumumab (IMC-A12) phase 1/2 trial ongoing
FGFR Inhibitors	N/A	Not known	Dovitinib and nintedanib phase 2 trials ongoing
PI3K/AKT Inhibitors	N/A	Not known	Buparlisib (BKM120) phase 3 trial ongoing
Angiogenesis Inhibitors	1 negative phase 2 RCT	Poor	Bevacizumab in phase 3 trials; motesanib phase 2 trials planned

NOTE: RCT = randomized clinical trial.

Using MTOR Inhibitors to Overcome Resistance to Endocrine Agents

As shown earlier, one mechanism of resistance to endocrine agents is activation of the PI3K/AKT/MTOR signaling pathway (Miller et al. 2010). In patients whose tumors have demonstrated resistance to nonsteroidal aromatase inhibitors, dual inhibition of the PI3K/AKT/MTOR signaling pathway and the estrogen signaling pathway provides additional improvement in efficacy in clinical studies (Baselga et al. 2012; Piccart et al. 2014; Yardley et al. 2013). Everolimus is a kinase inhibitor targeting MTOR approved in combination with exemestane for the treatment of postmenopausal women with HER2-negative hormone receptor positive breast cancer after failure of treatment with letrozole or anastrozole, based on results from the BOLERO-2 trial (FDA 2012; Piccart et al. 2014).

Using Cyclin-Dependent Kinase (CDK 4/6) Inhibitors to Overcome Resistance to Endocrine Agents

Use of CDK 4/6 inhibitors is being explored as a strategy to overcome resistance to endocrine agents.

ER regulates expression of cyclin D1. Cyclin D1 is involved in regulating entry into the synthesis phase, or S phase, of the cell cycle. It binds to cyclin-dependent kinases 4 and 6, which are collectively referred to as CDK 4/6. The cyclin D1–CDK 4/6 complex phosphorylates the retinoblastoma tumor suppressor protein, also called RB1. RB1 releases the transcription factors required for entry into the S phase of the cell cycle.

Secondary signals in the estrogen signaling pathway can become activated through cyclin D1 overexpression or amplification, CDK 4/6 gain of function, and RB1 alterations causing loss of function. This activation can cause resistance to endocrine agents.

Combining ER-targeting agents, such as aromatase inhibitors, with agents that target downstream components of the estrogen receptor pathway, such as CDK 4/6 inhibitors, may improve therapeutic benefit compared to standard of care. Inhibition of CDK 4/6 is being investigated in patients with hormone receptor positive breast cancer in the adjuvant and neoadjuvant settings, as first-line therapy, and after resistance develops to endocrine therapy. Note that in the subset of patients whose tumors demonstrate RB1 mutation or loss, CDK 4/6 inhibitors may not be effective, because the RB1 alteration occurs downstream of that signaling node.

Several cell cycle inhibitors have been developed which target CDK 4/6. The CDK 4/6 inhibitors include:

- Palbociclib (phase 3 studies: PALOMA-2; PALOMA-3; PEARL; PENELOPE-B)
- Ribociclib (phase 3 study: MONALEESA-2)
- Abemaciclib (phase 3 studies: MONARCH 2; MONARCH 3)

Reported results of clinical trials evaluating these CDK 4/6 inhibitors are summarized below.

Palbociclib

In a randomized phase 2 trial, patients with metastatic ER+/HER2-negative breast cancer treated with first-line palbociclib in combination with the aromatase inhibitor letrozole demonstrated an improvement in median progression-free survival compared with patients treated with letrozole alone (Table 2; Finn et al. 2015; PALOMA-1). Based on the outcomes from this trial, the combination of palbociclib and letrozole received accelerated FDA approval for ER+/HER2-negative first-line metastatic breast cancer (FDA 2015). The phase 3 trial of this combination is still ongoing. A phase 3 trial evaluating palbociclib in combination with fulvestrant in metastatic ER positive, HER2 negative breast cancer following disease progression during or after endocrine therapy showed improved progression-free survival relative to patients treated with placebo plus fulvestrant (PALOMA3; Turner et al. 2015). The trial was stopped early on April 15, 2015, due to efficacy.

There are several ongoing phase 1, 2, and 3 clinical trials evaluating palbociclib in combination with other endocrine therapies as well as anti-HER2 therapies (Table 3). These trials cover the neoadjuvant and adjuvant setting as well as the first, second, and third line therapies in the metastatic setting.

Table 2. Reported Trials with Palbociclib in Breast Cancer.

Reference	Study Type	Therapeutic setting	Treatment Agent	Mutation Status / Group	# Pts in Study	RR	PFS (months)	OS (months)

	**/ Phase**							
Turner et al. 2015 (PALOMA-3)	Phase 3	≥1st (no limit to prior therapies) metastatic or advanced breast cancer	palbociclib + fulvestrant	HR+	347	10.4%	9.2	
			placebo + fulvestrant		174	6.3%	3.8	
Finn et al. 2015 (PALOMA-1; TRIO-18)	Phase 2	1st line metastatic breast cancer	letrozole	ER+ / HER2– (cohort 1+2)	81	27%	10.2	33.3
				ER+ / HER2– (cohort 1)	32		5.7	
				ER+ / HER2– / CCND1+ or p16+ (cohort 2)	49		11.1	
			letrozole + palbociclib	ER+ / HER2– (cohort 1+2)	84	43%	20.2	37.5
				ER+ / HER2– (cohort 1)	34		26.1	
				ER+ / HER2– / CCND1+ or p16+ (cohort 2)	50		18.1	
			palbociclib	RB1+	37		3.7	

DeMichele et al. 2014	Phase 2	≥1st (no limit to prior therapies) metastatic breast cancer		HR+ / RB1+ subset	33		5.1	
		≥2 prior lines of hormone therapy		HR+ / RB1+ subset	24		5.0	
Clark et al. 2014	Phase 1	≥3rd line metastatic breast cancer	palbociclib + paclitaxel	RB1+	15	40%		

NOTE: CR = complete response; ER = estrogen receptor; OS = overall survival; PFS = progression-free survival; PR = partial response; Pts = patients; RR = response rate (CR + PR); RB1 = retinoblastoma.

Table 3. Ongoing and Recruiting Clinical Investigation with Palbociclib in Breast Cancer.

Study Type / Phase / ID	Therapeutic setting	Prior therapy requirement	Treatment Agent	Mutation Status / Group	# Pts in Study	Study Start Date
Phase 3 (PEARL, NCT02028507)	Any line, locally advanced or metastatic breast cancer	Recurrence during or within 12 months of adjuvant letrozole or anastrozole or during or within 1 month of letrozole or anastrozole for metastatic disease	palbociclib + exemestane	ER+ and/or PR+ HER2–	348	March 2014
			capecitabine			
Phase 3 (PENELOPE-B, NCT01864746)	Early breast cancer at high risk of relapse after showing less than pathological complete response to neoadjuvant taxane-	Prior neoadjuvant chemotherapy including taxane of at least 16 weeks	palbociclib + standard anti-hormonal therapy	ER+ and/or PR+ HER2–	800	November 2013
			Placebo + standard anti-			

	containing chemotherapy		hormonal therapy			
Phase 3 (PALOMA-2, NCT01740427)	1st line	No prior systemic anti-cancer therapy for advanced ER+ disease	palbociclib + letrozole	ER+ HER2–	650	February 2013
			placebo + letrozole			
Phase 2 (NCT02040857)	Adjuvant setting, breast cancer	One month of adjuvant tamoxifen or aromatase inhibitor; at least two more years of adjuvant therapy planned	palbociclib + tamoxifen or letrozole or anastrozole or exemestane	ER+ and/or PR+ HER2–	120	January 2014
Phase 2 (NCT01723774)	Neoadjuvant setting		palbociclib + anastrozole or anastrozole + goserelin	ER+ and/or PR+ HER2– PIK3CA mutation cohort	29	June 2013
Phase 1b (NCT01976169)	2nd line or greater, recurrent or metastatic breast cancer	Prior trastuzumab or HER2 targeted therapies required	palbociclib + trastuzumab-DM1	HER2+ RB1 proficient	17	January 2014

NOTE: ER = estrogen receptor; HER2 = human epidermal growth factor receptor 2; PR = progesterone receptor; Pts = patients; RB1 = retinoblastoma.

Ribociclib

In preliminary results from a phase Ib study, 6 patients with ER+, HER2-negative locally advanced or metastatic breast cancer were treated with the CDK 4/6 inhibitor ribociclib (also known as LEE011) in combination with letrozole with one dose-limiting toxicity (neutropenia) reported (Table 4). A 67% clinical benefit rate was reported in 6 patients (Munster et al. 2014).

Ribociclib is in a Phase 3 trial (MONALEESA-2) in combination with letrozole in first-line ER+ metastatic breast cancer (Table 5). Patients must not have experienced progression within 12 months of completing adjuvant letrozole or anastrozole.

Table 4. Reported Trials with Ribociclib in Breast Cancer.

Reference	Study Type / Phase	Therapeutic setting	Treatment Agent	Mutation Status / Group	# Pts in Study	RR	PFS (months)	OS (months)
Munster et al. 2014 (NCT01872260)	Phase 1b	1st line metastatic breast cancer	ribociclib + letrozole	ER+ / HER2–	10	67% (CBR)		

NOTE: CBR = clinical benefit rate (CR + PR + SD); CR = complete response; ER = estrogen receptor; OS = overall survival; PFS = progression-free survival; PR = partial response; Pts = patients; RR = response rate (CR + PR); SD = stable disease.

Table 5. Ongoing Clinical Investigation with Ribociclib in Breast Cancer.

Study Type / Phase / ID	Therapeutic setting	Prior Therapy Requirement	Treatment Agent	Mutation Status / Group	# Pts in Study	Study Start Date
Phase 3 (MONA LEESA-2, NCT01958021)	1st line, locally advanced or metastatic breast cancer, postmenopausal	No progression within 12 months of completing adjuvant letrozole or anastrole	ribociclib + letrozole<br>placebo + letrozole	ER+ and/or PR+ HER2–	500	December 2013
Phase 2 (SIGNATURE, NCT02187783)	2nd line or greater, any cancer except non-triple negative breast cancer		ribociclib	CDK 4/6, cyclin D1/3, or p16 aberrations	90	August 2014
Phase 2 (MONA LEESA-1, NCT01919229)	1st line, locally advanced or metastatic breast cancer, postmenopausal		ribociclib 400 mg + letrozole<br>ribociclib 600 mg + letrozole<br>letrozole	ER+ and/or PR+ HER2–	120	October 2013

Phase 1b/2 (NCT01872260)	1st line, locally advanced or metastatic breast cancer, postmenopausal (phase II, phase Ib dose expansions); Any line (phase Ib dose escalation)	No progression within 12 months of completing adjuvant letrozole	ribociclib + letrozole<br>BYL719 + letrozole<br>ribociclib + BYL719 + letrozole	ER+ HER2–	300	October 2013
Phase 1b/2 (NCT01857193)	1st or greater locally advanced or metastatic breast cancer, postmenopausal	Recurrence during or within 12 months of adjuvant letrozole or anastrozole; or during or within 1 month of letrozole or anastrozole for metastatic disease	ribociclib + everolimus + exemestane<br>ribociclib + exemestane<br>everolimus + exemestane	ER+ HER2–	185	September 2013
Phase 1b/2 (NCT02088684)	Any line, locally advanced or metastatic breast cancer, postmenopausal		ribociclib + BKM120 + fulvestrant<br>ribociclib + BYL719 + fulvestrant<br>ribociclib + fulvestrant	ER+ HER2–	216	May 2014
Phase 1 (NCT02154776)	1st line, locally advanced or metastatic breast cancer; 2nd line or greater in the		ribociclib + buparlisib + letrozole	ER+ HER2–	50	June 2014

	dose escalation phase					

NOTE: ER = estrogen receptor; HER2 = human epidermal growth factor receptor 2; PR = progesterone receptor; Pts = patients.

Abemaciclib

In a phase 1 trial with several expansion cohorts, the CDK 4/6 inhibitor abemaciclib showed activity in several tumor types, including lung cancer and breast cancer, in preliminary reports (Table 6). In an ER+ metastatic breast cancer cohort, 36 patients treated with the combination of abemaciclib and fulvestrant demonstrated a clinical benefit rate of 81% (Patnaik et al. 2014a; Patnaik et al. 2014b; Goldman et al. 2014; Shapiro et al. 2013).

Abemaciclib is being evaluated in two phase 3 clinical trials in combination with endocrine therapies (Table 7; MONARCH 2; MONARCH 3). These trials cover the first, second, and greater line therapies in the metastatic setting.

Table 6. Reported Trial with Abemaciclib in Breast Cancer.

Reference	Study Type / Phase	Therapeutic setting	Treatment Agent	Mutation Status / Group	# Pts in Study	RR	PFS (months)	OS (months)
Patnaik et al. 2014a; Patnaik et al. 2014b	Phase 1	Metastatic breast cancer	abemaciclib	HR+	36	25%		
			abemaciclib + fulvestrant (expanded cohort)		13	62%		

NOTE: CR = complete response; ER = estrogen receptor; HR = hormone receptor (ER and/or PR); OS = overall survival; PFS = progression-free survival; PR = partial response; PR = progesterone receptor; Pts = patients; RR = response rate (CR + PR).

Table 7. Ongoing and Recruiting Clinical Investigation with Abemaciclib in Breast Cancer.

Study Type / Phase / ID	Therapeutic setting	Prior therapy requirement	Treatment Agent	Mutation Status / Group	# Pts in Study	Study Start Date
Phase 3 (MONARCH 3, NCT02246621)	1st line or greater, locally advanced or metastatic		abemaciclib + anastrozole or letrozole	ER+ and/or PR+ HER2–	450	October 2014, not yet recruiting

	breast cancer, postmenopausal		anastrozole or letrozole + placebo			
Phase 3 (MONARCH 2, NCT02107703)	1st or 2nd line, locally advanced or metastatic breast cancer, postmenopausal	Either no prior endocrine therapy or progression during or within 12 months of adjuvant endocrine therapy or greater than 12 months and progressed after first-line endocrine therapy for metastatic disease, or progression after first-line therapy (no adjuvant therapy)	abemaciclib + fulvestrant	ER+ and/or PR+ HER2–	550	July 2014
			placebo + fulvestrant			
Phase 2 (MONARCH 1, NCT02102490)	1st line or greater, locally advanced or metastatic breast cancer	Disease progression after anti-estrogen therapy and 2 prior chemotherapy regimens required	abemaciclib	ER+ and/or PR+ HER2–	128	June 2014
Phase 1 (NCT02057133)	1st line, locally advanced or metastatic breast cancer,	No prior prior systemic endocrine therapy	abemaciclib + letrozole	ER+ and/or PR+ HER2–	81	March 2014

	pre- or postmenopausal					
	1st line, locally advanced or metastatic breast cancer, pre- or postmenopausal	No prior prior systemic endocrine therapy	abemaciclib + anastrozole			
	1st line or greater, locally advanced or metastatic breast cancer, pre- or postmenopausal		abemaciclib + tamoxifen			
	2nd line or greater, locally advanced or metastatic breast cancer, pre- or postmenopausal	Prior therapy with anastrozole or letrozole required	abemaciclib + exemestane			
	2nd line or greater, locally advanced or metastatic breast cancer, pre- or postmenopausal	Prior therapy with anastrozole or letrozole required	abemaciclib + exemestane + everolimus			

NOTE: ER = estrogen receptor; HER2 = human epidermal growth factor receptor 2; PR = progesterone receptor; Pts = patients.

Summary

In conclusion, CDK 4/6 inhibitors are a promising therapy for ER+ metastatic breast cancer. The FDA has granted accelerated approval to palbociclib for use in combination with letrozole for first-line or initial treatment in postmenopausal women with ER+/HER2-negative advanced breast cancer. Ongoing trials are evaluating safety and efficacy of CDK 4/6 inhibitors alone and in combination with other therapies.

Slides (version 1)

MY CANCER GENOME®

GENETICALLY INFORMED CANCER MEDICINE

Navigate the presentation using any of the following:

- Use the buttons

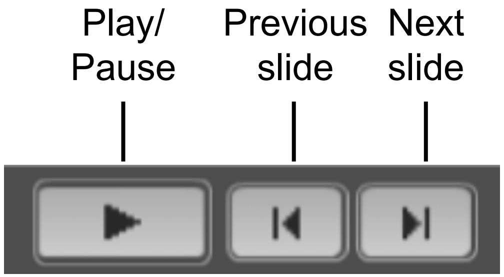

- Click on the slide

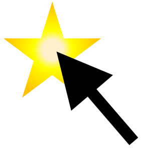

- Swipe (in mobile)

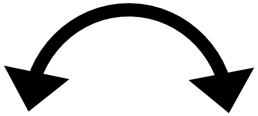

Overcoming Resistance to Endocrine Therapy in ER+ Breast Cancer

CDK4/6 Inhibition as a Novel Treatment Strategy

Prepared by My Cancer Genome at Vanderbilt University Medical Center

with funding from Pfizer Independent Grants for Learning & Change

Summer 2015

Current on 7/1/2015. © 2015 MyCancerGenome.org

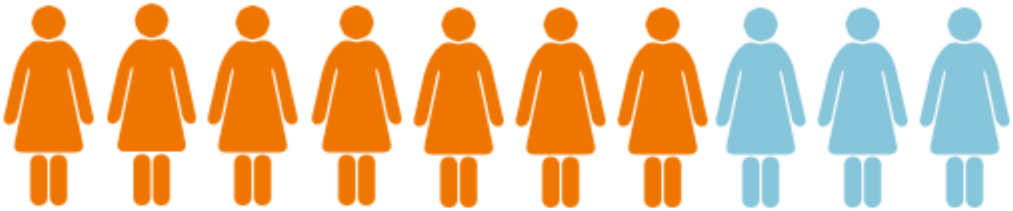

70% of breast cancer cases are estrogen receptor positive (ER+)

Allred et al. 2009
Colditz et al. 2004

Current on 7/1/2015. © 2015 MyCancerGenome.org

What is Estrogen?

- Estrogen is a steroid hormone

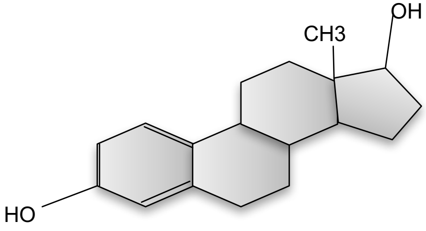

- Controls cellular processes, such as
  - cell division
  - growth
  - differentiation
  - proliferation

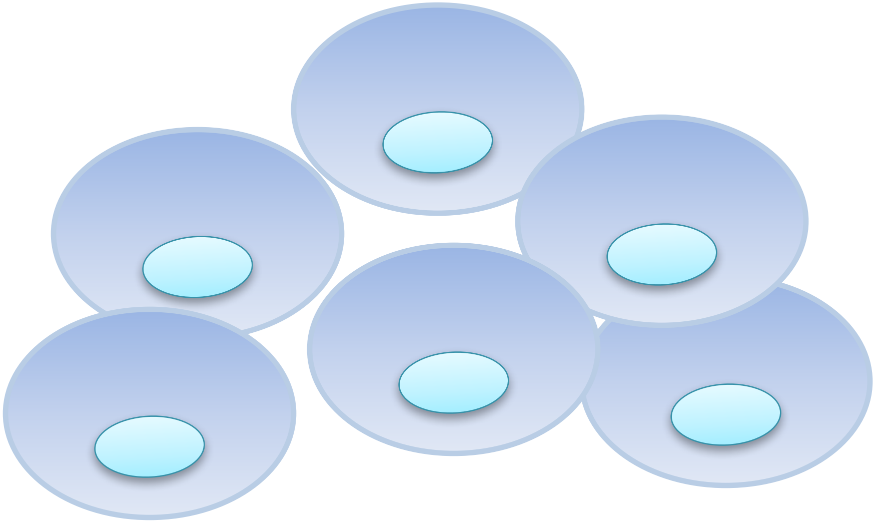

Current on 7/1/2015. © 2015 MyCancerGenome.org

Estrogen Receptor (ER) Signaling

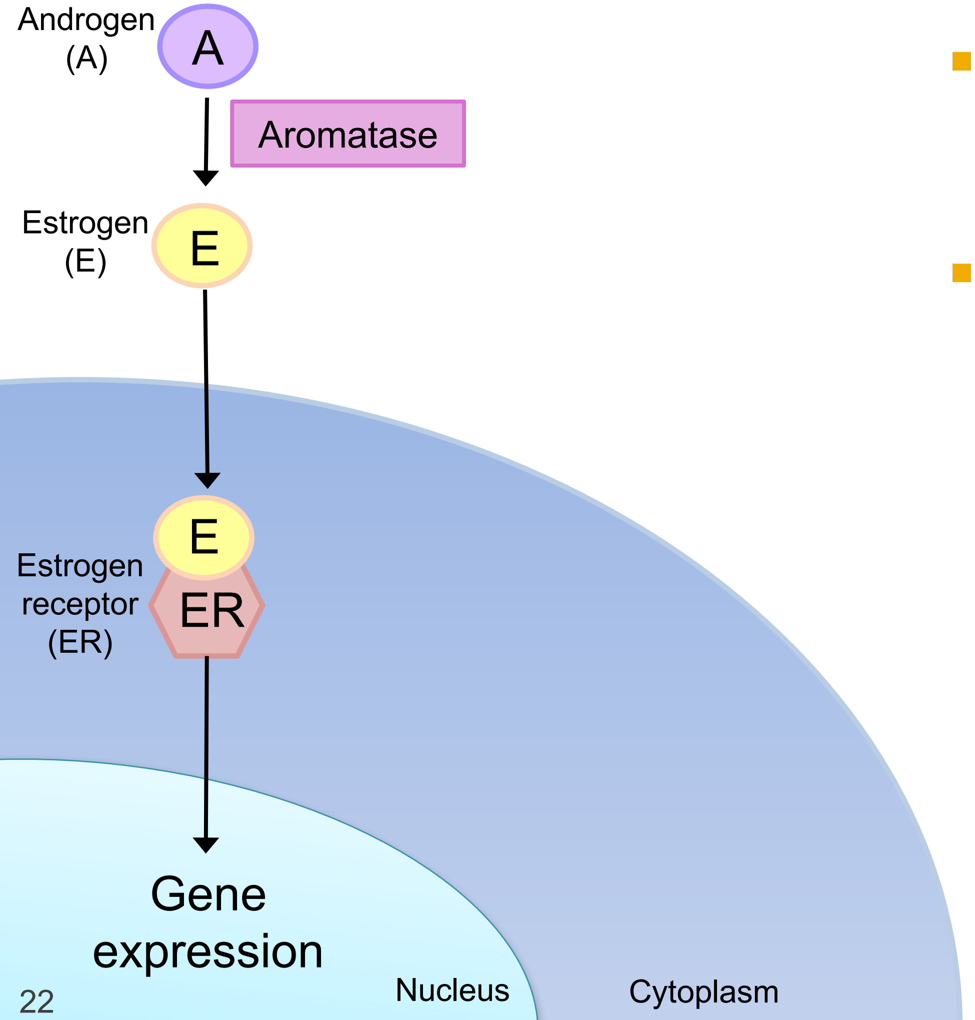

- **Aromatase** converts androgens into estrogens
- Estrogen binds to the estrogen receptor (ER), resulting in changes in **gene expression**

Current on 7/1/2015. © 2015 MyCancerGenome.org

Estrogen Receptor (ER) Signaling

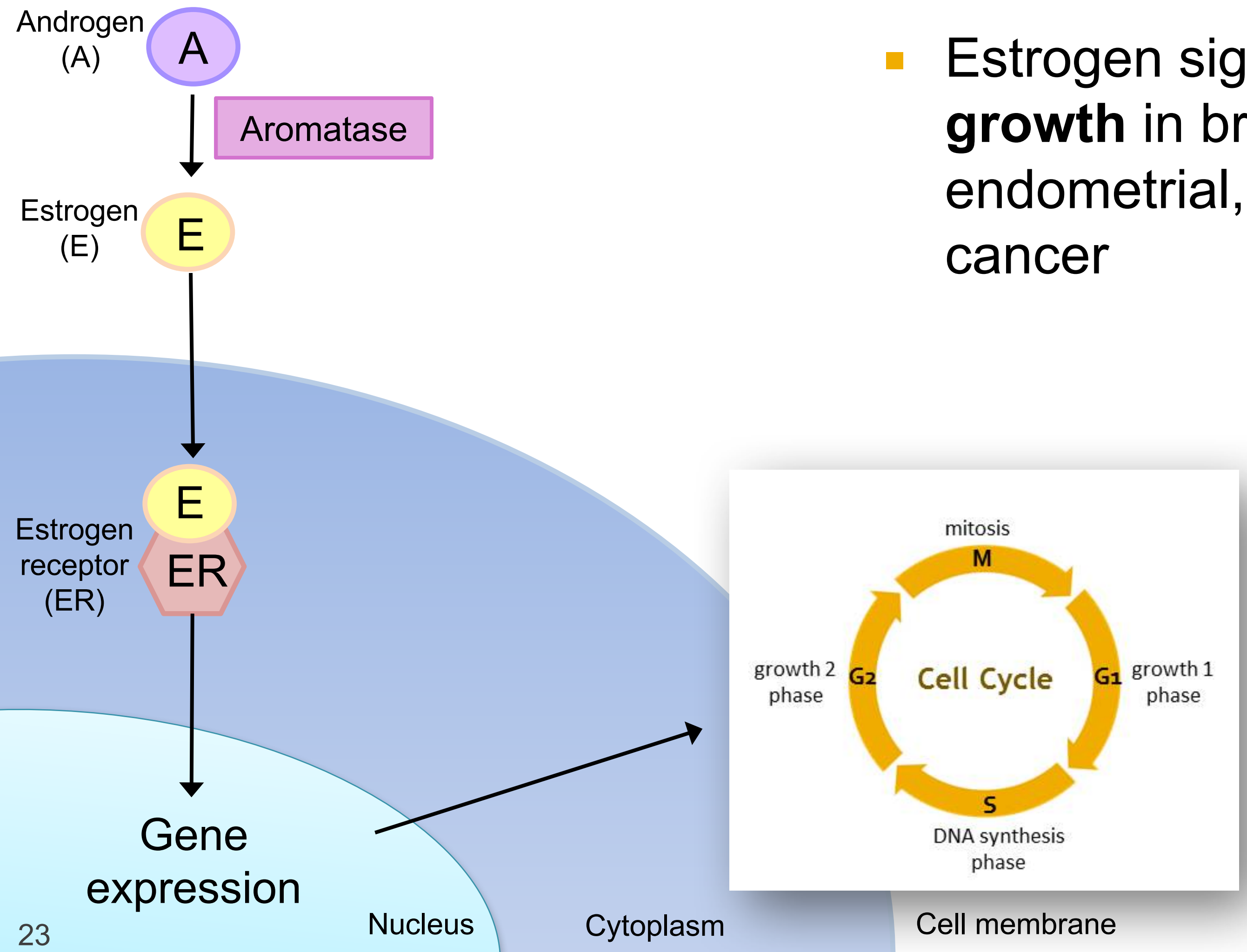

- Estrogen signaling **drives cell growth** in breast, endometrial, and prostate cancer

Current on 7/1/2015. © 2015 MyCancerGenome.org

Three Classes of Endocrine Agents Used to Treat ER+ Breast Cancer

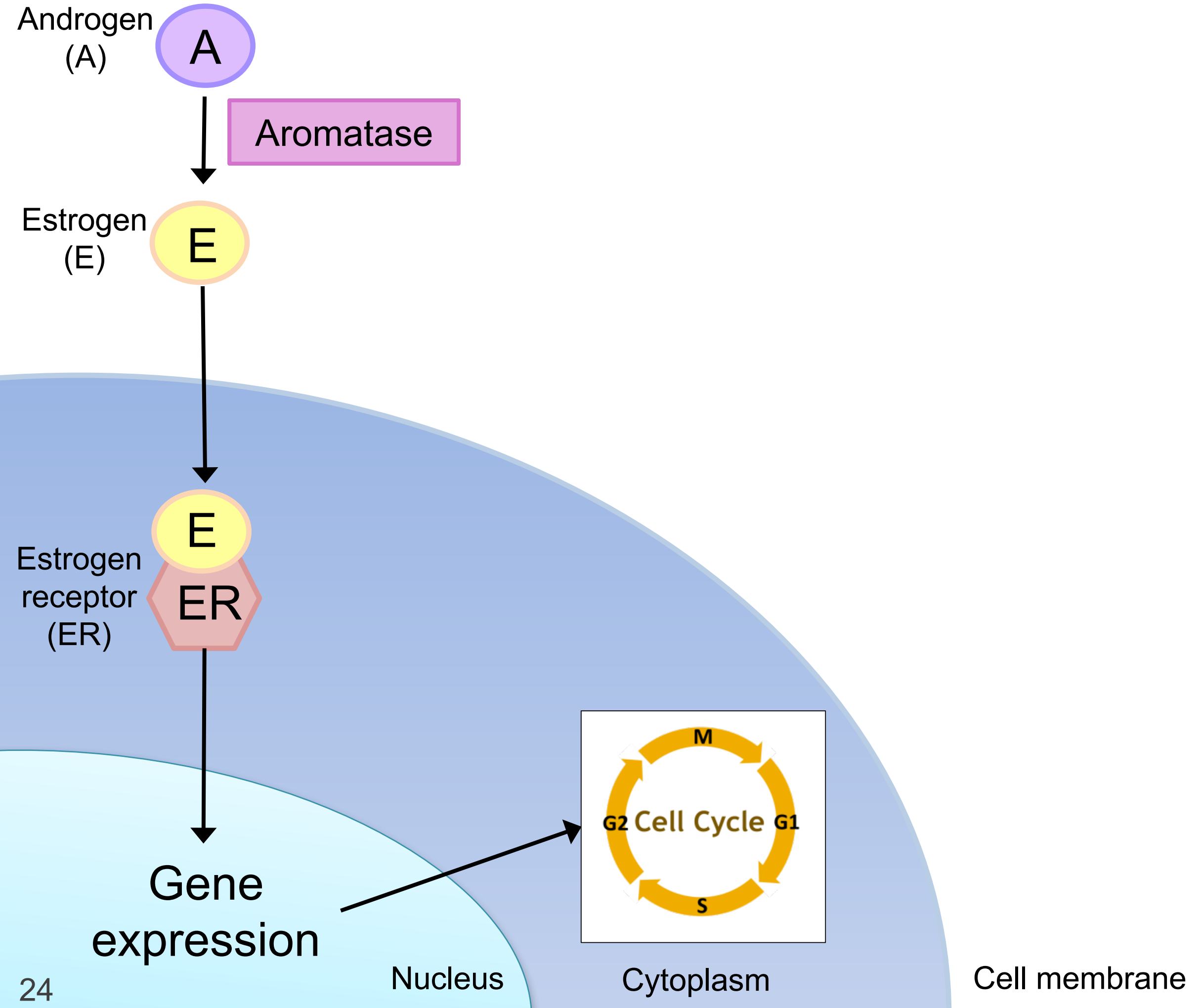

Current on 7/1/2015. © 2015 MyCancerGenome.org

Three Classes of Endocrine Agents Used to Treat ER+ Breast Cancer

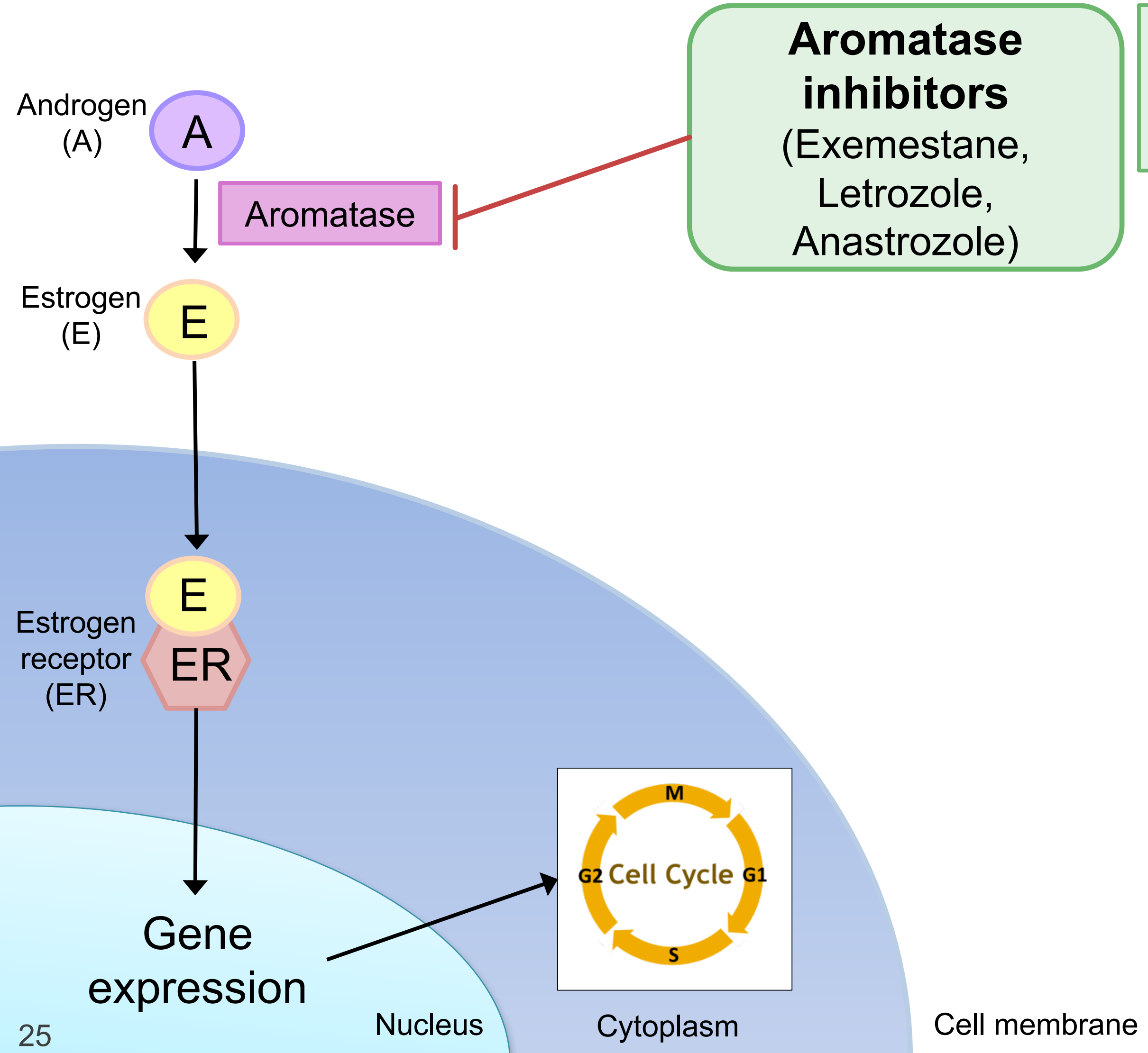

Aromatase inhibitors block aromatase, so estrogen is not produced

Current on 7/1/2015. © 2015 MyCancerGenome.org

Three Classes of Endocrine Agents Used to Treat ER+ Breast Cancer

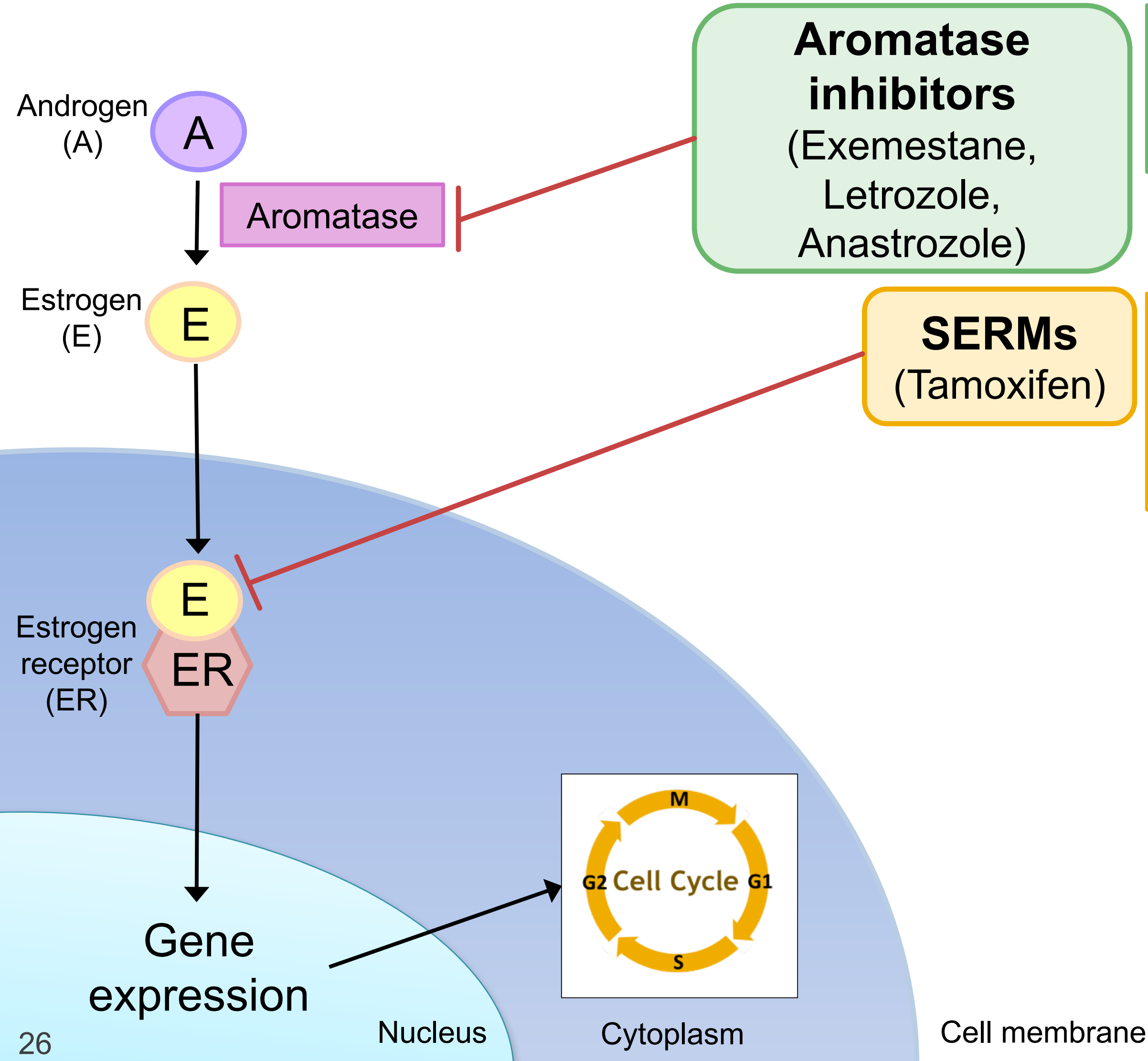

Aromatase inhibitors block aromatase, so estrogen is not produced

Selective estrogen receptor modulators (SERMs) compete with estrogen for binding to the ER

Current on 7/1/2015. © 2015 MyCancerGenome.org

Three Classes of Endocrine Agents Used to Treat ER+ Breast Cancer

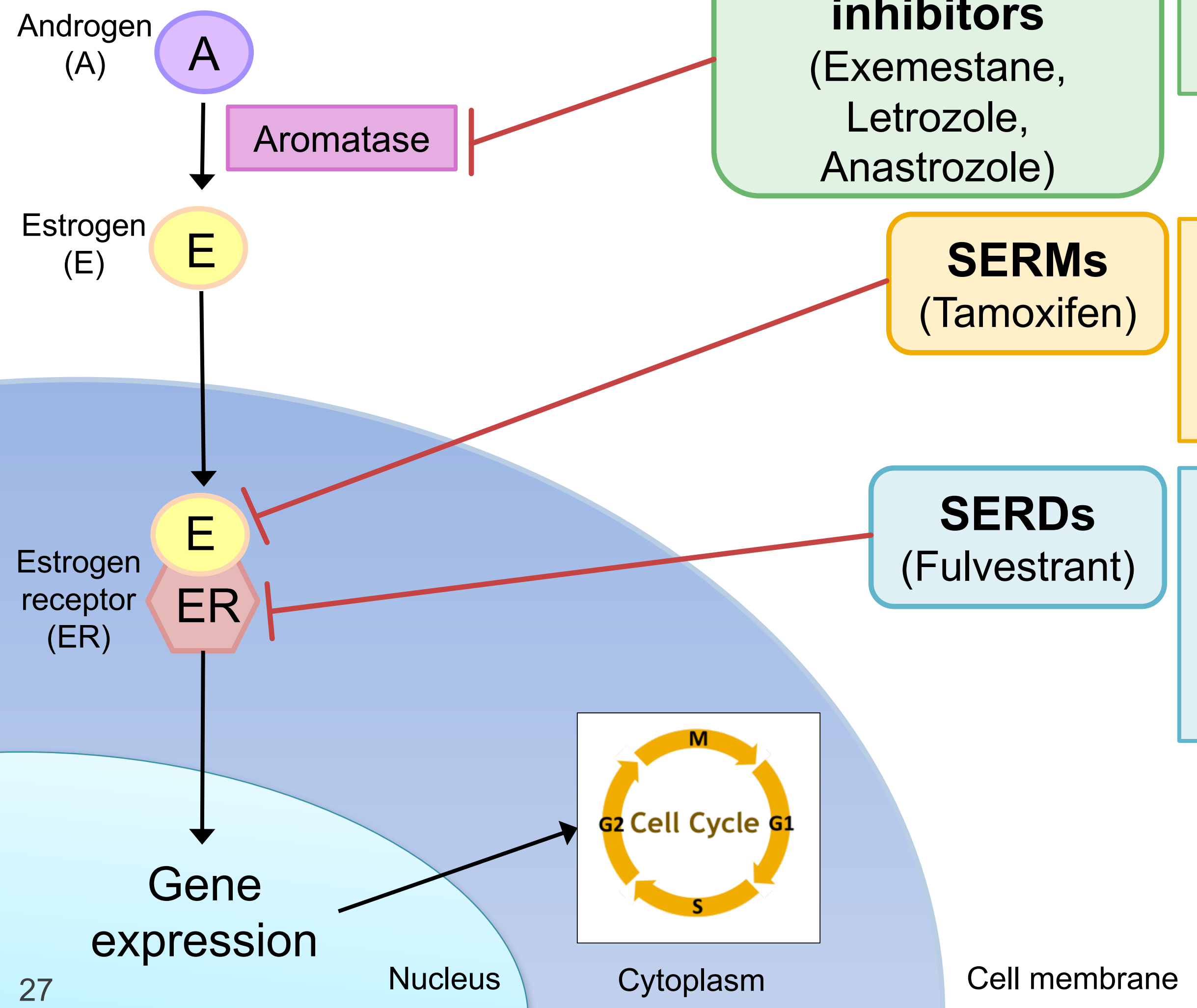

Aromatase inhibitors
(Exemestane, Letrozole, Anastrozole)

Aromatase inhibitors block aromatase, so estrogen is not produced

SERMs
(Tamoxifen)

Selective estrogen receptor modulators (SERMs) compete with estrogen for binding to the ER

SERDs
(Fulvestrant)

Selective estrogen receptor degraders or downregulators (SERDs) bind to the ER, blocking estrogen from binding, and sometimes downregulate the ER

Current on 7/1/2015. © 2015 MyCancerGenome.org

Primary Resistance to Endocrine Therapy in ER+ Breast Cancer

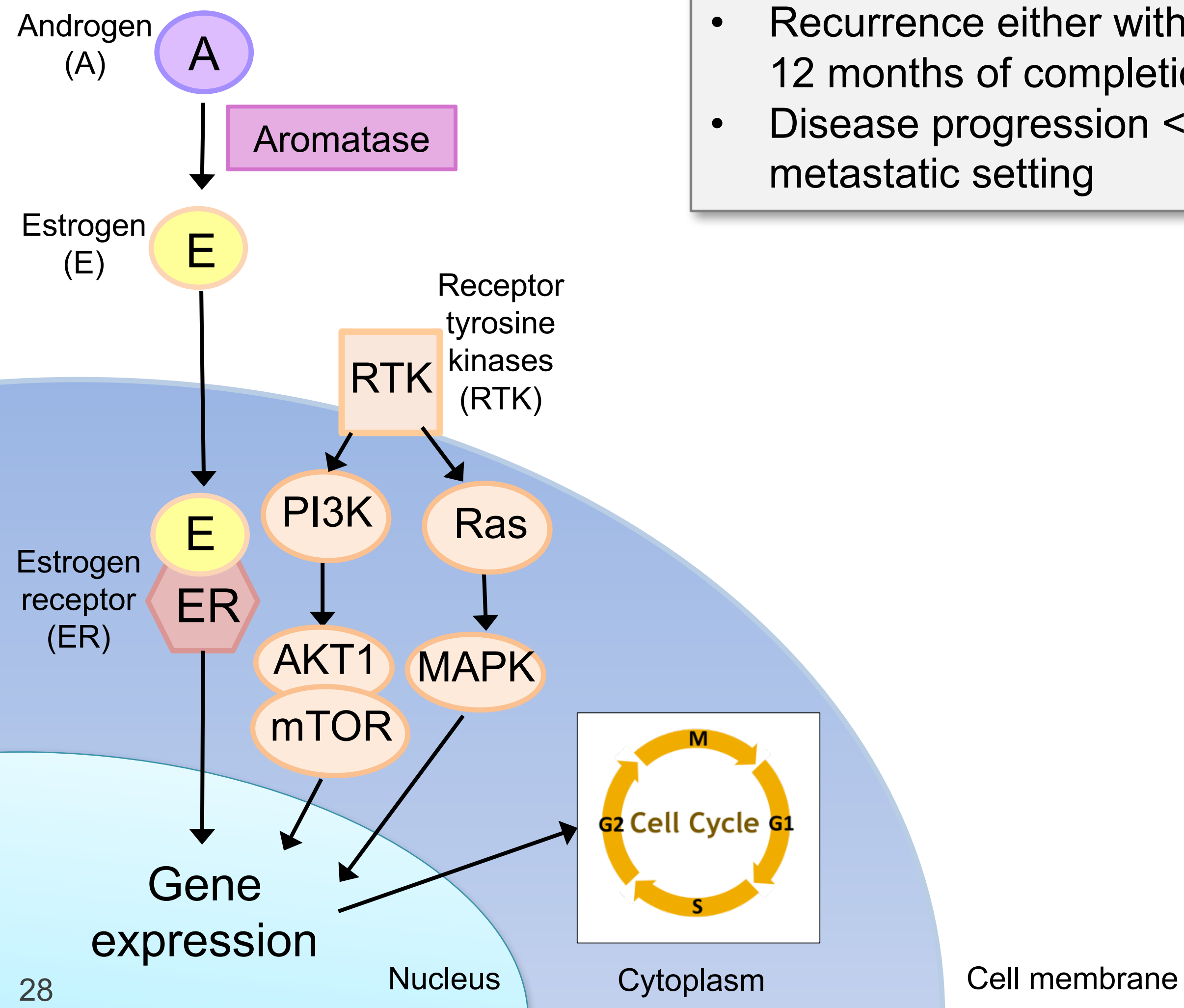

Primary resistance is defined as

- Recurrence either within adjuvant therapy or within 6–12 months of completion of adjuvant therapy
- Disease progression < 6 months after treatment in the metastatic setting

Bachelot et al. 2012; Osborne and Schiff 2011

Current on 7/1/2015. © 2015 MyCancerGenome.org

Primary Resistance to Endocrine Therapy in ER+ Breast Cancer

Primary resistance is defined as

- Recurrence either within adjuvant therapy or within 6–12 months of completion of adjuvant therapy
- Disease progression < 6 months after treatment in the metastatic setting

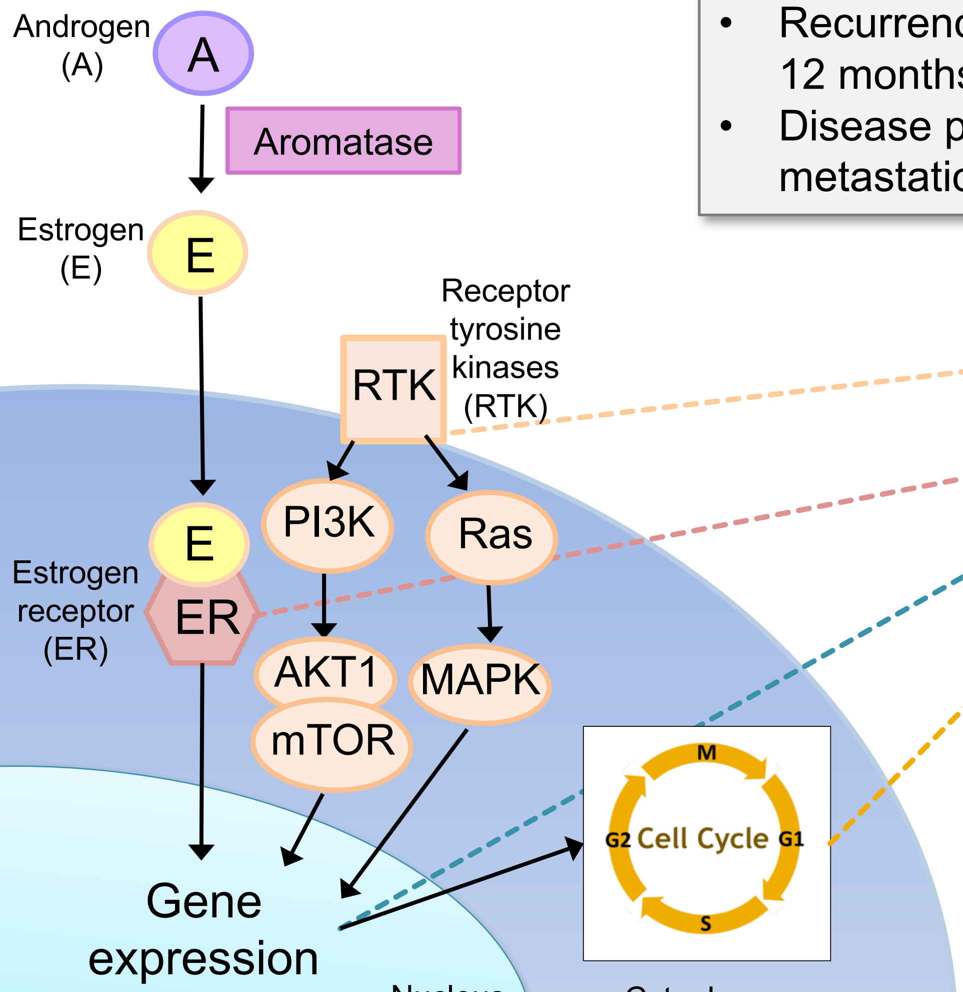

Some ways primary resistance may occur:

- FGFR amplifications
- Loss of ERα
- Post-translational modification of ERα
- ER mutations
- Expression of ER coactivation/corepression factors
- MYC amplification and overexpression
- Cyclin D1 amplification or expression

Cell membrane

Bachelot et al. 2012; Osborne and Schiff 2011

Current on 7/1/2015. © 2015 MyCancerGenome.org

Acquired Resistance to Endocrine Therapy in ER+ Breast Cancer

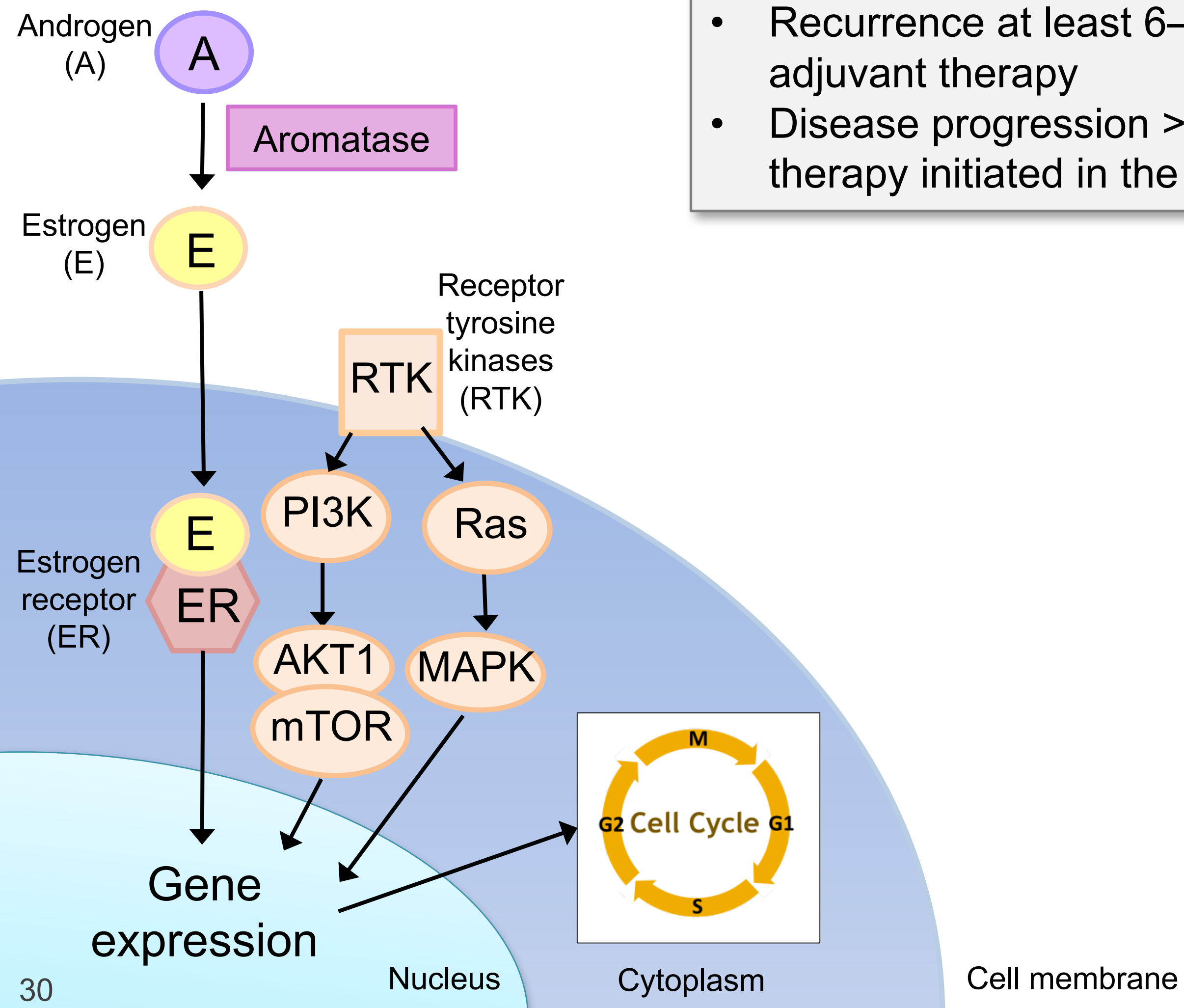

Acquired resistance is defined as

- Recurrence at least 6–12 months after completion of adjuvant therapy
- Disease progression > 6 months after endocrine therapy initiated in the metastatic setting

Bachelot et al. 2012; Osborne and Schiff 2011

Current on 7/1/2015. © 2015 MyCancerGenome.org

Acquired Resistance to Endocrine Therapy in ER+ Breast Cancer

Acquired resistance is defined as

- Recurrence at least 6–12 months after completion of adjuvant therapy
- Disease progression > 6 months after endocrine therapy initiated in the metastatic setting

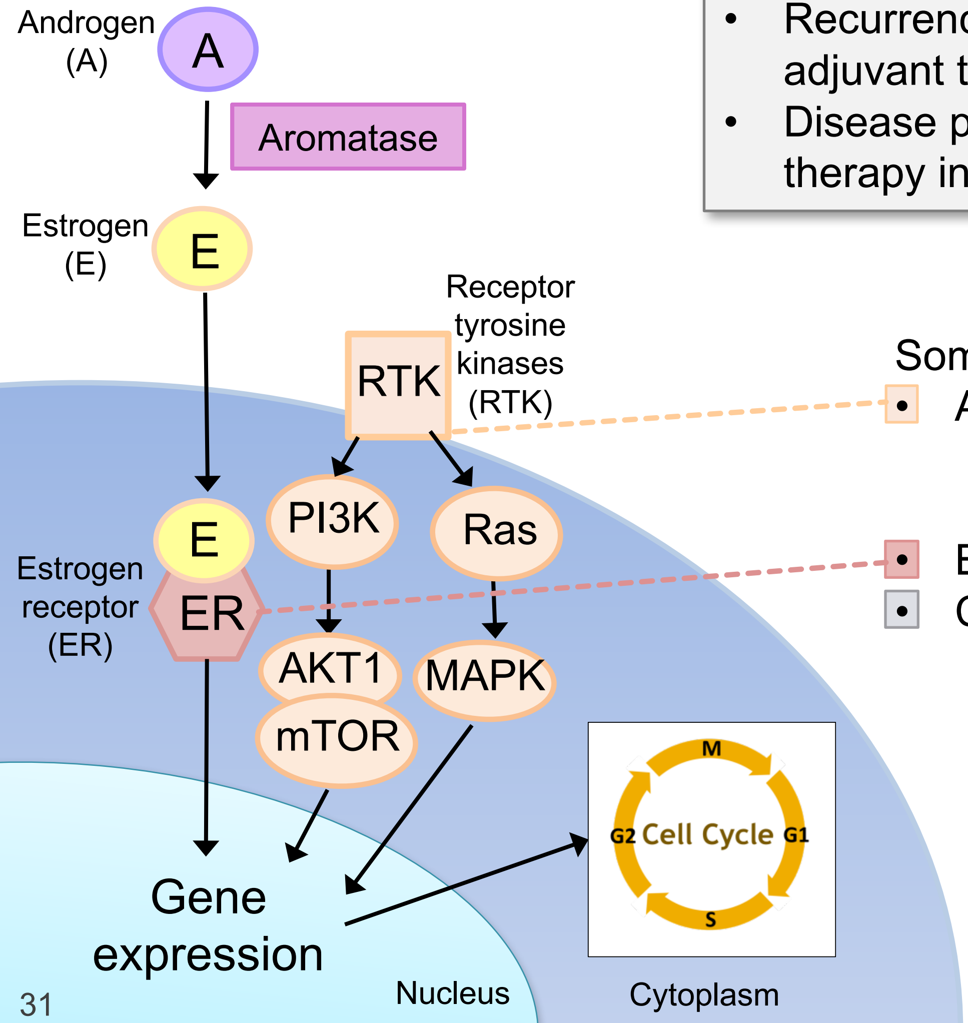

Some ways acquired resistance may occur:

- Activation of growth factor signaling pathways
  - PI3K/AKT1/mTOR
  - MAPK/ERK
- ER mutations
- Changes in the tumor microenvironment

Bachelot et al. 2012; Osborne and Schiff 2011

Current on 7/1/2015. © 2015 MyCancerGenome.org

Targeted Therapeutic Strategies in Endocrine Resistant ER+ Breast Cancer

Drug class	Strength of Clinical Data	Evidence	Clinical trials
mTOR inhibitors	1 positive phase 3 in second line<br>1 negative phase 3 in first line	Strong evidence for everolimus	Everolimus (Afinitor) approved (+ exemestane)<br>~53 trials with everolimus
CDK 4/6 inhibitors	1 positive phase 2 RCT in first line<br>1 positive phase 3 RCT in second line	Strong evidence for palbociclib/ Promising	Palbociclib (Ibrance) approved (+ letrozole)<br>Palbociclib + Fulvestrant trial stopped at interim for efficacy<br>Palbociclib, ribociclib, and abemaciclib in phase 3 trials
HDAC inhibitors	1 positive phase 2 in second line	Promising	Entinostat phase 3 trial initiated
EGFR/HER2 inhibitors	1 negative phase 2 RCT in second line (Gefitinib)<br>1 promising phase 2 (Erlotinib 1st-line)	Mixed	Erlotinib phase 2 trial ongoing (+ fulvestrant)
IGF-1R inhibitors	1 negative phase 2 in second line (agent: OSI-906)	Not known	Cixutumumab (IMC-A12) phase 1/2 trial ongoing
FGFR inhibitors	N/A	Not known	Dovitinib and nintedanib phase 2 trials ongoing
PI3K/Akt inhibitors	N/A	Not known	Buparlisib (BKM120) phase 3 trial ongoing
Angiogenesis inhibitors	1 negative phase 2 RCT	Poor	Bevacizumab in phase 3 trials; motesanib phase 2 trials planned

Current on 7/1/2015. © 2015 MyCancerGenome.org

Targeted Therapeutic Strategies in Endocrine Resistant ER+ Breast Cancer

Drug class	Strength of Clinical Data	Evidence	Clinical trials
mTOR inhibitors	1 positive phase 3 in second line<br>1 negative phase 3 in first line	Strong evidence for everolimus	Everolimus (Afinitor) approved (+ exemestane)<br>~53 trials with everolimus
CDK 4/6 inhibitors	1 positive phase 2 RCT in first line<br>1 positive phase 3 RCT in second line	Strong evidence for palbociclib/ Promising	Palbociclib (Ibrance) approved (+ letrozole)<br>Palbociclib + Fulvestrant trial stopped at interim for efficacy<br>Palbociclib, ribociclib, and abemaciclib in phase 3 trials
HDAC inhibitors	1 positive phase 2 in second line	Promising	Entinostat phase 3 trial initiated
EGFR/HER2 inhibitors	1 negative phase 2 RCT in second line (Gefitinib)<br>1 promising phase 2 (Erlotinib 1st-line)	Mixed	Erlotinib phase 2 trial ongoing (+ fulvestrant)
IGF-1R inhibitors	1 negative phase 2 in second line (agent: OSI-906)	Not known	Cixutumumab (IMC-A12) phase 1/2 trial ongoing
FGFR inhibitors	N/A	Not known	Dovitinib and nintedanib phase 2 trials ongoing
PI3K/Akt inhibitors	N/A	Not known	Buparlisib (BKM120) phase 3 trial ongoing
Angiogenesis inhibitors	1 negative phase 2 RCT	Poor	Bevacizumab in phase 3 trials; motesanib phase 2 trials planned

Current on 7/1/2015. © 2015 MyCancerGenome.org

Targeted Therapeutic Strategies in Endocrine Resistant ER+ Breast Cancer

Drug class	Strength of Clinical Data	Evidence	Clinical trials
mTOR inhibitors	1 positive phase 3 in second line<br>1 negative phase 3 in first line	Strong evidence for everolimus	Everolimus (Afinitor) approved (+ exemestane)<br>~53 trials with everolimus
CDK 4/6 inhibitors	1 positive phase 2 RCT in first line<br>1 positive phase 3 RCT in second line	Strong evidence for palbociclib/<br>Promising	Palbociclib (Ibrance) approved (+ letrozole)<br>Palbociclib + Fulvestrant trial stopped at interim for efficacy<br>Palbociclib, ribociclib, and abemaciclib in phase 3 trials
HDAC inhibitors	1 positive phase 2 in second line	Promising	Entinostat phase 3 trial initiated
EGFR/HER2 inhibitors	1 negative phase 2 RCT in second line (Gefitinib)<br>1 promising phase 2 (Erlotinib 1st-line)	Mixed	Erlotinib phase 2 trial ongoing (+ fulvestrant)
IGF-1R inhibitors	1 negative phase 2 in second line (agent: OSI-906)	Not known	Cixutumumab (IMC-A12) phase 1/2 trial ongoing
FGFR inhibitors	N/A	Not known	Dovitinib and nintedanib phase 2 trials ongoing
PI3K/Akt inhibitors	N/A	Not known	Buparlisib (BKM120) phase 3 trial ongoing
Angiogenesis inhibitors	1 negative phase 2 RCT	Poor	Bevacizumab in phase 3 trials; motesanib phase 2 trials planned

Current on 7/1/2015. © 2015 MyCancerGenome.org

Combining Everolimus (mTOR Inhibition) with Exemestane for ER+ Metastatic Breast Cancer Resistant to Letrozole or Anastrazole

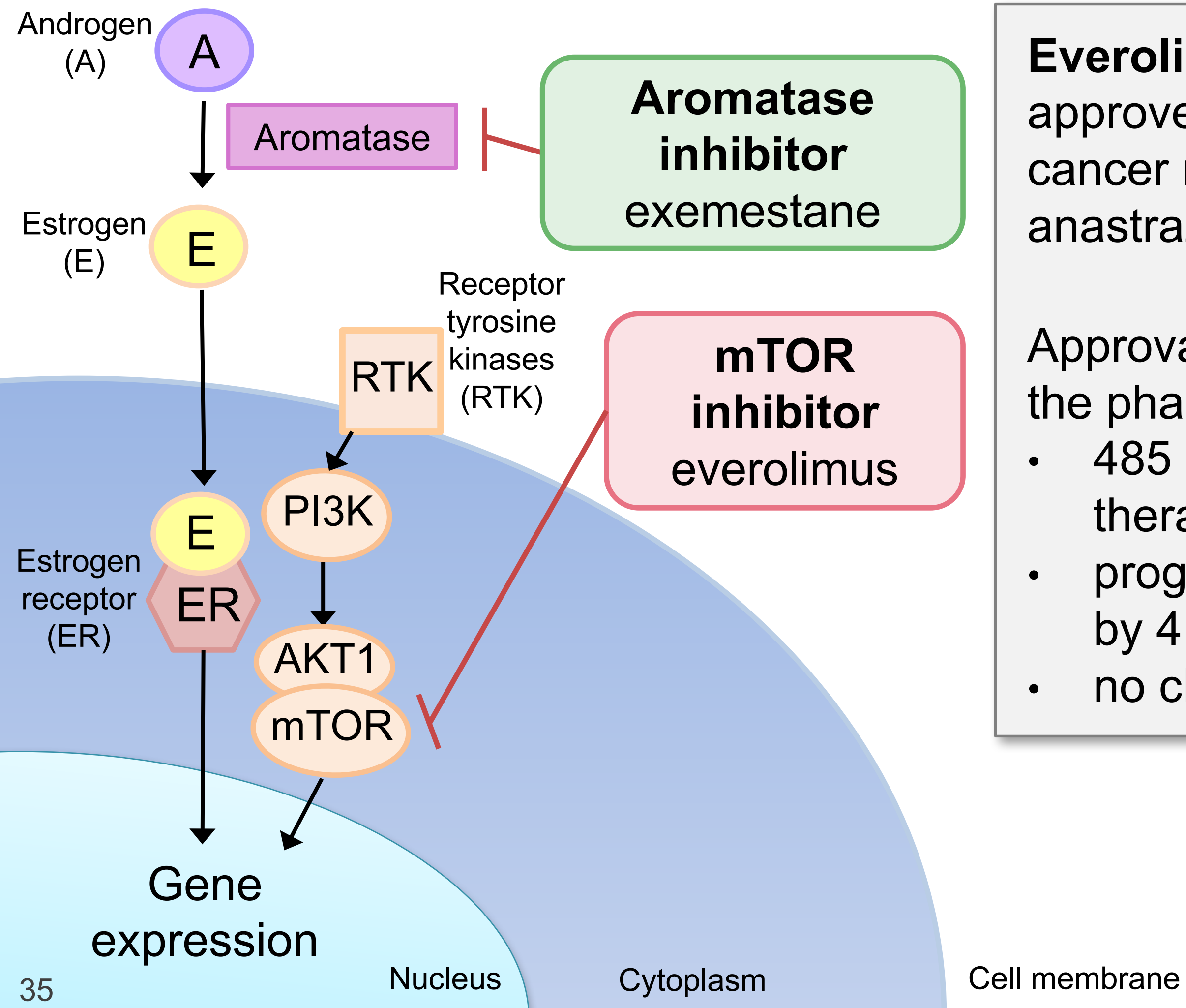

Everolimus + exemestane is FDA-approved for ER+ metastatic breast cancer **resistant** to letrozole or anastrazole

Approval was based on the results of the phase 3 trial BOLERO-2

- 485 patients received combination therapy
- progression free survival improved by 4.6 months
- no change in overall survival

Baselga et al. 2012
Piccart et al. 2014
Yardley et al. 2013

Current on 7/1/2015. © 2015 MyCancerGenome.org

Using Cyclin-Dependent Kinase (CDK4/6) Inhibitors to Overcome Resistance to Endocrine Agents

- Regulation of the cell growth cycle by Cyclin D1 and CDK4/6
- Combining CDK4/6 inhibitors with hormonal agents
- Reported and ongoing trials with CDK4/6 inhibitors

Current on 7/1/2015. © 2015 MyCancerGenome.org

Cyclin D1 Signaling

- **Cyclin D1** is a transcriptional target of the estrogen receptor
- The estrogen receptor regulates the expression of cyclin D1
- As a result, cyclin D1 overexpression and amplification is a mechanism of resistance to endocrine agents

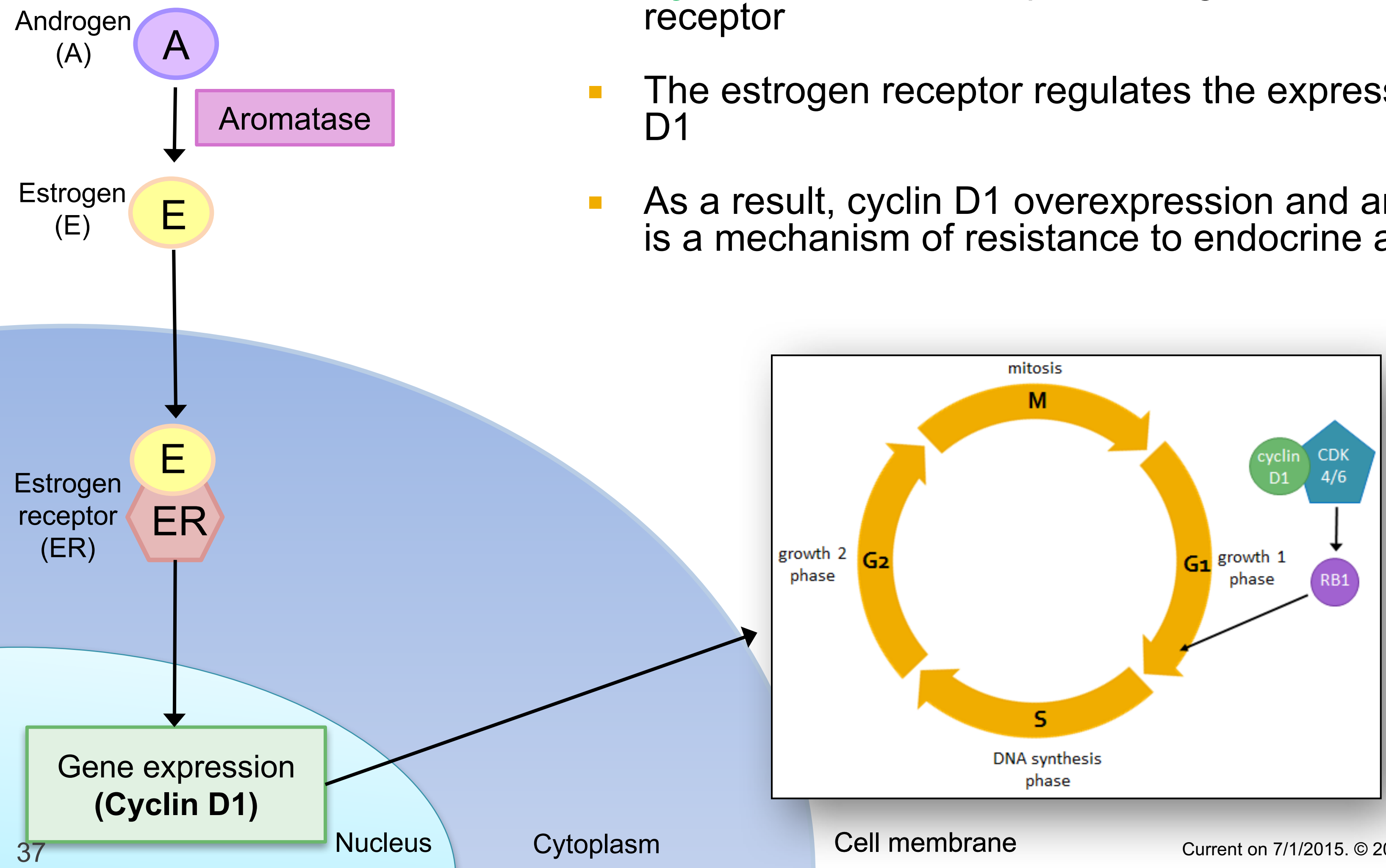

Current on 7/1/2015. © 2015 MyCancerGenome.org

Cyclin D1 Regulates the Cell Growth Cycle

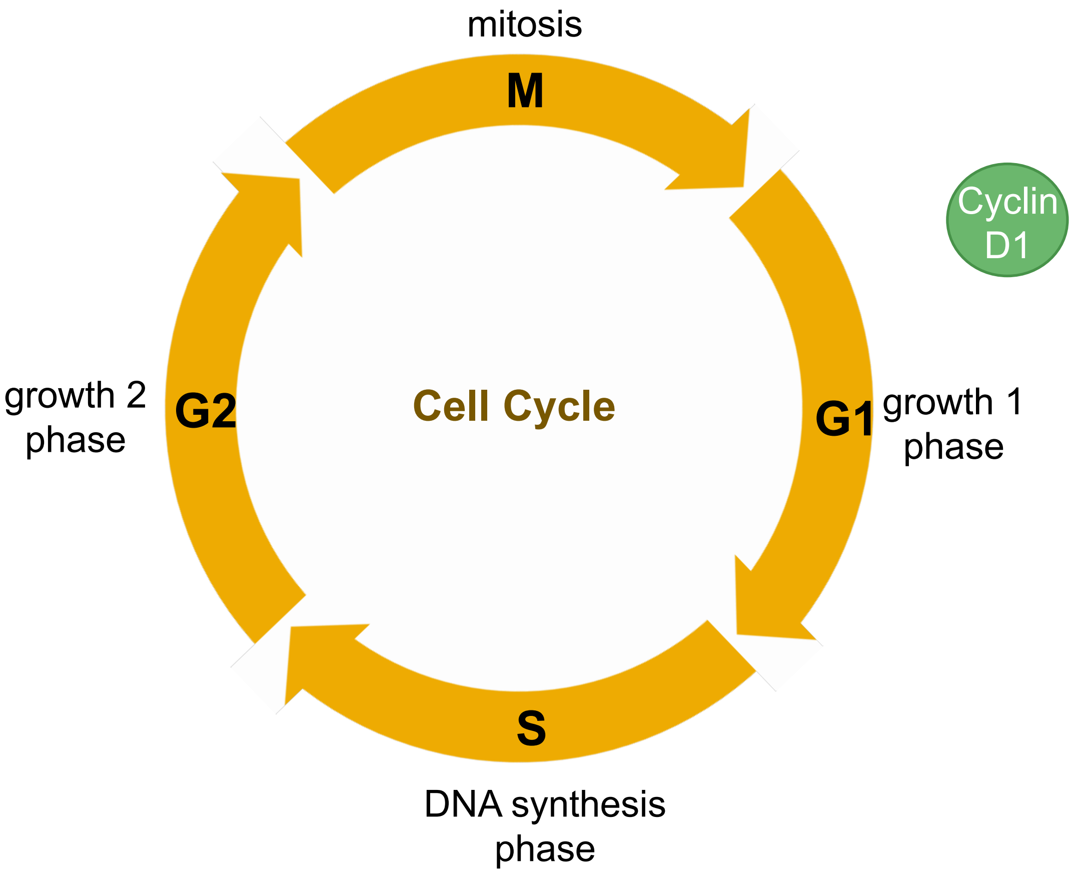

Current on 7/1/2015. © 2015 MyCancerGenome.org

Cyclin D1 Regulates the Cell Growth Cycle

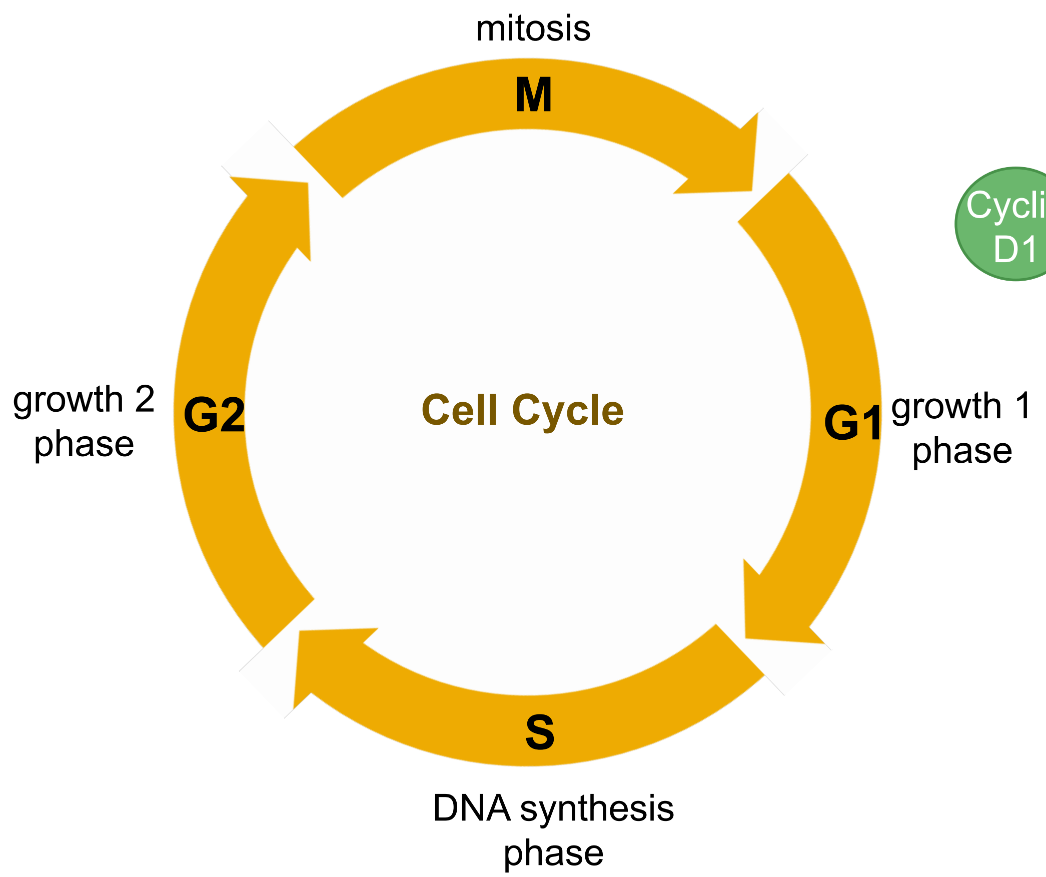

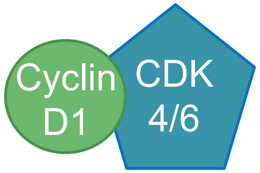

Cyclin D1 forms complexes with **CDK4/6**

Abbreviations:
CDK4/6: cyclin dependent kinase 4/6

Current on 7/1/2015. © 2015 MyCancerGenome.org

Cyclin D1 Regulates the Cell Growth Cycle

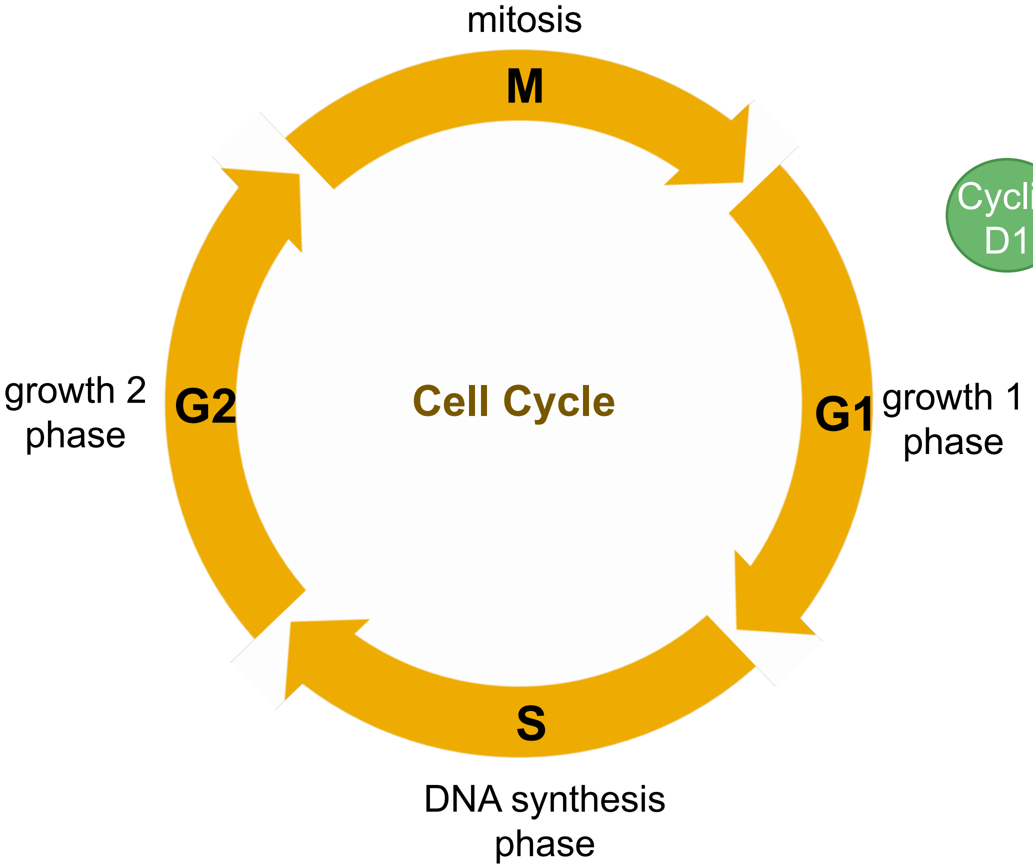

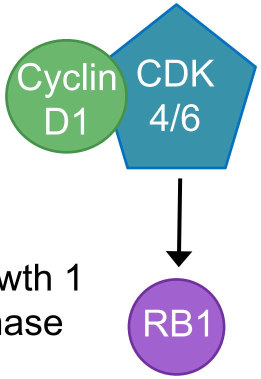

Cyclin D1 forms complexes with **CDK4/6**

CDK4/6 phosphorylates **RB1**, blocking its tumor suppressor function

Abbreviations:
CDK4/6: cyclin dependent kinase 4/6
RB1: retinoblastoma tumor suppressor protein 1

Current on 7/1/2015. © 2015 MyCancerGenome.org

Cyclin D1 Regulates the Cell Growth Cycle

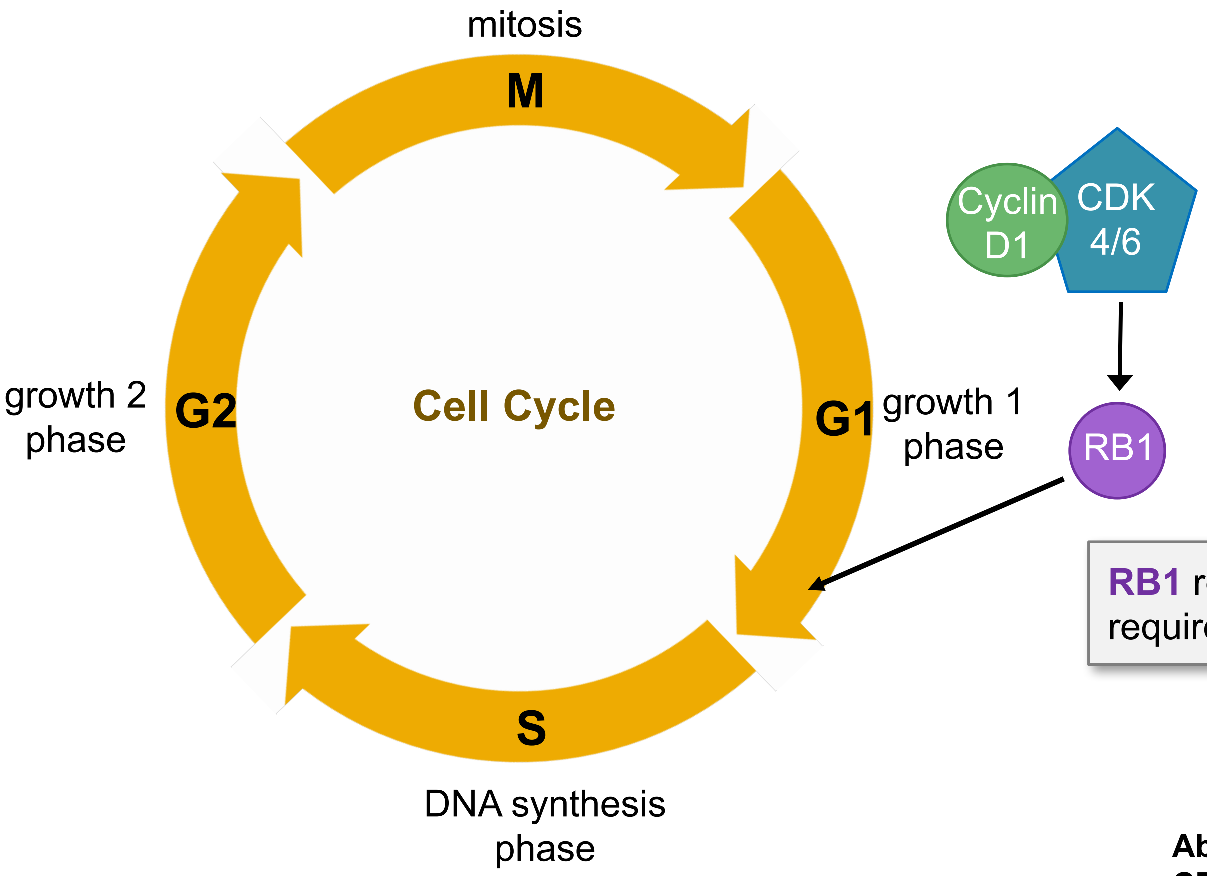

Cyclin D1 forms complexes with **CDK4/6**

CDK4/6 phosphorylates **RB1**, blocking its tumor suppressor function

RB1 releases the transcription factors required for entry into the **S phase**

Abbreviations:
CDK4/6: cyclin dependent kinase 4/6
RB1: retinoblastoma tumor suppressor protein 1

Current on 7/1/2015. © 2015 MyCancerGenome.org

Resistance to Endocrine Therapy via Alterations in Regulators of the Cell Growth Cycle

Alterations that cause the secondary signals in the estrogen signaling pathway to become activated include:

- **Cyclin D1** amplification
- **CDK4/6** gain of function
- **RB1** loss of function

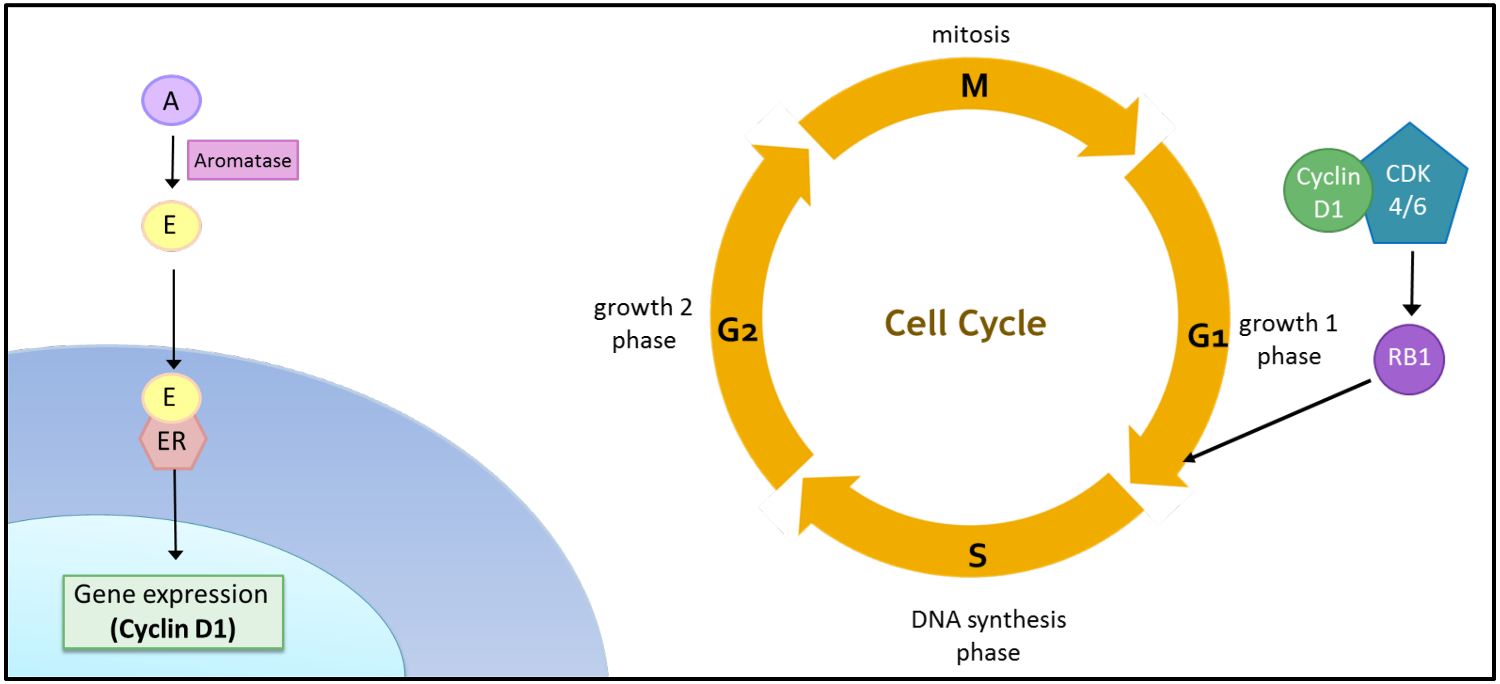

Butt et al. 2005
Giuliano et al. 2011
Migliaccio et al. 2014

Current on 7/1/2015. © 2015 MyCancerGenome.org

CDK4/6 Inhibition

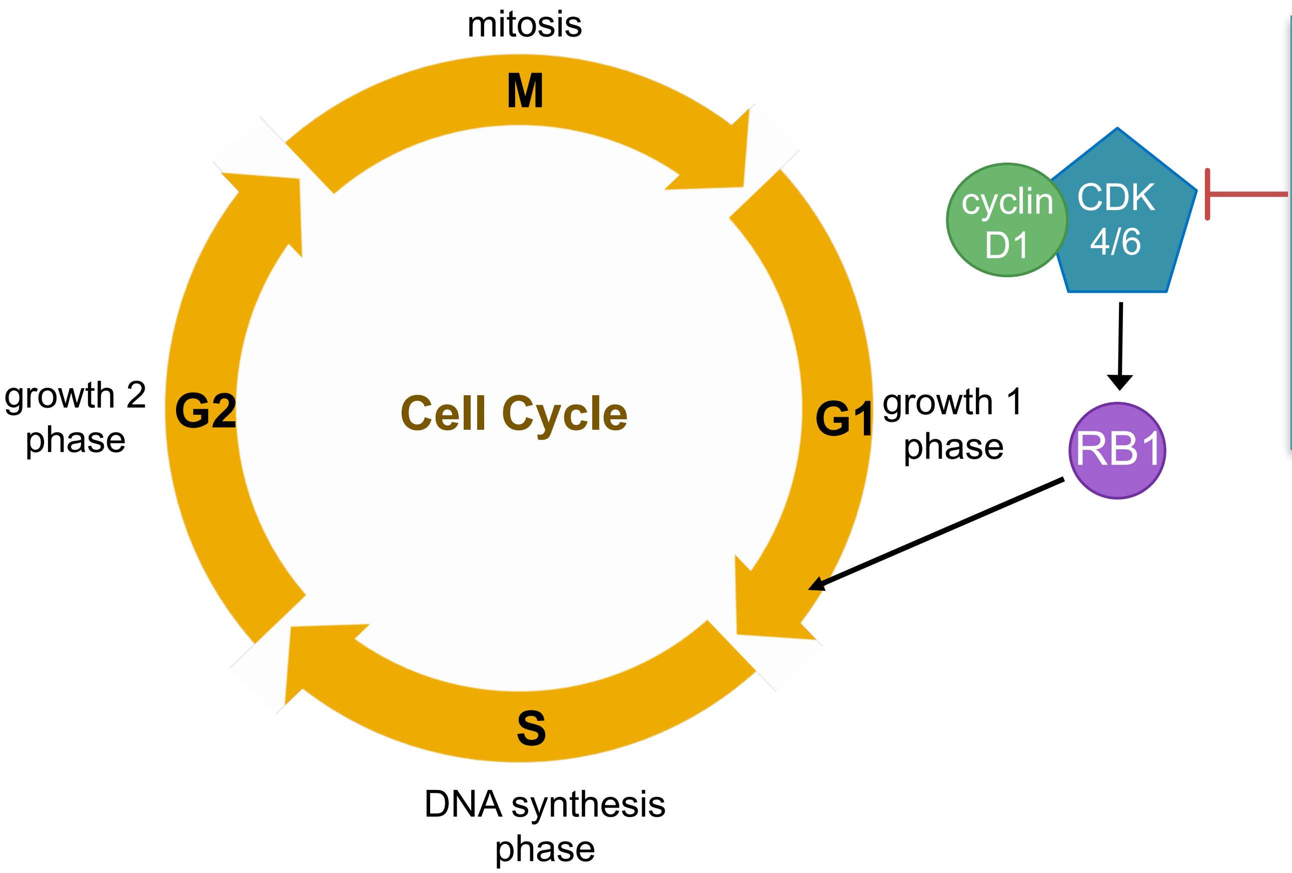

CDK 4/6 inhibitors:
Inhibition of CDK4/6 is an investigational therapeutic strategy in the adjuvant and neoadjuvant settings, as 1st line therapy, and after resistance develops to endocrine therapy.

Current on 7/1/2015. © 2015 MyCancerGenome.org

Retinoblastoma (RB1) Mutation Status and Impact of CDK4/6 Inhibitors

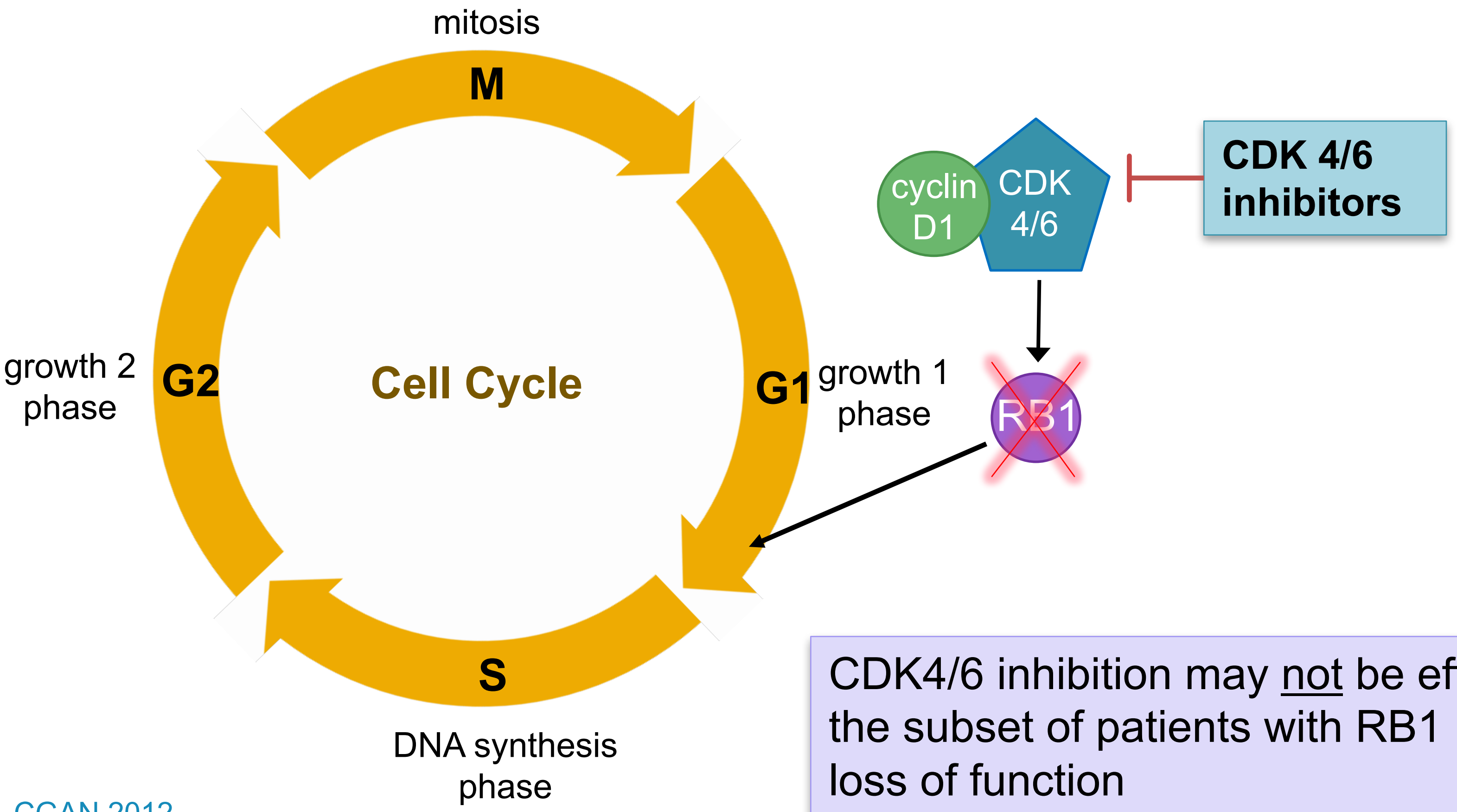

CDK4/6 inhibition may not be effective in the subset of patients with RB1 mutation or loss of function

CGAN 2012
Jiang et al. 2011

Current on 7/1/2015. © 2015 MyCancerGenome.org

Combining Hormonal Agents and CDK4/6 Inhibition

Combining ER-targeting agents with CDK4/6 inhibitors may improve benefit over standard of care

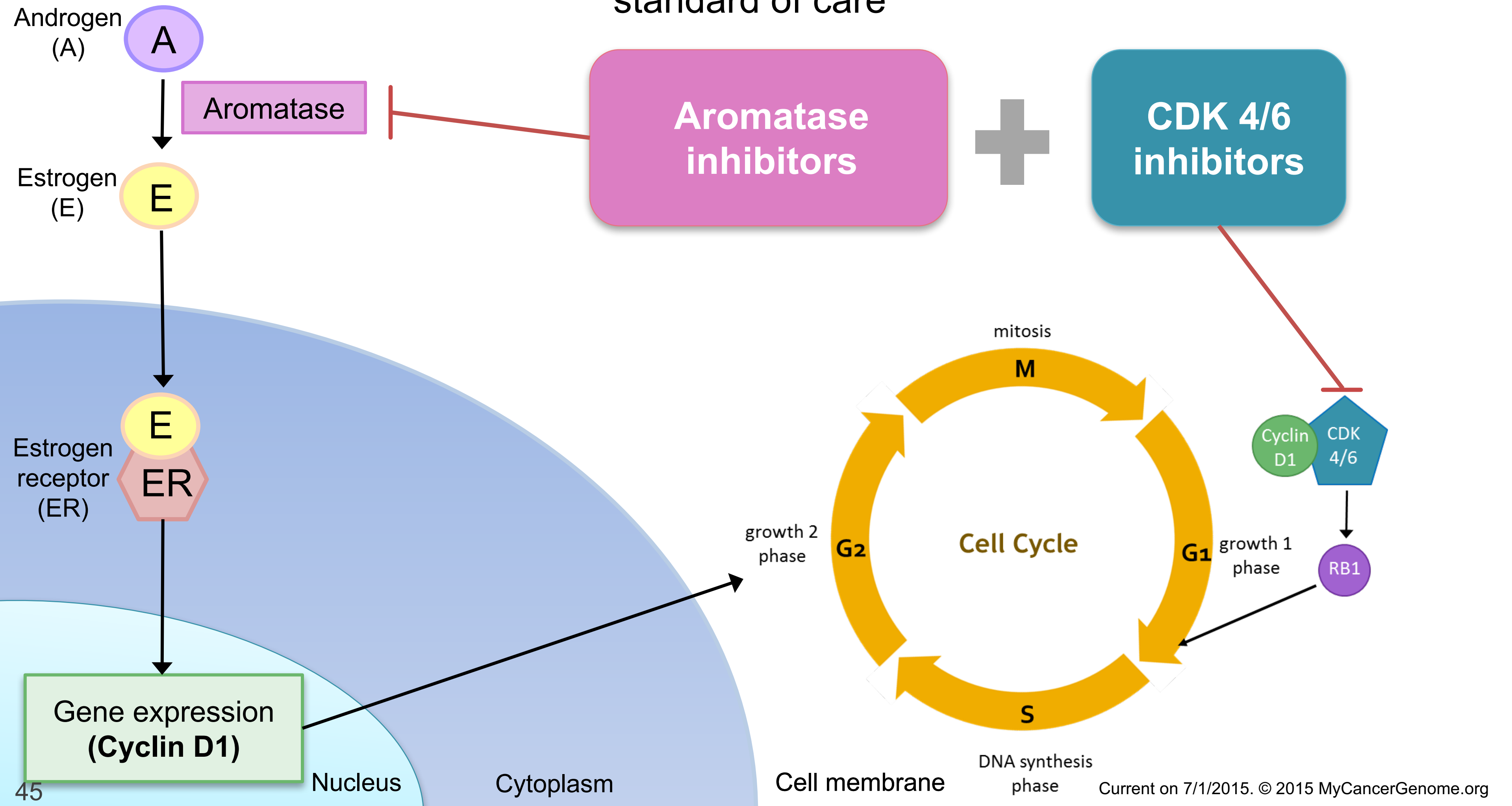

Current on 7/1/2015. © 2015 MyCancerGenome.org

Combining Hormonal Agents and CDK4/6 Inhibition

Combining ER-targeting agents with CDK4/6 inhibitors may improve benefit over standard of care

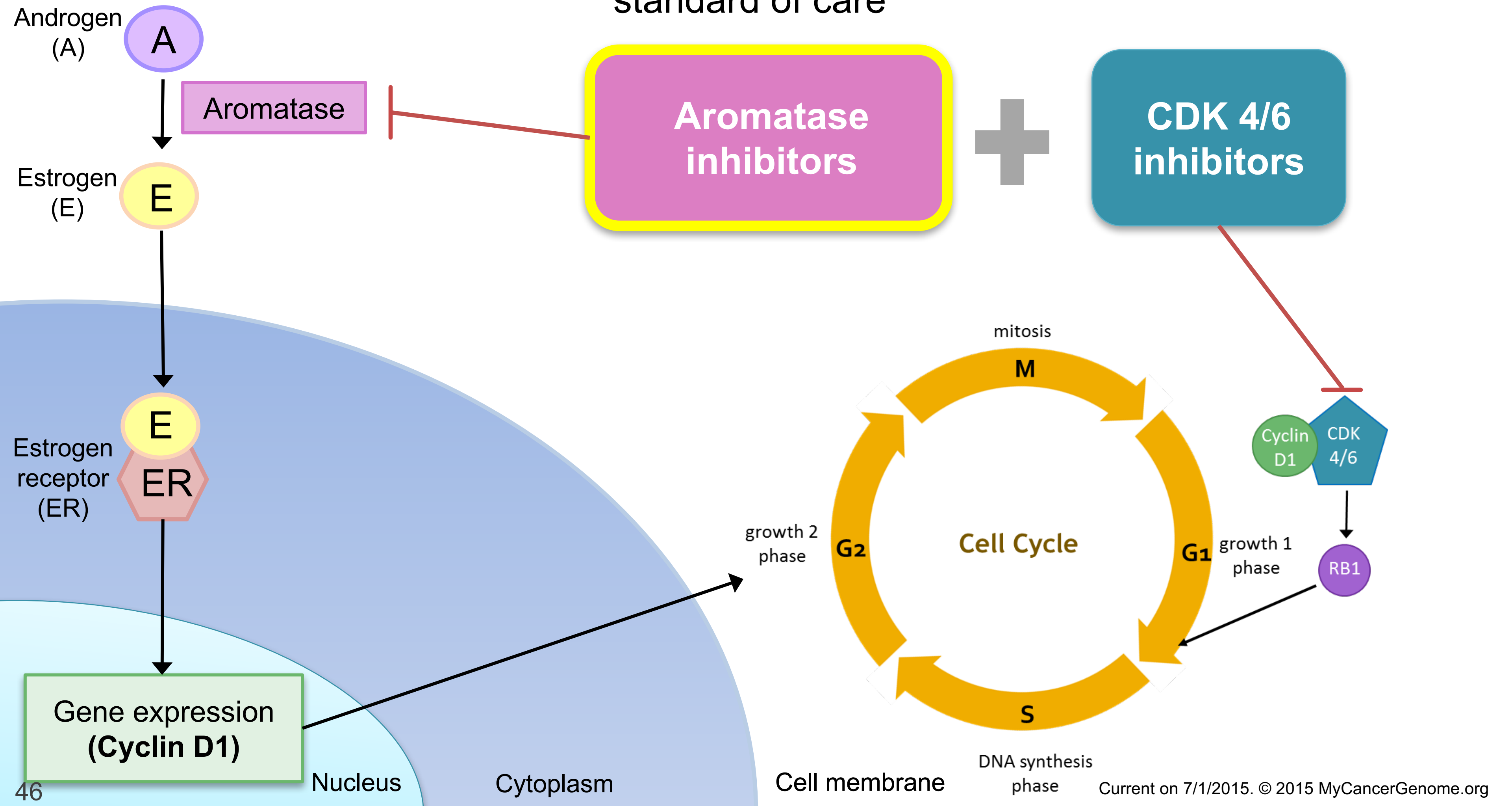

Current on 7/1/2015. © 2015 MyCancerGenome.org

Combining Hormonal Agents and CDK4/6 Inhibition

Combining ER-targeting agents with CDK4/6 inhibitors may improve benefit over standard of care

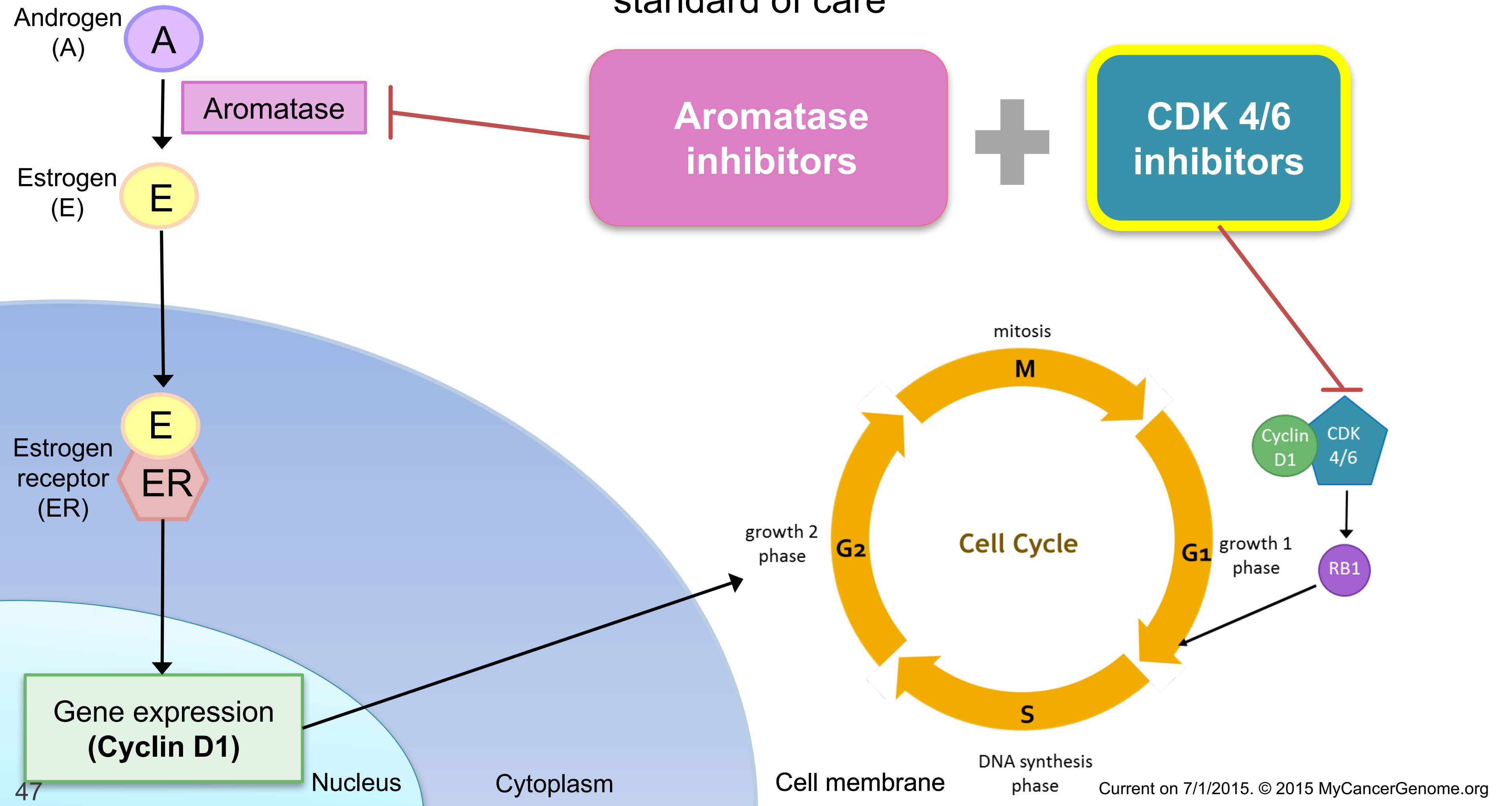

Current on 7/1/2015. © 2015 MyCancerGenome.org

CDK4/6 Inhibitors in Phase 3 Clinical Trials

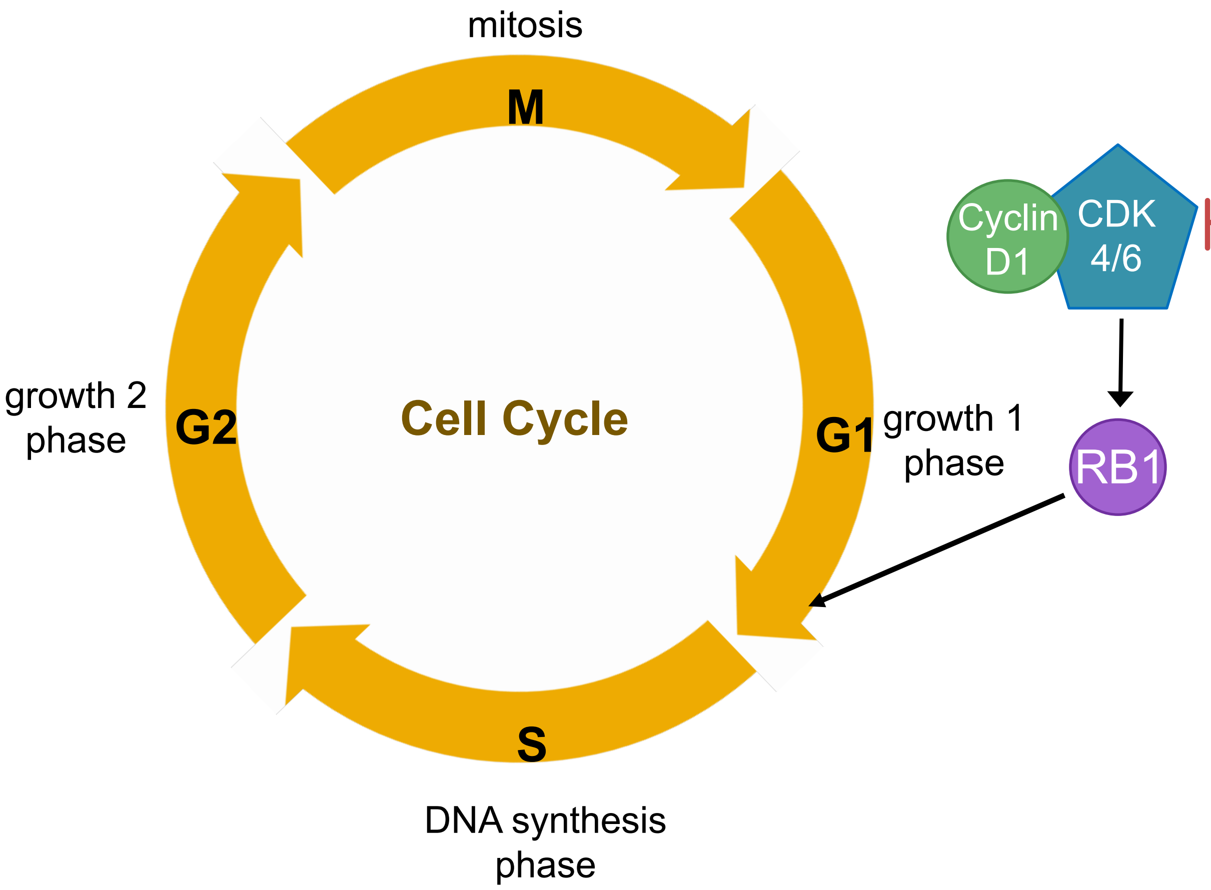

CDK 4/6 inhibitors:

- Palbociclib
- Ribociclib (LEE011)
- Abemaciclib

Current on 7/1/2015. © 2015 MyCancerGenome.org

CDK4/6 Inhibitors in Phase 3 Clinical Trials

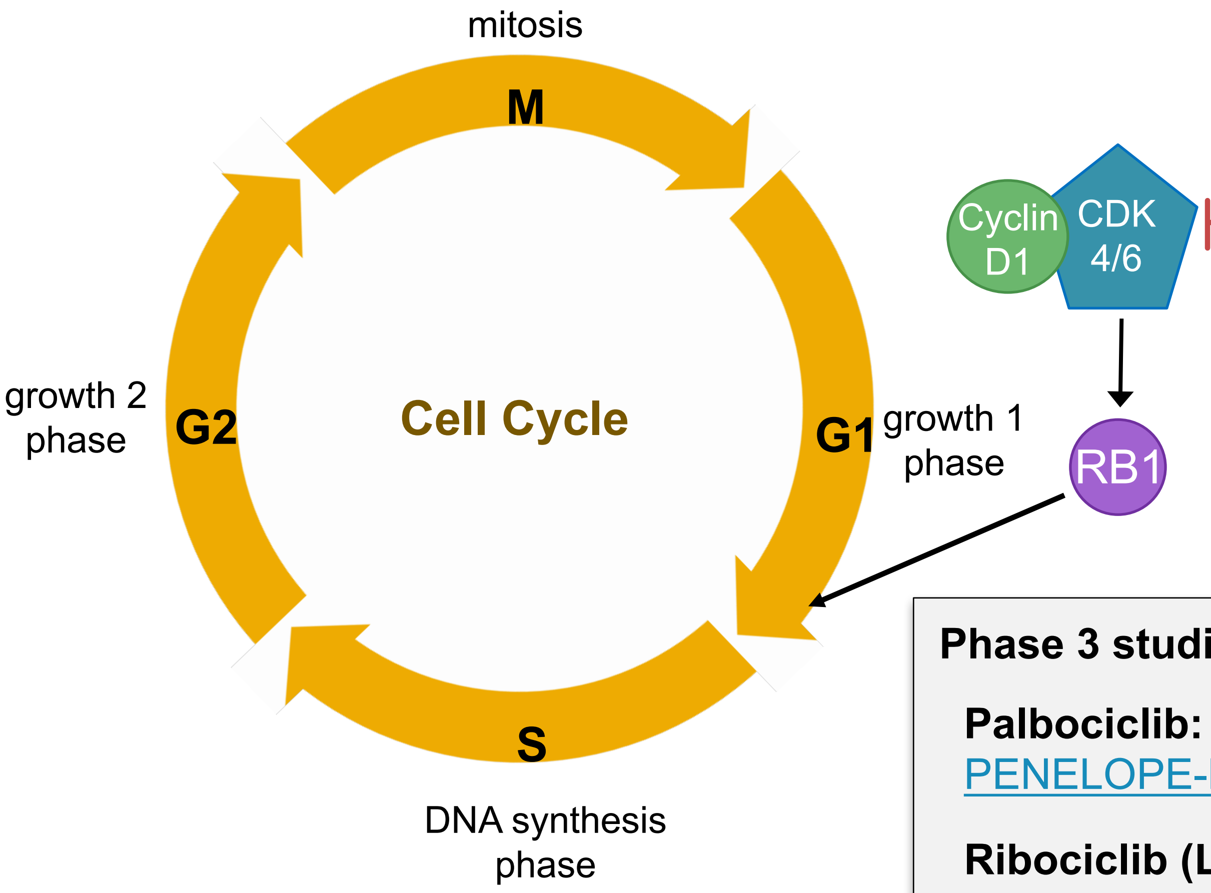

CDK 4/6 inhibitors:
- Palbociclib
- Ribociclib (LEE011)
- Abemaciclib

Phase 3 studies of CDK4/6 inhibitors

Palbociclib: PALOMA-2; PALOMA-3; PEARL; PENELOPE-B

Ribociclib (LEE011): MONALEESA-2

Abemaciclib: MONARCH 2; MONARCH 3

Current on 7/1/2015. © 2015 MyCancerGenome.org

CDK 4/6 Inhibitor Palbociclib

1st line therapy → 2nd line therapy or greater → 3rd line therapy

Trial	Treatment	Result
NCT01320592 Phase 1 (3rd line therapy)	palbociclib + paclitaxel (n = 15)	Safe, well tolerated, and some prolonged tumor responses (Clark et al. 2014)

Current on 7/1/2015. © 2015 MyCancerGenome.org

CDK 4/6 Inhibitor Palbociclib

PALOMA-1
NCT00721409
Phase 2

palbociclib + letrozole
(*n* = 84)

Improved median progression-free survival (PFS) compared to letrozole alone (Finn et al. 2015)
PFS: 20.2 months
Overall survival: 37.5 months

letrozole (*n* = 81)

PFS: 10.2 months

NCT01037790
Phase 2

Palbociclib
(*n* = 37)

Clinical benefit rate: 29%
Median PFS: 5 months
(DeMichele et al. 2014)

1st line therapy → 2nd line therapy or greater → 3rd line therapy

NCT01320592
Phase 1

palbociclib + paclitaxel
(*n* = 15)

Safe, well tolerated, and some prolonged tumor responses
(Clark et al. 2014)

Current on 7/1/2015. © 2015 MyCancerGenome.org

CDK 4/6 Inhibitor Palbociclib

Improved median progression-free survival (PFS) compared to letrozole alone (Finn et al. 2015)
PFS: 20.2 months
Overall survival: 37.5 months

PALOMA-1
NCT00721409
Phase 2

palbociclib + letrozole (*n* = 84)

letrozole (*n* = 81)

PFS: 10.2 months

Improved progression-free survival compared to placebo + fulvestrant (Turner et al. 2015);
PFS: 9.2 months

PALOMA-3
NCT01942135
Phase 3

palbociclib + fulvestrant (n = 347)

placebo + fulvestrant (n = 174)

PFS: 3.8 months

Clinical benefit rate: 29%
Median PFS: 5 months
(DeMichele et al. 2014)

NCT01037790
Phase 2

Palbociclib (*n* = 37)

1st line therapy → 2nd line therapy or greater → 3rd line therapy

NCT01320592
Phase 1

palbociclib + paclitaxel (*n* = 15)

Safe, well tolerated, and some prolonged tumor responses (Clark et al. 2014)

Palbociclib + letrozole was granted accelerated FDA approval on Feb. 3, 2015

- for 1st line or initial endocrine therapy (Phase 2 trial, PALOMA-1)
- in postmenopausal women with ER+/HER2-negative advanced breast cancer
- Phase 3 trial (PALOMA-2) of this combination is ongoing

Current on 7/1/2015. © 2015 MyCancerGenome.org

CDK 4/6 Inhibitor Palbociclib: Ongoing Trials

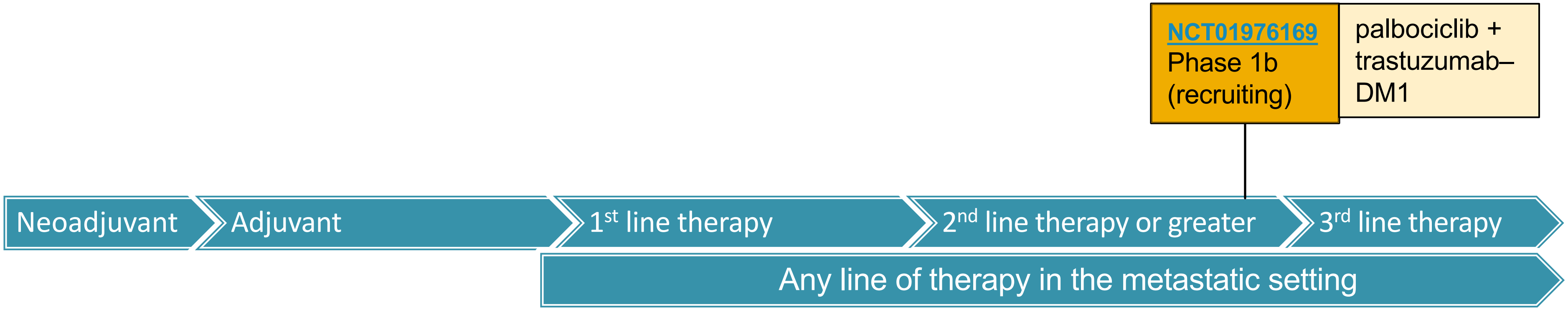

Current on 7/1/2015. © 2015 MyCancerGenome.org

CDK 4/6 Inhibitor Palbociclib: Ongoing Trials

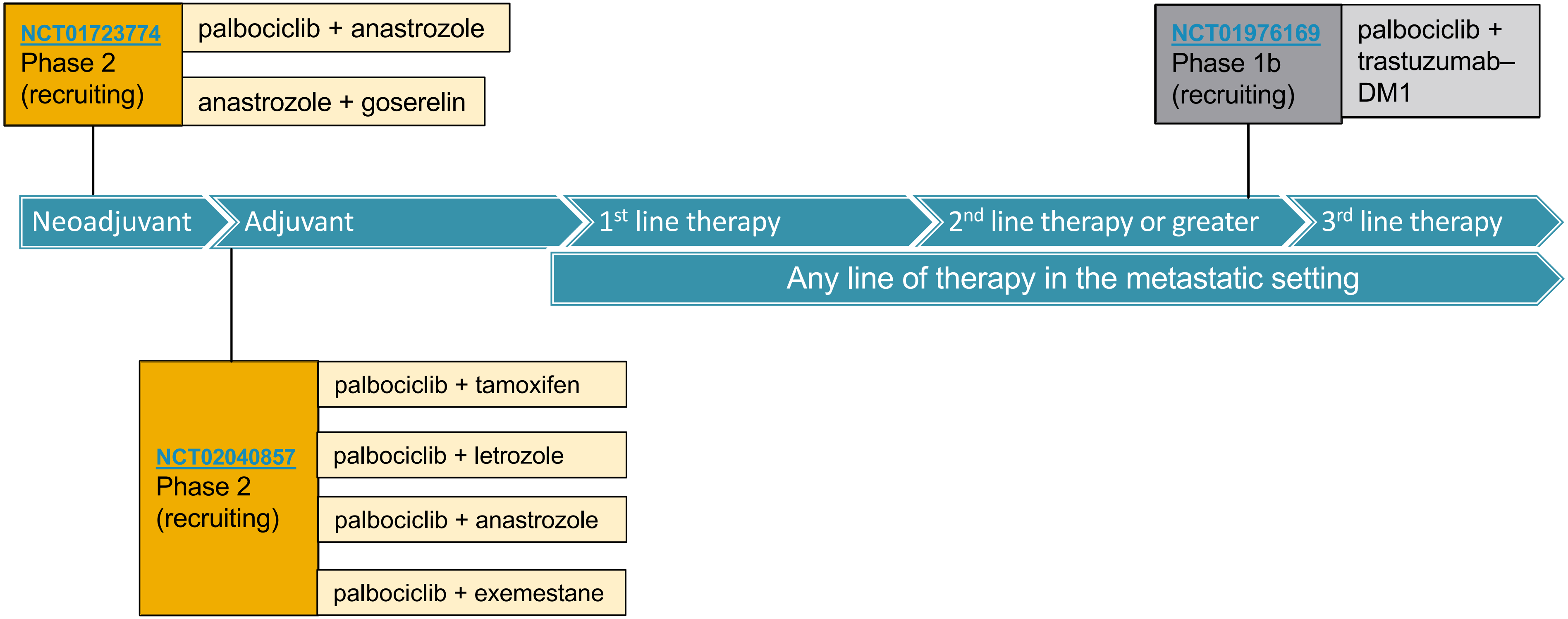

Current on 7/1/2015. © 2015 MyCancerGenome.org

CDK 4/6 Inhibitor Palbociclib: Ongoing Trials

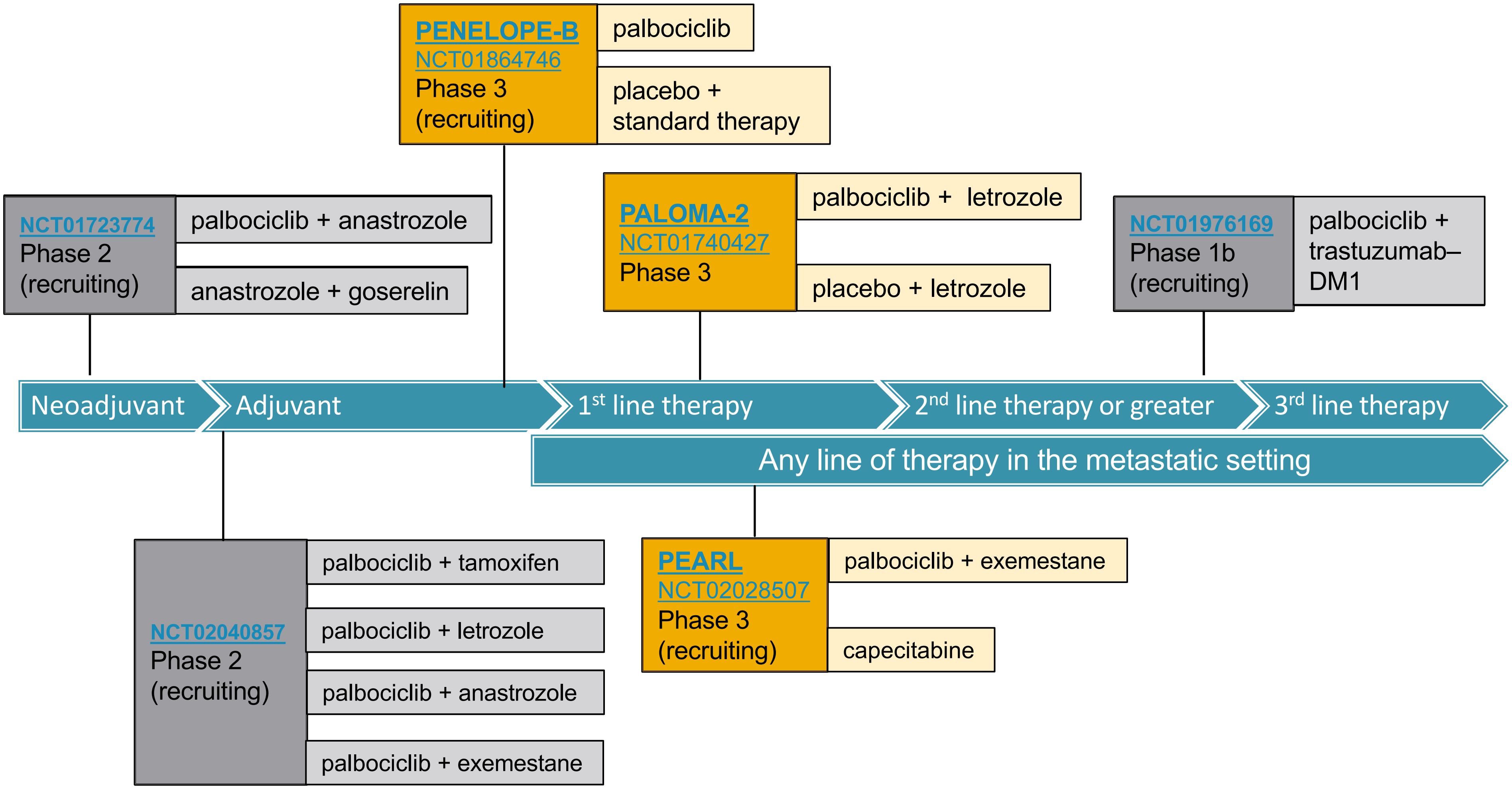

Current on 7/1/2015. © 2015 MyCancerGenome.org

CDK 4/6 Inhibitor Ribociclib (LEE011)

1st line therapy → 2nd line therapy or greater → 3rd line therapy

These drug combinations had acceptable safety profiles and a 67% clinical benefit rate (Munster et al. 2014).

NCT01872260
Phase 1b
(recruiting)

- LEE011+ letrozole (*n* = 6)
- BYL719 + letrozole (*n* = 5)

Current on 7/1/2015. © 2015 MyCancerGenome.org

CDK 4/6 Inhibitor Ribociclib (LEE011)

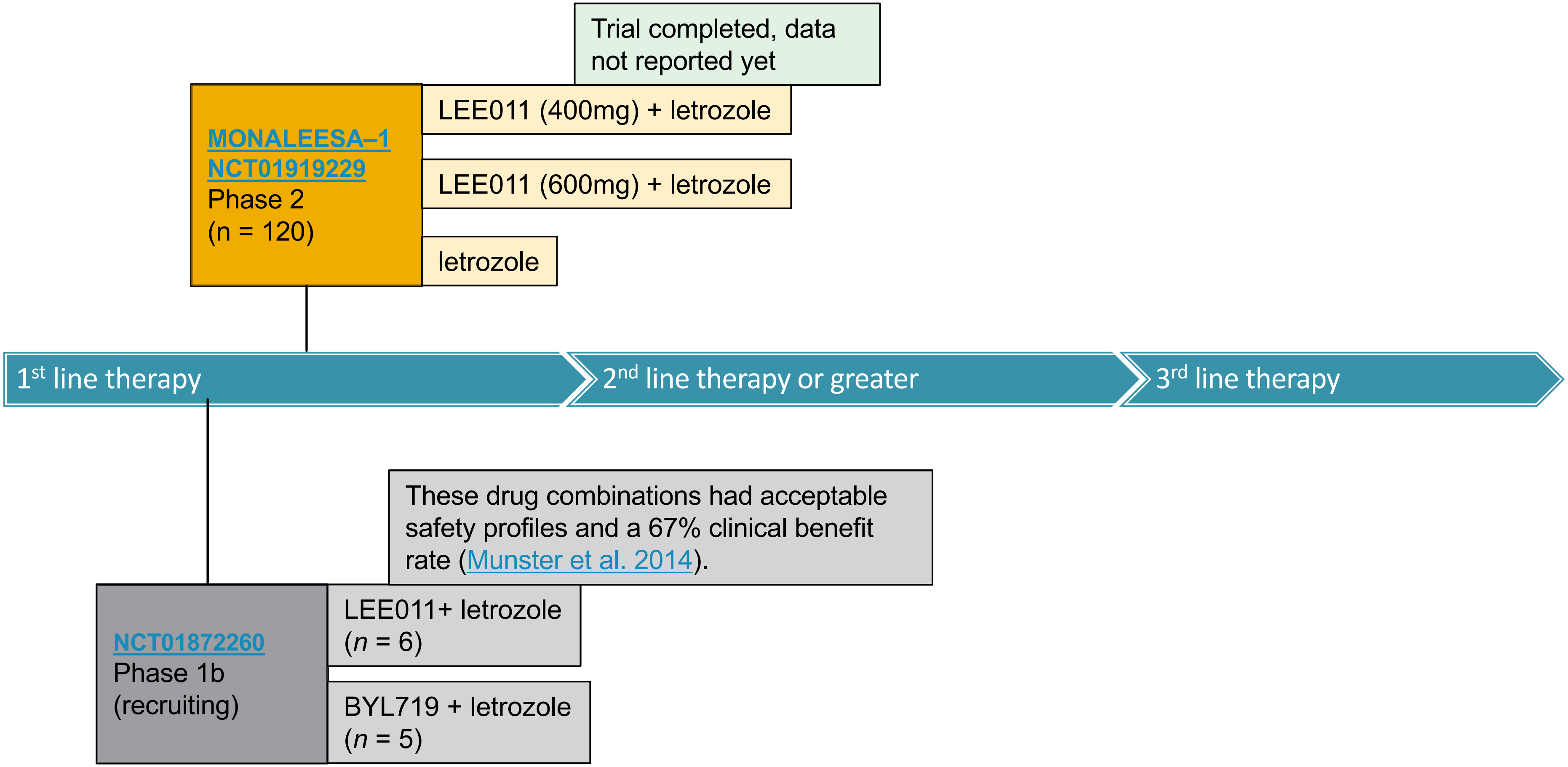

Current on 7/1/2015. © 2015 MyCancerGenome.org

CDK 4/6 Inhibitor Ribociclib (LEE011): Ongoing Trials

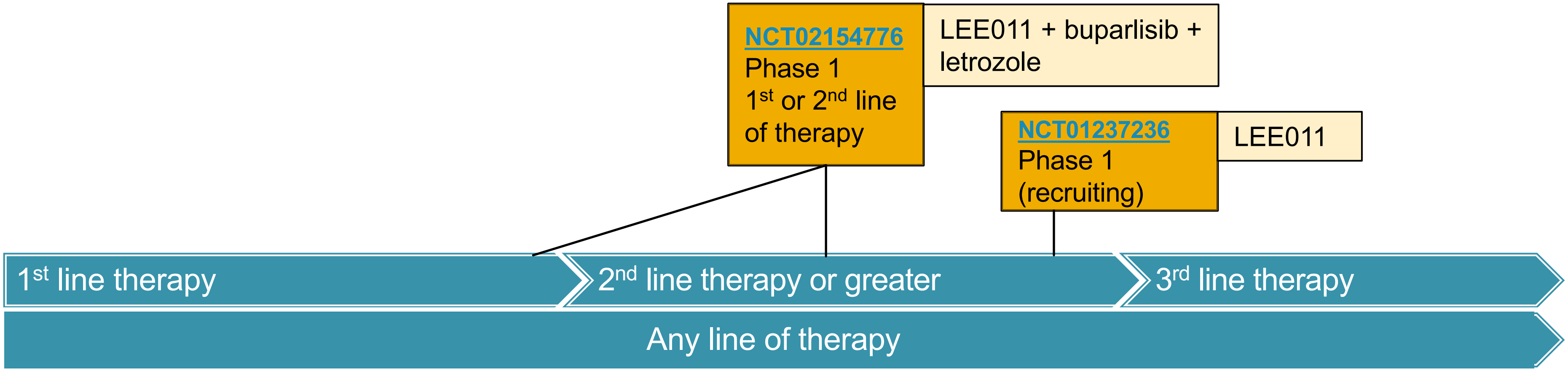

Current on 7/1/2015. © 2015 MyCancerGenome.org

CDK 4/6 Inhibitor Ribociclib (LEE011): Ongoing Trials

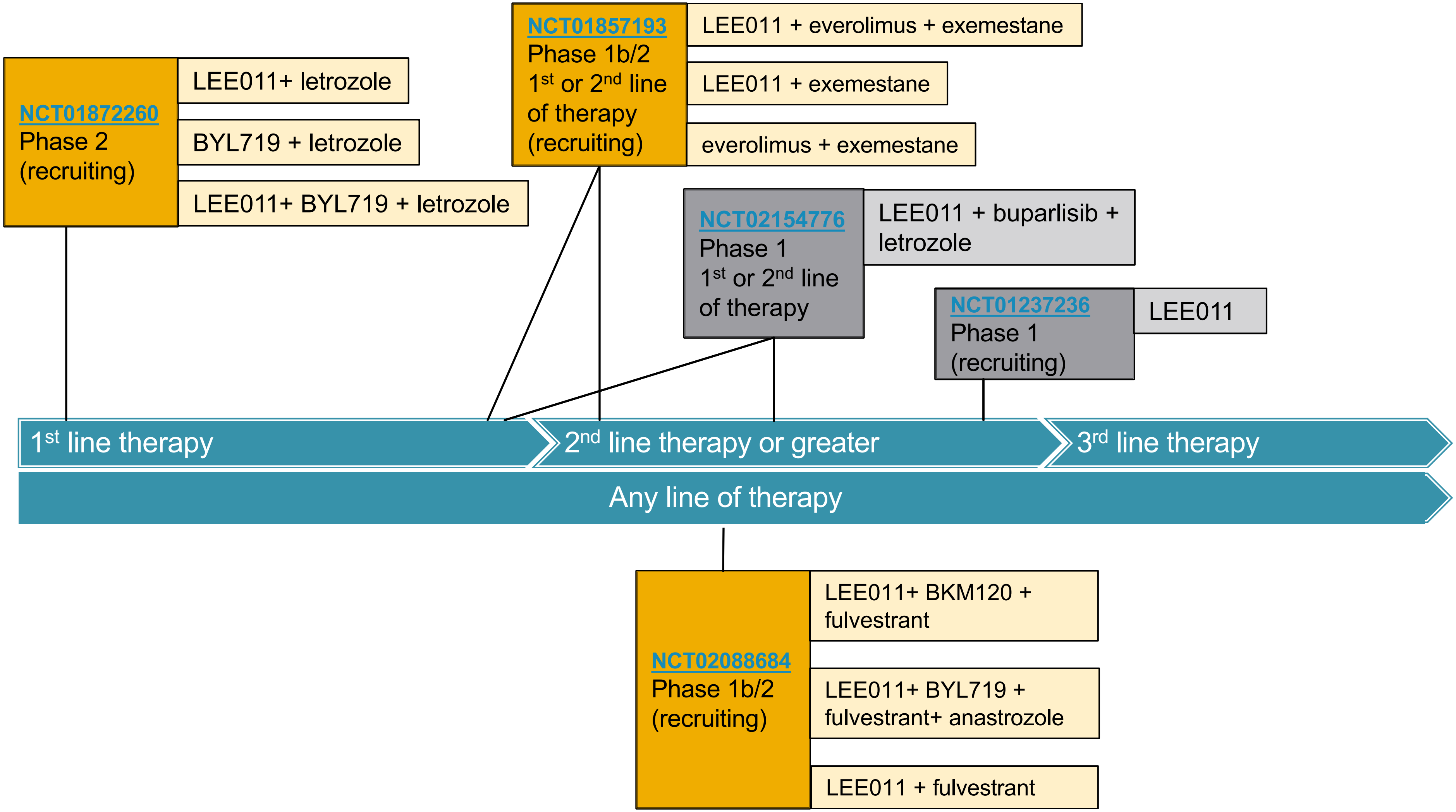

Current on 7/1/2015. © 2015 MyCancerGenome.org

CDK 4/6 Inhibitor Ribociclib (LEE011): Ongoing Trials

NCT01872260
Phase 2
(recruiting)
- LEE011+ letrozole
- BYL719 + letrozole
- LEE011+ BYL719 + letrozole

MONALEESA – 2
NCT01958021
Phase 3
(recruiting)
- LEE011+ letrozole
- Letrozole + placebo

NCT01857193
Phase 1b/2
1st or 2nd line of therapy
(recruiting)
- LEE011 + everolimus + exemestane
- LEE011 + exemestane
- everolimus + exemestane

NCT02154776
Phase 1
1st or 2nd line of therapy
- LEE011 + buparlisib + letrozole

NCT01237236
Phase 1
(recruiting)
- LEE011

1st line therapy → 2nd line therapy or greater → 3rd line therapy

Any line of therapy

NCT02088684
Phase 1b/2
(recruiting)
- LEE011+ BKM120 + fulvestrant
- LEE011+ BYL719 + fulvestrant+ anastrozole
- LEE011 + fulvestrant

Current on 7/1/2015. © 2015 MyCancerGenome.org

CDK 4/6 Inhibitor Abemaciclib

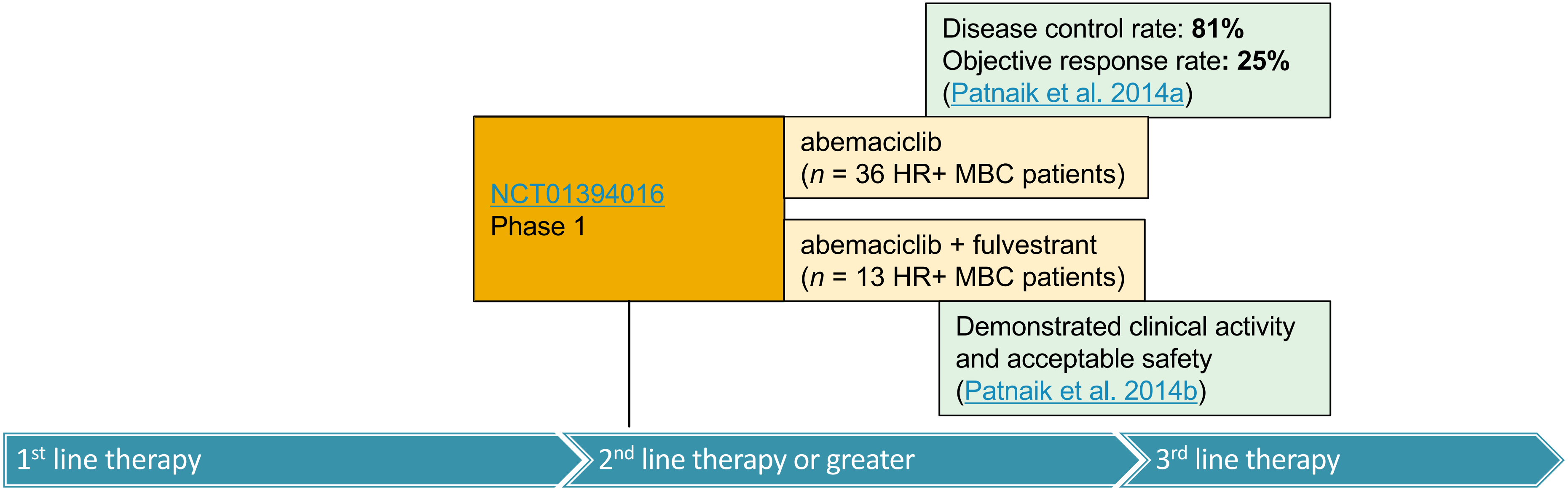

Current on 7/1/2015. © 2015 MyCancerGenome.org

CDK 4/6 Inhibitor Abemaciclib: Ongoing Trials

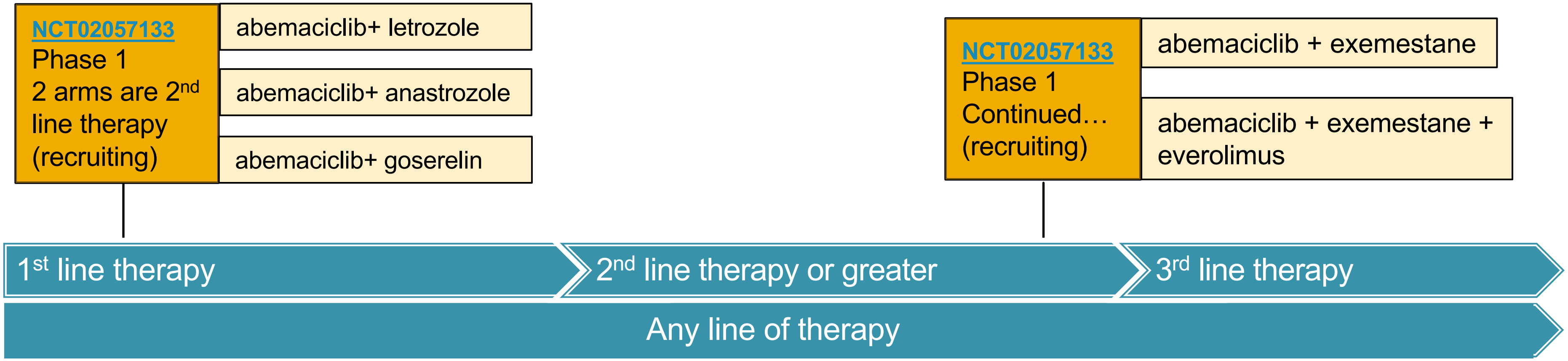

Current on 7/1/2015. © 2015 MyCancerGenome.org

CDK 4/6 Inhibitor Abemaciclib: Ongoing Trials

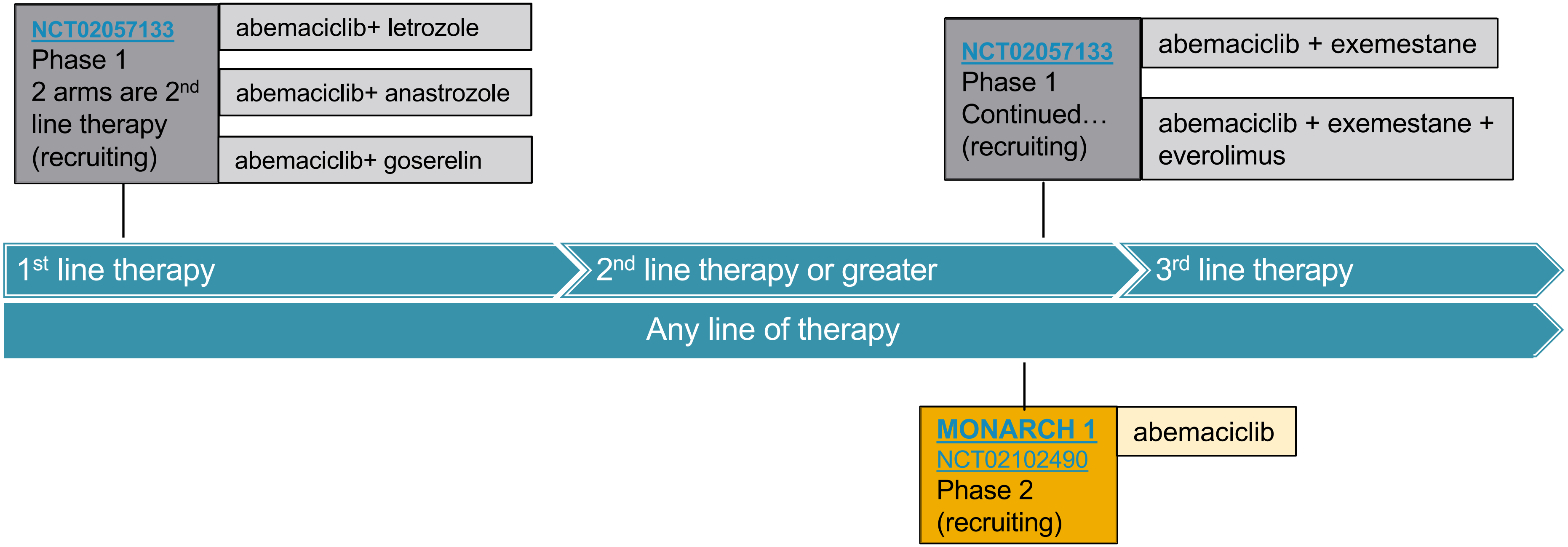

Current on 7/1/2015. © 2015 MyCancerGenome.org

CDK 4/6 Inhibitor Abemaciclib: Ongoing Trials

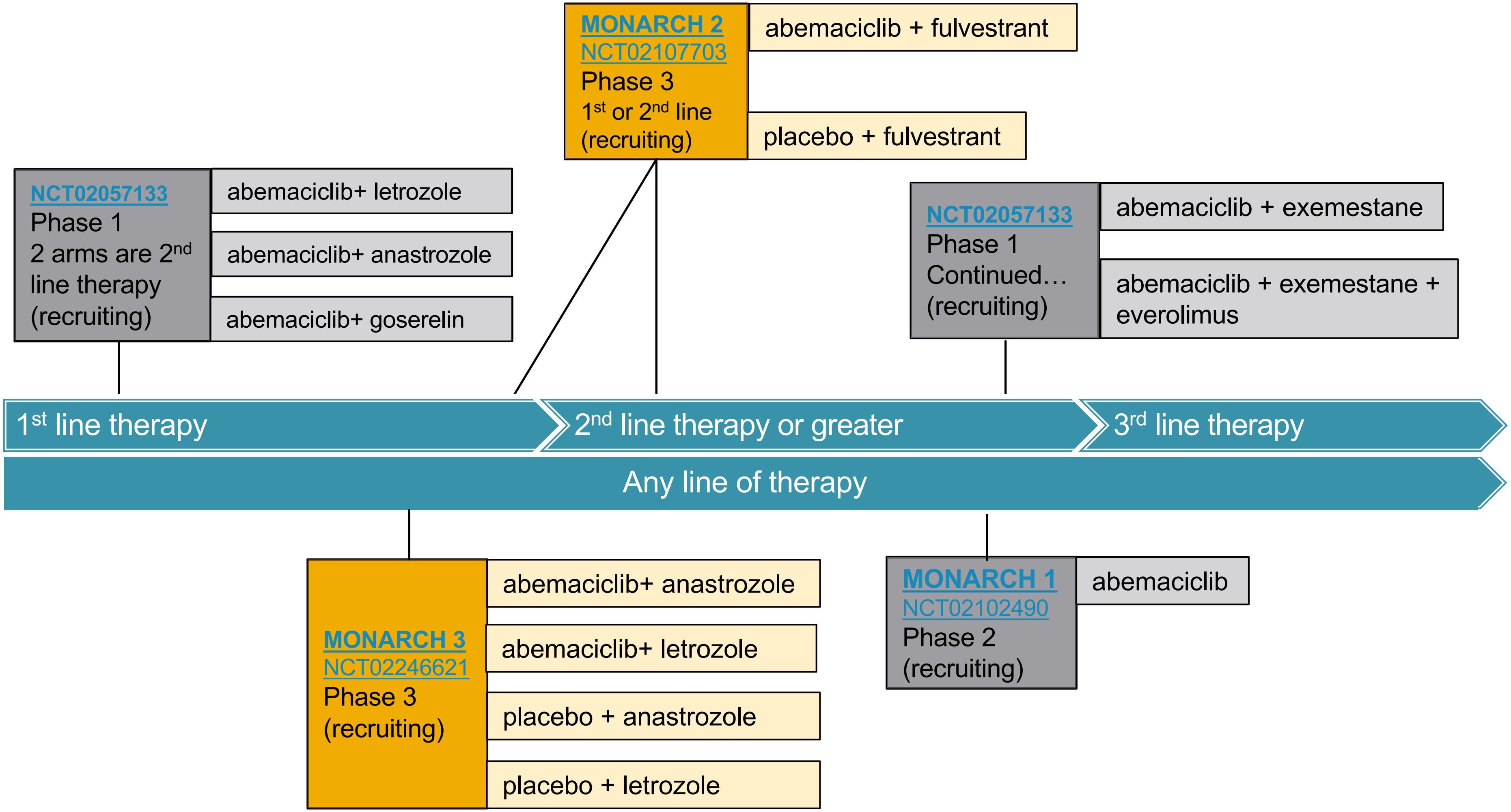

Current on 7/1/2015. © 2015 MyCancerGenome.org

Summary

- CDK4/6 inhibitors are a promising therapy for ER+ metastatic breast cancer
- Palbociclib plus letrozole has been granted accelerated FDA approved for 1st line or initial treatment in postmenopausal women with ER+ HER2-negative advanced breast cancer
- Ongoing trials are evaluating safety and efficacy of CDK4/6 inhibitors alone and in combination with other therapies

Current on 7/1/2015. © 2015 MyCancerGenome.org

References

- Allred DC, Carlson RW, Berry DA, Burstein HJ, Edge SB, Goldstein LJ, Gown A, Hammond ME, Iglehart JD, Moench S, Pierce LJ, Ravdin P, Schnitt SJ, Wolff AC. “NCCN Task Force Report: Estrogen Receptor and Progesterone Receptor Testing in Breast Cancer by Immunohistochemistry.” *J Natl Compr Canc Netw*. Suppl 6, no.S1-S21; (Sep 2009) quiz S22-3. PubMed PMID: 19755043.
- Bachelot, T., C. Bourgier, C. Cropet, I. Ray-Coquard, J. M. Ferrero, G. Freyer, S. Abadie-Lacourtoisie, *et al.* "Randomized Phase Ii Trial of Everolimus in Combination with Tamoxifen in Patients with Hormone Receptor-Positive, Human Epidermal Growth Factor Receptor 2-Negative Metastatic Breast Cancer with Prior Exposure to Aromatase Inhibitors: A Gineco Study." [In eng]. *J Clin Oncol* 30, no. 22 (Aug 1 2012): 2718-24. PubMed PMID: 22565002.
- Baselga J, Campone M, Piccart M, Burris HA 3rd, Rugo HS, Sahmoud T, Noguchi S, Gnant M, Pritchard KI, Lebrun F, Beck JT, Ito Y, Yardley D, Deleu I, Perez A, Bachelot T, Vittori L, Xu Z, Mukhopadhyay P, Lebwohl D, Hortobagyi GN. Everolimus in postmenopausal hormone-receptor-positive advanced breast cancer. N Engl J Med. 2012 Feb 9;366(6):520-9. PubMed PMID: 22149876.
- Butt AJ, McNeil CM, Musgrove EA, Sutherland RL. Downstream targets of growth factor and oestrogen signalling and endocrine resistance: the potential roles of c-Myc, cyclin D1 and cyclin E. Endocr Relat Cancer. 2005 Jul;12 Suppl 1:S47-59. Review. PubMed PMID: 16113099.
- Cancer Genome Atlas Network. “Comprehensive molecular portraits of human breast tumours.” *Nature* 490, no.7418 (Oct 2012):61-70. doi: 10.1038/nature11412. PubMed PMID: 23000897.
- Cerami E, Gao J, Dogrusoz U, Gross BE, Sumer SO, Aksoy BA, Jacobsen A, Byrne CJ, Heuer ML, Larsson E, Antipin Y, Reva B, Goldberg AP, Sander C, Schultz N. “The cBio cancer genomics portal: an open platform for exploring multidimensional cancer genomics data.” *Cancer Discov* 2, no.10 (May 2012):401-4. doi: 10.1158/2159-8290.CD-12-0095. Erratum in: *Cancer Discov*. 2012 Oct;2(10):960. PubMed PMID: 22588877.
- Clark, A. S., P. J. O'Dwyer, D. Heitjan, P. Lal, M. D. Feldman, M. Gallagher, C. Redlinger, *et al.* "A Phase I Trial of Palbociclib and Paclitaxel in Metastatic Breast Cancer." *J Clin Oncol* 32, no. 5s (2014): abstr 527.
- Colditz GA, Rosner BA, Chen WY, Holmes MD, Hankinson SE. “Risk factors for breast cancer according to estrogen and progesterone receptor status.” *J Natl Cancer Inst*. 96, no.3 (Feb 2004): 218-28. PubMed PMID: 14759989.
- DeMichele A, Clark As, Tan KS, Heitjan DF, Gramlich K, Gallagher M, Lal P, et al. “ CDK 4/6 Inhibitor Pabociclib (PD0332991) in Rb+ Advanced Breast Cancer: Pahse II Activity, Safety, and Predictive Biomarker Assessment.” *Clin Cancer Res* 21, no. 5 (Mar 2015): 995-1001. PubMed PMID: 25501126.
- Finn, R. S., J. P. Crown, I. Lang, K. Boer, I. M. Bondarenko, S. O. Kulyk, J. Ettl, *et al.* "The cyclin-dependent kinase 4/6 inhibitor palbociclib in combination with letrozole versus letrozole alone as first-line treatment of oestrogen receptor-positive, HER2-negative, advanced breast cancer (PALOMA-1/TRIO-18): a randomised phase 2 study." *Lancet Oncol* S1470-2045, no.14 (Jan 2015): 71159-3. PubMed PMID: 25524798.
- Gao, J., B. A. Aksoy, U. Dogrusoz, G. Dresdner, B. Gross, S. O. Sumer, Y. Sun, *et al.* "Integrative Analysis of Complex Cancer Genomics and Clinical Profiles Using the Cbioportal." *Sci Signal* 6, no. 269 (Apr 2 2013): pl1. PubMed PMID: 23550210.
- Giuliano M, Schifp R, Osborne CK, Trivedi MV. Biological mechanisms and clinical implications of endocrine resistance in breast cancer. Breast. 2011 Oct;20 Suppl 3:S42-9. doi: 10.1016/S0960-9776(11)70293-4. Review. PubMed PMID: 22015292.

Current on 7/1/2015. © 2015 MyCancerGenome.org

References Continued…

- Infante, J. R., G. Shapiro, P. Witteveen, J. F. Gerecitano, V. Ribrag, R. Chugh, I. Issa, et al. "A Phase I Study of the Single-Agent Cdk4/6 Inhibitor Lee011 in Pts with Advanced Solid Tumors and Lymphomas.". J Clin Oncol 32, no. 15_suppl (2014): abstr 2528.
- Jiang Z, Jones R, Liu JC, Deng T, Robinson T, Chung PE, Wang S, Herschkowitz JI, Egan SE, Perou CM, Zacksenhaus E. “RB1 and p53 at the crossroad of EMT and triple-negative breast cancer.” *Cell Cycle* 10, no.10 (May 2011):1563-70. Review. PubMed PMID: 21502814.
- Migliaccio I, Di Leo A, Malorni L. Cyclin-dependent kinase 4/6 inhibitors in breast cancer therapy. Curr Opin Oncol. 2014 Nov;26(6):568-75. PubMed PMID: 25188473.
- Miller TW, Hennessy BT, González-Angulo AM, Fox EM, Mills GB, Chen H, Higham C, García-Echeverría C, Shyr Y, Arteaga CL. “Hyperactivation of phosphatidylinositol-3 kinase promotes escape from hormone dependence in estrogen receptor-positive human breast cancer.” *J Clin Invest* 120, no.7 (Jul 2010):2406-13. doi: 10.1172/JCI41680. PubMed PMID: 20530877.
- Miller TW, Balko JM, Arteaga CL. “Phosphatidylinositol 3-kinase and antiestrogen resistance in breast cancer.” *J Clin Oncol* 29, no. 33 (Nov 2011a):4452-61. doi: 10.1200/JCO.2010.34.4879. Review. PubMed PMID: 22010023.
- Munster PN, Hamilton EP, Estevez LG, De Boer RH, Mayer IA, Campone M, Asano S, et al. “Phase IB study of LEE011 and BYL719 in combination with letrozole in ER+, HER2- breast cancer.” *J Clin Oncol* 32, no.26_suppl (2014): abstr 143.
- Osborne CK, Schiff R. Mechanisms of endocrine resistance in breast cancer. Annu Rev Med. 2011;62:233-47. doi: 10.1146/annurev-med-070909-182917. Review. PubMed PMID: 20887199.
- Patnaik, A., L. S. Rosen, S. M. Tolaney, A. W. Tolcher, J. W. Goldman, L. Gandhi, K. P. Papadopoulos, et al. "Clinical Activity of Ly2835219, a Novel Cell Cycle Inhibitor Selective for Cdk4 and Cdk6, in Patients with Metastatic Breast Cancer." Cancer Res, no. 74 (2014a): CT232.
- Patnaik, A., L. S. Rosen, S. M. Tolaney, A. W. Tolcher, J. W. Goldman, L. Gandhi, K. P. Papadopoulos, et al. "Ly2835219, a Novel Cell Cycle Inhibitor Selective for Cdk4/6, in Combination with Fulvestrant for Patients with Hormone Receptor Positive (Hr+) Metastatic Breast Cancer." J Clin Oncol 32, no. 5s (2014b): abstr 534.
- Piccart M, Hortobagyi GN, Campone M, Pritchard KI, Lebrun F, Ito Y, Noguchi S, Perez A, Rugo HS, Deleu I, Burris HA 3rd, Provencher L, Neven P, Gnant M, Shtivelband M, Wu C, Fan J, Feng W, Taran T, Baselga J. “Everolimus plus exemestane for hormone-receptor-positive, human epidermal growth factor receptor-2-negative advanced breast cancer: overall survival results from BOLERO-2†.” *Ann Oncol* 25, no. 12 (Dec 2014):2357-62. doi: 10.1093/annonc/mdu456. PubMed PMID: 25231953.
- Ring A, Dowsett M. “Mechanisms of tamoxifen resistance.” *Endocr Relat Cancer* 11, no. 4. (Dec 2004):643-58. Review. PubMed PMID: 15613444.
- Turner NC, Ro J, André F, Loi S, Verma S, Iwata H, Harbeck N, Loibl S, Huang Bartlett C, Zhang K, Giorgetti C, Randolph S, Koehler M, Cristofanilli M. Palbociclib in Hormone-Receptor-Positive Advanced Breast Cancer. *N Engl J Med.* 2015 Jul 16;373(3):209-19. doi: 10.1056/NEJMoa1505270. Epub 2015 Jun 1. PubMed PMID: 26030518.
- Yardley DA, Noguchi S, Pritchard KI, Burris HA 3rd, Baselga J, Gnant M, Hortobagyi GN, Campone M, Pistilli B, Piccart M, Melichar B, Petrakova K, Arena FP, Erdkamp F, Harb WA, Feng W, Cahana A, Taran T, Lebwohl D, Rugo HS. “Everolimus plus exemestane in postmenopausal patients with HR(+) breast cancer: BOLERO-2 final progression-free survival analysis. “ *Adv Ther* 30, no. 10 (Oct 2013):870-84. doi: 10.1007/s12325-013-0060-1. Erratum in: *Adv Ther*. 2014 Sep;31(9):1008-9. PubMed PMID: 24158787

Current on 7/1/2015. © 2015 MyCancerGenome.org

Audio Script (version 2)

Slide 2
Thank you for participating in this study, which has been prepared by My Cancer Genome at Vanderbilt University. This study was funded by Pfizer Independent Grants for Learning and Change. In this presentation, we will look at strategies for overcoming resistance to endocrine therapy in estrogen receptor positive breast cancer, focusing on CDK4/6 inhibitors.

Slide 3
As we show in this pictogram, up to 70% of breast cancers express the estrogen receptor, which is referred to as estrogen-receptor-positive breast cancer.

Slide 4
Estrogen is a steroid hormone that controls cellular processes such as cell division, growth, differentiation, and proliferation in target tissues such as breast and ovaries.

In order to understand how drugs can target aberrant estrogen signaling in breast cancer, we will briefly review the different parts of the signaling process.

Slide 5
In normal estrogen receptor signaling, the aromatase enzyme converts androgen into estrogen. Estrogen acts as a ligand and binds to the estrogen receptor.

Slide 6
This activates the signaling pathways that regulate cell growth processes, including those related to sexual development and reproductive function. Aberrant estrogen signaling can drive cell growth in several types of cancer, including breast, ovarian, endometrial, and prostate cancer.

Slide 7
There are three classes of endocrine agents, which are used to treat estrogen-receptor-positive breast cancer:

Slide 8
First, steroidal and nonsteroidal aromatase inhibitors, such as anastrozole, letrozole, and exemestane, inhibit the enzyme aromatase, which prevents the synthesis of estrogen. These agents are used in postmenopausal women in adjuvant and metastatic settings;

Slide 9
Second, selective estrogen receptor modulators, or SERMs, such as tamoxifen, compete with estrogen as a ligand for the estrogen receptor, inhibiting the pathway. These agents are used in premenopausal and postmenopausal women in adjuvant and metastatic settings;

Slide 10
Third, selective estrogen receptor degraders or downregulators, or SERDs, such as fulvestrant, bind to the estrogen receptor and cause it to change shape, which inhibits the receptor’s ability to bind estrogen and sometimes downregulates the estrogen receptor itself. These agents are used in postmenopausal women in the metastatic setting.

Despite the presence of estrogen receptor expression in breast cancer tissues, not all estrogen-receptor-positive breast cancers respond to endocrine therapy. Molecular alterations in estrogen-receptor-positive breast cancers can lead to primary or acquired resistance to endocrine therapy.

Slide 11
Primary resistance is defined as recurrence, either within adjuvant therapy or within 6 to 12 months of completion of adjuvant therapy or as disease progression less than six months after treatment.

Slide 12
Primary resistance may be a result of

- FGFR amplification,
- Loss of ER-alpha,
- Post-translational modification of ER-alpha,
- Estrogen receptor mutations,
- Expression of estrogen receptor coactivation or corepression factors,
- MYC amplification or overexpression, or
- Cyclin D1 amplification or expression.

Slide 13
Acquired resistance is defined as recurrence at least 6 to 12 months after completion of adjuvant therapy or as disease progression more than 6 months after endocrine therapy was initiated in the metastatic setting.

Slide 14
Acquired resistance may be a result of

- Activation of growth factor signaling pathways such as
  - PI3K/AKT1/MTOR or
  - MAPK/ERK;
- Estrogen receptor mutations, or
- Changes in the tumor microenvironment.

In the next slides, we will discuss current approaches to addressing resistance to endocrine therapy.

Slide 15
In recent years, there has been an increase in the number of clinical trials using novel targeted therapeutic strategies in endocrine resistant ER+ breast cancer, with varying levels of evidence for improvement in outcomes.

Slide 16
We will briefly discuss mTOR inhibitors, and

Slide 17
then we will focus on CDK4/6 inhibitors for the remainder of the presentation.

Slide 18
The mTOR inhibitor everolimus in combination with the endocrine therapy exemestane is FDA-approved for estrogen-receptor-positive metastatic breast cancer resistant to letrozole or anastrozole. Approval of the drug combination was based on the results of the phase 3 trial BOLERO-2. In this study, 485 patients

received combination therapy. The study showed an improvement in progression free survival by 4.6 months; however, there was no change in overall survival.

Slide 19
Now we will discuss the use of cyclin-dependent kinase—also known as CDK4/6—inhibitors to overcome resistance to endocrine therapy. First, we will introduce the roles that cyclin D1 and CDK4/6 play in the cell. We will then discuss combining CDK4/6 inhibitors with hormonal agents and clinical trials of CDK4/6 inhibitors.

Slide 20
Expression of cyclin D1 is regulated by the estrogen receptor. As a result, overexpression or amplification of cyclin D1 can cause resistance to endocrine agents. In the next two slides, we will explore how this occurs.

Slide 21
Cyclin D1 is involved in regulating entry into the synthesis phase, or S phase, of the cell cycle.

Slide 22
It binds to cyclin-dependent kinases 4 and 6, which are collectively referred to as CDK4/6.

Slide 23
The cyclin D1–CDK4 or 6 complex phosphorylates the retinoblastoma tumor suppressor protein, also called RB1.

Slide 24
RB1 releases the transcription factors required for entry into the S phase of the cell cycle.

Slide 25
Secondary signals in the estrogen signaling pathway can become activated through cyclin D1 amplification and CDK4/6 gain of function. RB1 alterations causing loss of function also result in pathway activation.

Slide 26
CDK4/6 inhibition is under investigation as a therapeutic strategy in the adjuvant, neoadjuvant, and metastatic settings; as well as in both aromatase-inhibitor-naïve and -resistant settings.

Slide 27
In the subset of patients whose tumors demonstrate RB1 mutation or loss, CDK4/6 inhibitors may not be effective, because the RB1 alteration occurs downstream of that signaling node.

Slide 28
Combining estrogen-receptor-targeting agents, such as

Slide 29
Aromatase inhibitors, with agents that target downstream components of the estrogen receptor pathway, such as

Slide 30
CDK 4/6 inhibitors, may improve therapeutic benefit compared to standard of care.

Slide 31
Several cell cycle inhibitors have been developed that target CDK4/6. The CDK 4/6 inhibitors include palbociclib, ribociclib, and abemaciclib.

Slide 32
All three agents are being studied in randomized phase 3 clinical trials as single agents or in combination with endocrine therapies.

In the next six slides, we will summarize the current status of clinical trials for palbociclib, ribociclib, and abemaciclib.

Slide 33
Palbociclib is the first CDK4/6 inhibitor I will discuss; results of several clinical trials have been reported. For each trial, the name, if there is one, the phase, and the national clinical trial identifier are shown, along with the treatment arms, the number of patients in each arm, and a summary of trial results, if available.

One phase one and

Slide 34
two phase two trials of palbociclib, either alone or in combination with letrozole, showed clinical benefit. Based on these trials,

Slide 35
the FDA granted accelerated approval to palbociclib for use in combination with letrozole for the treatment of postmenopausal women with HER2-negative hormone receptor positive breast cancer.

Slide 36
On April 15, 2015, the PALOMA–3 phase three trial of palbociclib plus fulvestrant was stopped after the interim analysis showed efficacy in metastatic estrogen-receptor positive, HER2 negative breast cancer “following disease progression during or after endocrine therapy”.

Slide 37
There are several ongoing phase one,

Slide 38
two, and

Slide 39
three clinical trials evaluating palbociclib in combination with other endocrine therapies as well as anti-HER2 therapies. These trials cover the neoadjuvant and adjuvant setting as well as the first, second, and third line therapies in the metastatic setting.

Slide 40
Ribociclib is the second CDK4/6 inhibitor we will discuss; results of several phase 1 clinical trials have been reported. A phase 1b trial of ribociclib in combination with letrozole demonstrated the acceptable drug safety profiles and a 67% clinical benefit rate.

Slide 41
A phase 2 trial, called MONALEESA-1, is closed to accrual, but the data are not yet reported.

Slide 42
There are several ongoing phase one,

Slide 43
two, and

Slide 44
three clinical trials evaluating ribociclib in combination with other endocrine therapies as well as anti-HER2 therapies. These trials cover first, second, and greater line therapies in the metastatic setting.

Slide 45
Abemaciclib is the third CDK4/6 inhibitor we will discuss; it is under evaluation for use in estrogen-receptor-positive metastatic breast cancer. In a phase one trial, abemaciclib alone and abemaciclib in combination with fulvestrant both showed clinical benefit.

Slide 46
There are several recruiting and ongoing phase one,

Slide 47
two, and

Slide 48
three clinical trials evaluating abemaciclib in combination with endocrine therapies and anti-HER2 therapies. These trials cover the first, second, and third line therapies in the metastatic setting.

Slide 49
In conclusion, CDK4/6 inhibitors are a promising therapy for estrogen-receptor-positive metastatic breast cancer.

The FDA has granted accelerated approval to palbociclinb for use in combination with letrozole for first-line or initial treatment in postmenopausal women with ER+/HER2-negative advanced breast cancer.

Ongoing trials are evaluating safety and efficacy of CDK4/6 inhibitors alone and in combination with other therapies.

Text (version 2)

Slide 2

Thank you for participating in this study, which has been prepared by My Cancer Genome at Vanderbilt University. This study was funded by Pfizer Independent Grants for Learning and Change. In this presentation, we will look at strategies for overcoming resistance to endocrine therapy in estrogen receptor positive breast cancer, focusing on CDK4/6 inhibitors.

Slide 3

As we show in this pictogram, up to 70% of breast cancers express the estrogen receptor, which is referred to as estrogen receptor positive breast cancer.

Slide 4

Estrogen is a steroid hormone that controls cellular processes such as cell division, growth, differentiation, and proliferation in target tissues such as breast and ovaries.

In order to understand how drugs can target aberrant estrogen signaling in breast cancer, we will briefly review the different parts of the signaling process.

Slide 5

In normal estrogen receptor signaling, the aromatase enzyme converts androgen into estrogen. Estrogen acts as a ligand and binds to the estrogen receptor.

Slide 6

This activates the signaling pathways that regulate cell growth processes, including those related to sexual development and reproductive function. Aberrant estrogen signaling can drive cell growth in several types of cancer, including breast, ovarian, endometrial, and prostate cancer.

Slides 7–10

There are three classes of endocrine agents, which are used to treat estrogen receptor positive breast cancer:

First, steroidal and nonsteroidal aromatase inhibitors, such as anastrozole, letrozole, and exemestane, inhibit the enzyme aromatase, which prevents the synthesis of estrogen. These agents are used in postmenopausal women in adjuvant and metastatic settings;

Second, selective estrogen receptor modulators, or SERMs, such as tamoxifen, compete with estrogen as a ligand for the estrogen receptor, inhibiting the pathway. These agents are used in premenopausal and postmenopausal women in adjuvant and metastatic settings;

Third, selective estrogen receptor degraders or downregulators, or SERDs, such as fulvestrant, bind to the estrogen receptor and cause it to change shape, which inhibits the receptor’s ability to bind estrogen and sometimes downregulates the estrogen receptor itself. These agents are used in postmenopausal women in the metastatic setting.

Despite the presence of estrogen receptor expression in breast cancer tissues, not all estrogen receptor positive breast cancers respond to endocrine therapy. Molecular alterations in estrogen receptor positive breast cancers can lead to primary or acquired resistance to endocrine therapy.

Slides 11–12

Primary resistance is defined as recurrence, either within adjuvant therapy or within 6 to 12 months of completion of adjuvant therapy or as disease progression less than six months after treatment.

Primary resistance may be a result of

- FGFR amplification,
- Loss of ERα,
- Post-translational modification of ERα,
- Estrogen receptor mutations,
- Expression of estrogen receptor coactivation or corepression factors,
- MYC amplification or overexpression, or
- Cyclin D1 amplification or expression.

Slides 13–14

Acquired resistance is defined as recurrence at least 6 to 12 months after completion of adjuvant therapy or as disease progression more than 6 months after endocrine therapy was initiated in the metastatic setting.

Acquired resistance may be a result of

- Activation of growth factor signaling pathways such as
    - PI3K/AKT1/MTOR or
    - MAPK/ERK;
- Estrogen receptor mutations, or
- Changes in the tumor microenvironment.

In the next slides, we will discuss current approaches to addressing resistance to endocrine therapy.

Slides 15–17

In recent years, there has been an increase in the number of clinical trials using novel targeted therapeutic strategies in endocrine resistant ER+ breast cancer, with varying levels of evidence for improvement in outcomes.

We will briefly discuss mTOR inhibitors, and then we will focus on CDK4/6 inhibitors for the remainder of the presentation.

Slide 18

The mTOR inhibitor everolimus in combination with the endocrine therapy exemestane is FDA-approved for estrogen receptor positive metastatic breast cancer resistant to letrozole or anastrozole. Approval of the drug combination was based on the results of the phase 3 trial BOLERO-2. In this study, 485 patients received combination therapy. The study showed an improvement in progression-free survival by 4.6 months; however, there was no change in overall survival.

Slide 19

Now we will discuss the use of cyclin-dependent kinase—also known as CDK4/6—inhibitors to overcome resistance to endocrine therapy. First, we will introduce the roles that cyclin D1 and CDK4/6 play in the cell. We will then discuss combining CDK4/6 inhibitors with hormonal agents and clinical trials of CDK4/6 inhibitors.

Slide 20
Expression of cyclin D1 is regulated by the estrogen receptor. As a result, overexpression or amplification of cyclin D1 can cause resistance to endocrine agents. In the next two slides, we will explore how this occurs.

Slides 21–24
Cyclin D1 is involved in regulating entry into the synthesis phase, or S phase, of the cell cycle.

It binds to cyclin-dependent kinases 4 and 6, which are collectively referred to as CDK4/6.

The cyclin D1–CDK4 or 6 complex phosphorylates the retinoblastoma tumor suppressor protein, also called RB1.

RB1 releases the transcription factors required for entry into the S phase of the cell cycle.

Slide 25
Secondary signals in the estrogen signaling pathway can become activated through cyclin D1 amplification and CDK4/6 gain of function. RB1 alterations causing loss of function also result in pathway activation.

Slide 26
CDK4/6 inhibition is under investigation as a therapeutic strategy in the adjuvant, neoadjuvant, and metastatic settings; as well as in both aromatase inhibitor-naïve and -resistant settings.

Slide 27
In the subset of patients whose tumors demonstrate RB1 mutation or loss, CDK4/6 inhibitors may not be effective, because the RB1 alteration occurs downstream of that signaling node.

Slides 28–30
Combining estrogen receptor-targeting agents, such as aromatase inhibitors, with agents that target downstream components of the estrogen receptor pathway, such as CDK 4/6 inhibitors, may improve therapeutic benefit compared to standard of care.

Slides 31–32
Several cell cycle inhibitors have been developed that target CDK4/6. The CDK 4/6 inhibitors include palbociclib, ribociclib, and abemaciclib.

All three agents are being studied in randomized phase 3 clinical trials as single agents or in combination with endocrine therapies.

In the next six slides, we will summarize the current status of clinical trials for palbociclib, ribociclib, and abemaciclib.

Slides 33–35
Palbociclib is the first CDK4/6 inhibitor I will discuss; results of several clinical trials have been reported. For each trial, the name, if there is one, the phase, and the national clinical trial identifier are shown, along with the treatment arms, the number of patients in each arm, and a summary of trial results, if available.

One phase 1 and two phase 2 trials of palbociclib, either alone or in combination with letrozole, showed clinical benefit. Based on these trials, the FDA granted accelerated approval to palbociclib for use in combination with letrozole for the treatment of postmenopausal women with HER2-negative hormone receptor positive breast cancer.

On April 15, 2015, the PALOMA–3 phase 3 trial of palbociclib plus fulvestrant was stopped after the interim analysis showed efficacy in metastatic estrogen-receptor positive, HER2 negative breast cancer "following disease progression during or after endocrine therapy."

Slides 36–38

There are several ongoing phase 1, 2, and 3 clinical trials evaluating palbociclib in combination with other endocrine therapies as well as anti-HER2 therapies. These trials cover the neoadjuvant and adjuvant settings as well as first, second, and third-line therapies in the metastatic setting.

Slides 39–40

Ribociclib is the second CDK4/6 inhibitor we will discuss; results of several phase 1 clinical trials have been reported. A phase 1b trial of ribociclib in combination with letrozole demonstrated the acceptable drug safety profiles and a 67% clinical benefit rate.

A phase 2 trial, called MONALEESA-1, is closed to accrual, but the data are not yet reported.

Slides 41–43

There are several ongoing phase 1, 2, and 3 clinical trials evaluating ribociclib in combination with other endocrine therapies as well as anti-HER2 therapies. These trials cover first, second, and greater line therapies in the metastatic setting.

Slide 44

Abemaciclib is the third CDK4/6 inhibitor we will discuss; it is under evaluation for use in estrogen receptor positive metastatic breast cancer. In a phase one trial, abemaciclib alone and abemaciclib in combination with fulvestrant both showed clinical benefit.

Slides 45–47

There are several recruiting and ongoing phase 1, 2, and 3 clinical trials evaluating abemaciclib in combination with endocrine therapies and anti-HER2 therapies. These trials cover the first, second, and third line therapies in the metastatic setting.

Slide 48

In conclusion,

- CDK4/6 inhibitors are a promising therapy for estrogen receptor positive metastatic breast cancer.
- The FDA has granted accelerated approval to palbociclib for use in combination with letrozole for first-line or initial treatment in postmenopausal women with ER+/HER2-negative advanced breast cancer.
- Ongoing trials are evaluating safety and efficacy of CDK4/6 inhibitors alone and in combination with other therapies.

Control Educational Materials

My Cancer Genome

MY CANCER GENOME®
GENETICALLY INFORMED CANCER MEDICINE

Search My Cancer Genome Go

Home | DIRECT | About Us

- Breast Cancer
- AKT1
- AR
- ERBB2
- ESR1
  - ER Expression
  - ESR1 Mutations Resistant to Antiestrogen Therapy
- FGFR1
- FGFR2
- PGR
- PIK3CA
- PTEN

- Other Diseases

- Molecular Medicine

- Take Our Survey
- Glossary
- News
- Our Team
- Acknowledgements

What is ESR1? | ESR1 in Breast Cancer | ER Expression | Clinical Trials

ER (ESR1)

Gene and Protein Description

Estrogen receptor 1 (ESR1; also known as ER) is a gene that encodes an estrogen receptor protein, estrogen receptor α (ERα). ESR1 is located on chromosome 6 (Gosden et al. 1986). Estrogen receptor β (ERβ) is a second estrogen receptor that plays a separate role in cancer biology and is encoded by a different gene (Thomas and Gustafsson 2011). The symbol ER generally refers to ERα. The protein functions in hormone binding. Estrogen receptors are important for sexual development and reproductive function. Missense mutations, nonsense mutations, silent mutations, frameshift deletions, and in-frame deletions are observed in cancers such as endometrial cancer, intestinal cancer, and stomach cancer.

Steroid Signaling Pathway

ER is a member of the steroid hormone signaling pathway, a cell signaling pathway that functions in transcriptional activation and gene expression. The pathway includes, but is not limited to, the following proteins: androgen receptor (AR), estrogen receptor 1 (ESR1), progesterone receptor (PGR), LRP1B, and TSHR. The steroid hormone signaling pathway may be activated by steroid hormones, such as estrogen and progesterone, which bind to a steroid binding protein.

Estrogen is a steroid hormone that controls cellular processes such as cell division, growth, differentiation, and proliferation. Estrogen is converted from androgen precursors by the aromatase enzyme. Aromatase converts androgens to estrogens. Estrogen acts as a ligand and binds to the estrogen receptor (ER), which results in changes in gene expression and the activation of signaling pathways that regulate cell growth processes, such as the cell cycle control signaling pathway.

Oncogenic Alterations in ESR1

ER Expression

- ER expression occurs in 73–75% of invasive breast cancers (Nadji et al. 2005; Rhodes et al. 2000).
- ER protein expression occurs in 40–76% of endometrial cancers (Merritt et al. 2010; Suthipintawong et al. 2008).

ESR1 Mutations

- ESR1 mutations are rare in primary breast cancers at the time of diagnosis (TCGA 2012).
- ESR1 mutations have been identified in up to 55% of ER-positive metastatic breast cancers that have been previously treated with antiestrogens in retrospective data sets (Jeselsohn et al. 2014; Merenbakh-Lamin et al. 2013; Robinson et al. 2013; Toy et al. 2013).
- Cases of ESR1 mutations in endometrial cancer have also been reported (TCGA 2013).

Related Pathways

- Hormone signaling

Contributors: Justin M. Balko, Pharm. D., Ph.D., Ingrid A. Mayer, M.D., M.S.C.I., Mia Levy, M.D., Ph.D., Carlos L. Arteaga, M.D.

Suggested Citation: Balko, J., I. Mayer, M. Levy, C. Arteaga. 2015. ER (ESR1). *My Cancer Genome* https://www.mycancergenome.org/content/disease/breast-cancer/esr1/?tab=0 (Updated December 7).

Last Updated: December 7, 2015

Disclaimer: The information presented at MyCancerGenome.org is compiled from sources believed to be reliable. Extensive efforts have been made to make this information as accurate and as up-to-date as possible. However, the accuracy and completeness of this information cannot be guaranteed. Despite our best efforts, this information may contain typographical errors and omissions. The contents are to be used only as a guide, and health care providers should employ sound clinical judgment in interpreting this information for individual patient care.

MCG Home | About Us | Acknowledgments | Give | Site Map | Legal | APIs and Licensing

My Cancer Genome is managed by the Vanderbilt-Ingram Cancer Center Copyright © 2010 - 2016 MY CANCER GENOME

MY CANCER GENOME®
GENETICALLY INFORMED CANCER MEDICINE

Search My Cancer Genome Go

Home DIRECT About Us

- Breast Cancer
- AKT1
- AR
- ERBB2
- ESR1
  - ER Expression
  - ESR1 Mutations Resistant to Antiestrogen Therapy
- FGFR1
- FGFR2
- PGR
- PIK3CA
- PTEN

- Other Diseases

- Molecular Medicine

- Take Our Survey
- Glossary
- News
- Our Team
- Acknowledgements

What is ESR1? | ESR1 in Breast Cancer | ER Expression | Clinical Trials

ER (ESR1) in Breast Cancer

Both ER expression and ESR1 mutations are observed in breast cancer. ER expression is common in primary breast cancers and occurs in 73–75% of invasive breast cancers (Nadji et al. 2005; Rhodes et al. 2000). ESR1 mutations are observed primarily in breast cancers that have developed resistance to antiestrogen therapy (Jeselsohn et al. 2014; Merenbakh-Lamin et al. 2013; Robinson et al. 2013; Toy et al. 2013).

ER-Positive Breast Cancer Sensitive to Endocrine Therapy

Endocrine therapies are a class of agents that target the estrogen receptor pathway. ER expression has been demonstrated to be predictive of benefit from tamoxifen, a member of the class of endocrine therapies known as selective estrogen receptor modulators (SERMs; Davies et al. 2012; EBCTCG 2011). Multi-year adjuvant treatment is currently the standard of care for early stage ER-positive breast cancer patients (NCCN 2013). For patients with ER-positive metastatic breast cancer, standard-of-care endocrine therapies include SERMs, estrogen agonists/antagonists, and aromatase inhibitors (NCCN 2013).

However, despite the demonstrated benefit, up to 50% of patients on first-line tamoxifen will eventually experience progressive disease (Nabholtz et al. 2000). In the first-line setting, a third of metastatic ER-positive breast cancer patients do not respond to aromatase inhibitors and the remainder will experience progression after an initial period of clinical response (Mouridsen et al. 2003; Nabholtz et al. 2000).

ER-Positive Breast Cancer Resistant to Endocrine Therapy

Resistance to endocrine therapy in ER-positive breast cancer is defined clinically as either primary or acquired (Bachelot et al. 2012).

Primary resistance is defined as

- Recurrence either within adjuvant therapy or within 6–12 months of completion of adjuvant therapy
- Disease progression < 6 months after treatment in the metastatic setting

Acquired resistance is defined as

- Recurrence at least 6–12 months after completion of adjuvant therapy
- Disease progression > 6 months after endocrine therapy initiated in the metastatic setting

A variety of mechanisms have been implicated in primary and acquired resistance to endocrine agents. Recently, there has been an increase in the number of clinical trials using novel targeted therapeutic strategies in endocrine resistant ER-positive breast cancer, with varying levels of evidence for improvement in outcomes (Table 1).

Table 1. Mechanisms of Resistance to Endocrine Agents.

Mechanism of Resistance	Implications for Targeted Therapeutics
**Primary Resistance**	
Receptor Tyrosine Kinase/Growth Factor Signaling Pathway	
FGFR amplification	Unknown at this time[a]
EGFR/ERBB2 mutations	Unknown at this time
Cell Cycle Control Signaling Pathway	
Cyclin D1 amplification or expression	FDA approved[b]
MYC amplification and overexpression	Unknown at this time
Hormone Signaling Pathway	
Loss of ERα	Unknown at this time
Post-translational modification of ERα	Unknown at this time

Expression of ER coactivation/corepression factors	Unknown at this time
ESR1 mutations	Unknown at this time[c]
**Acquired Resistance**	
PI3K/AKT1/MTOR Signaling Pathway	
PI3K/AKT/mTOR pathway activation	FDA approved[d]
Mitogen-Activated Protein (MAP) Kinase Pathway	
MAPK/ERK pathway activation	Unknown at this time
Hormone Signaling Pathway	
ESR1 mutations	Unknown at this time[e]
Other	
Changes in the tumor microenvironment	Unknown at this time

[a] Turner et al. (2010) showed in preclinical studies that FGFR amplification promoted resistance to endocrine therapies. FGFR-inhibitors dovitinib (NCT01528345) and nintedanib (NCT01658462) phase 2 trials are ongoing in patients with ER-positive and/or HER2-negative breast cancer.

[b] Alterations that cause the secondary signals in the ESR1 and CDK4/6 pathway to become activated include cyclin D1 amplification and CDK4/6 gain. Cyclin D1 is a transcriptional target of the estrogen receptor and is involved in regulating entry into the synthesis phase (S phase) of the cell cycle. Cyclin D1 binds to cyclin dependent kinases 4 and 6 (CDK4/6), and this complex phosphorylates the retinoblastoma (RB1) tumor suppressor protein. The RB1 protein releases the transcription factors required for S phase entry in the cell cycle. Frequencies of genetic alterations have been described in cyclin D1 (14%), CDK4/6 (3%), and RB1 (5%) in primary untreated ER-positive breast cancer samples (Cerami et al. 2012; Gao et al. 2013). Palbociclib is a kinase inhibitor targeting cyclin-dependent kinase 4 (CDK4) and 6 (CDK6) granted accelerated approval in combination with letrozole for the treatment of postmenopausal women with HER2-negative hormone receptor positive breast cancer for first-line or initial endocrine therapy. Accelerated approval of the drug combination was based on the results of the randomized phase 2 trial PALOMA-I. In this study, 84 patients received combination therapy. The study showed an improvement in progression-free survival by 10 months (FDA 2015; Finn et al. 2015).

[c] ESR1 mutations are less common in primary breast cancers at the time of diagnosis (TCGA 2012), but they have been identified in up to 55% of ER-positive metastatic breast cancers that have been previously treated with antiestrogens in retrospective data sets. In vitro laboratory studies suggest that breast tumor cells harboring ESR1 mutations may not be as sensitive to antiestrogen therapy as wild type ER-positive breast tumor cells (Jeselsohn et al. 2014; Merenbakh-Lamin et al. 2013; Robinson et al. 2013; Toy et al. 2013).

[d] Hyper-activation of PI3K signaling occurs in 28-47% of ER-positive breast cancer samples, and leads to estrogen-dependent or estrogen-independent ER activity (Miller et al. 2010, 2011). Dual inhibition of the ER signaling pathway and the PIK3CA/mTOR pathway is a parallel targeting strategy that has been supported clinically. The mTOR inhibitor everolimus combined with endocrine therapy exemestane is an FDA-approved strategy for ER-positive metastatic breast cancer resistant to letrozole or anastrozole. Approval of the drug combination was based on the results of the randomized phase 3 trial BOLERO-2. In this study, 485 patients received combination therapy. The study showed an improvement in progression-free survival by 4.6 months; however, there was no change in overall survival (Baselga et al. 2012; FDA 2012; Piccart et al. 2014; Yardley et al. 2013).

[e] Several different ESR1 mutations in the ligand-binding domain of ESR1 have been identified in tumor samples from patients with ER-positive metastatic breast cancer after treatment with antiestrogen therapy, but these are rare in primary untreated tumors (Robinson et al. 2013; Toy et al. 2013). In functional modeling studies, these mutations confer constitutive ligand-independent activation of ER transcription and ERα expression and may mediate antiestrogen resistance (Jeselsohn et al. 2014; Merenbakh-Lamin et al. 2013; Robinson et al. 2013; Toy et al. 2013).

Contributors: Justin M. Balko, Pharm. D., Ph.D., Ingrid A. Mayer, M.D., M.S.C.I., Mia Levy, M.D., Ph.D., Carlos L. Arteaga, M.D.

Suggested Citation: Balko, J., I. Mayer, M. Levy, C. Arteaga. 2015. ER (ESR1) in Breast Cancer. *My Cancer Genome* https://www.mycancergenome.org/content/disease/breast-cancer/esr1/ (Updated July 6).

Last Updated: July 6, 2015

Disclaimer: The information presented at MyCancerGenome.org is compiled from sources believed to be reliable. Extensive efforts have been made to make this information as accurate and as up-to-date as possible. However, the accuracy and completeness of this information cannot be guaranteed. Despite our best efforts, this information may

contain typographical errors and omissions. The contents are to be used only as a guide, and health care providers should employ sound clinical judgment in interpreting this information for individual patient care.

MCG Home | About Us | Acknowledgments | Give | Site Map | Legal | APIs and Licensing

My Cancer Genome is managed by the Vanderbilt-Ingram Cancer Center Copyright © 2010 - 2016 MY CANCER GENOME

MY CANCER GENOME®
GENETICALLY INFORMED CANCER MEDICINE

Search My Cancer Genome Go

Home | DIRECT | About Us

- Breast Cancer
- AKT1
- AR
- ERBB2
- ESR1
  - ER Expression
  - ESR1 Mutations Resistant to Antiestrogen Therapy
- FGFR1
- FGFR2
- PGR
- PIK3CA
- PTEN

- Other Diseases

- Molecular Medicine

- Take Our Survey
- Glossary
- News
- Our Team
- Acknowledgements

What is ESR1? | ESR1 in Breast Cancer | ER Expression | Clinical Trials

ER (ESR1) Expression in Breast Cancer

**Properties**	
Frequency of ER expression in breast cancer	73–75% (Nadji et al. 2005; Rhodes et al. 2000)
**Implications for Targeted Therapeutics**	
**Hormone Signaling Pathway**	
Androgen receptor antagonists	Unknown at this time
Selective androgen receptor modulators	Unknown at this time
Selective estrogen receptor modulators	Primary sensitivity[a]
Estrogen receptor agonists/antagonists	Primary sensitivity[b]
Aromatase inhibitors	Primary sensitivity[c]
**PI3K/AKT1/MTOR Signaling Pathway**	
mTOR inhibitors	Primary sensitivity[d]
PIK3CA inhibitors	Unknown at this time
TORC1/TORC2 inhibitors	Unknown at this time
**Cell Cycle Control Signaling Pathway**	
CDK4/6 inhibitors	Primary sensitivity[e]
**Chromatin Remodeling/DNA Methylation Pathway**	
HDAC Inhibitors	Unknown at this time[f]
**Receptor Tyrosine Kinase/Growth Factor Signaling Pathway**	
EGFR inhibitors	Unknown at this time
HER2 inhibitors	Unknown at this time
FGFR inhibitors	Unknown at this time
IGF1R inhibitors	Unknown at this time

Estrogen receptor (ER) protein expression occurs in 73–75% of breast cancers (Nadji et al. 2005; Rhodes et al. 2000). ER expression, in general, occurs with progesterone receptor (PR) expression in breast cancer and also occurs in half of HER2-positive breast cancers. ER expression is an important predictor of efficacy for three classes of endocrine agents (Allred et al. 2009; Davies et al. 2012; EBCTCG 2011). Aromatase inhibitors (e.g., exemstane, letrozole, and anastrozole) block aromatase, so estrogen is not produced. Selective estrogen receptor modulators (SERMs; e.g., tamoxifen) compete with estrogen for binding to the ER. Selective estrogen receptor degraders or downregulators (SERDs; e.g., fulvestrant) bind to the ER, blocking estrogen from binding, and sometimes downregulate ER.

Multi-year adjuvant endocrine therapy with tamoxifen or an aromatase inhibitor is currently the standard-of-care treatment for early stage ER-positive breast cancer (NCCN 2013). For patients with metastatic ER-positive breast cancer, standard-of-care endocrine therapies include selective estrogen receptor modulators and aromatase inhibitors (NCCN 2013).

[a] Tamoxifen is a SERM that competes with ligand for ER and binds to ER to repress instead of activate it. Tamoxifen is approved for the treatment of ER-positive breast cancer (FDA 1977).

[b] Fulvestrant is an estrogen receptor antagonist that inhibits ER transcriptional activity in the nucleus and destabilizes ER. Fulvestrant is approved for the treatment of hormone receptor positive metastatic breast cancer in

postmenopausal women with disease progression after antiestrogen therapy (FDA 2002).

[c] Aromatase inhibitors inhibit the enzyme aromatase from synthesizing estrogen from androgen. Anastrozole and letrozole are non-steroidal aromatase inhibitors approved for treatment of postmenopausal women with hormone receptor positive breast cancer. Exemestane is a steroidal aromatase inhibitor approved treatment of postmenopausal women with ER-positive breast cancer (FDA 1995, 1997, 2005).

[d] Everolimus is a kinase inhibitor targeting mTOR that is FDA-approved as a combination therapy with exemestane for the treatment of postmenopausal women with HER2-negative hormone receptor positive breast cancer after failure of treatment with letrozole or anastrozole. Approval of the drug combination was based on the results of the randomized phase 3 trial BOLERO-2. In this study, 485 patients received combination therapy. The study showed an improvement in progression-free survival by 4.6 months; however, there was no change in overall survival (Baselga et al. 2012; FDA 2012; Piccart et al. 2014; Yardley et al. 2013a).

[e] Palbociclib is a kinase inhibitor targeting cyclin-dependent kinase 4 (CDK4) and 6 (CDK6) granted accelerated approval in combination with letrozole for the treatment of postmenopausal women with HER2-negative hormone receptor positive breast cancer for first-line or initial endocrine therapy. Accelerated approval of the drug combination was based on the results of the randomized phase 2 trial PALOMA-I. In this study, 84 patients received combination therapy. The study showed an improvement in progression-free survival by 10 months (FDA 2015; Finn et al. 2015).

[f] Entinostat is a selective histone deacetylase inhibitor that demonstrated a progression-free survival benefit in combination with exemestane in a randomized phase 2 trial of ER-positive breast cancer patients. Based on this phase 2 trial, the FDA designated entinostat as a breakthrough therapy for the treatment of ER-positive metastatic breast cancer (Yardley et al. 2013b).

Testing for ER Expression in Breast Cancer

Because ER and PR expression is predictive for response to endocrine therapy and prognostic for survival outcomes, accurate immunohistochemistry (IHC) measurements for ER and PR expression in breast cancer are important (Hammond et al. 2010).

Several different methods have been used to measure ER status in breast tumors. Per National Comprehensive Cancer Network (NCCN) guidelines, ER expression in invasive breast cancer or ductal carcinoma in situ (DCIS) tissues should be measured with validated IHC assays (Allred et al. 2009). The ASCO/CAP guideline recommendations for ER and PR testing by IHC in breast cancer patients specify the following algorithm for optimal ER/PR testing (Hammond et al. 2010):

1. Positive for ER or PR if finding of ≥ 1% of tumor cell nuclei are immunoreactive.
2. Negative for ER or PR if finding of < 1% of tumor cell nuclei are immunoreactive in the presence of evidence that the sample can express ER or PR (positive intrinsic controls are seen).
3. Uninterpretable for ER or PR if finding that no tumor nuclei are immunoreactive and that internal epithelial elements present in the sample or separately submitted from the same sample lack any nuclear staining.

Contributors: Justin M. Balko, Pharm. D., Ph.D., Ingrid A. Mayer, M.D., M.S.C.I., Mia Levy, M.D., Ph.D., Carlos L. Arteaga, M.D.

Suggested Citation: Balko, J., I. Mayer, M. Levy, C. Arteaga. 2015. ER (ESR1) Expression in Breast Cancer. *My Cancer Genome* https://www.mycancergenome.org/content/disease/breast-cancer/esr1/248/ (Updated July 6).

Last Updated: July 6, 2015

Disclaimer: The information presented at MyCancerGenome.org is compiled from sources believed to be reliable. Extensive efforts have been made to make this information as accurate and as up-to-date as possible. However, the accuracy and completeness of this information cannot be guaranteed. Despite our best efforts, this information may contain typographical errors and omissions. The contents are to be used only as a guide, and health care providers should employ sound clinical judgment in interpreting this information for individual patient care.

My Cancer Genome is managed by the Vanderbilt-Ingram Cancer Center Copyright © 2010 - 2016 MY CANCER GENOME

MY CANCER GENOME®
GENETICALLY INFORMED CANCER MEDICINE

Search My Cancer Genome Go

Home | DIRECT | About Us

- Acute Lymphoblastic Leukemia
- Acute Myeloid Leukemia
- Anaplastic Large Cell Lymphoma
- Basal Cell Carcinoma
- Bladder Cancer
- Breast Cancer
- Chronic Lymphocytic Leukemia
- Chronic Myeloid Leukemia
- Colorectal Cancer
- GIST
- Gastric Cancer
- Glioma
- Inflammatory Myofibroblastic Tumor
- Lung Cancer
- Medulloblastoma
- Melanoma
- Myelodysplastic Syndromes
- Neuroblastoma
- Ovarian Cancer
- Prostate Cancer
- Rhabdomyosarcoma
- Thymic Carcinoma
- Thyroid Cancer

- Molecular Medicine

- Take Our Survey
- Glossary
- News
- Our Team
- Acknowledgements

CDK4/CDK6 Inhibition and CDK4/CDK6 Inhibitors in Breast Cancer

Inhibition of CDK4/6 is a therapeutic strategy being investigated in patients with hormone receptor positive breast cancer in the adjuvant and neoadjuvant settings, as first-line therapy, and after resistance develops to endocrine therapy.

Cyclin D1 is a transcriptional target of the estrogen receptor and is involved in regulating entry into the synthesis phase (S phase) of the cell cycle. Cyclin D1 binds to cyclin dependent kinases 4 and 6 (CDK4/6), and this complex phosphorylates the retinoblastoma (RB1) tumor suppressor protein. The RB1 protein releases the transcription factors required for S phase entry in the cell cycle. CDK4/6 inhibitors block CDK4/6 from activating RB1 to promote the cell growth cycle.

Combining ER-targeting agents such as letrozole with agents that target downstream components of the ER pathway including CDK4/6 inhibitors is a vertical targeting strategy, and improves benefit cooperatively over standard of care single-agent therapy alone in clinical studies (Finn et al. 2015; Patnaik et al. 2014a, 2014b).

Several cell cycle inhibitors have been developed which target CDK4/6. Palbociclib is a kinase inhibitor targeting CDK4/6 that is FDA approved as a combination therapy with letrozole for the treatment of postmenopausal women with HER2-negative, hormone receptor positive breast cancer (FDA 2015; Finn et al. 2015). Ribociclib (LEE011) and abemaciclib (LY2835219) are two other kinase inhibitors targeting CDK4/6.

CDK4/6 Inhibitors in Breast Cancer

- palbociclib (Ibrance)
- ribociclib
- abemaciclib

Contributors: Justin M. Balko, Pharm. D., Ph.D., Ingrid A. Mayer, M.D., M.S.C.I., Mia Levy, M.D., Ph.D., Carlos L. Arteaga, M.D.

Suggested Citation: Balko, J., I. Mayer, M. Levy, C. Arteaga. 2015. CDK4/6 Inhibition and CDK4/6 Inhibitors in Breast Cancer. *My Cancer Genome* http://www.mycancergenome.org/content/molecular-medicine/cdk4cdk6-inhibition-and-cdk4cdk6-inhibitors-in-breast-cancer/ (Updated

Last Updated: June 24, 2015

Disclaimer: The information presented at MyCancerGenome.org is compiled from sources believed to be reliable. Extensive efforts have been made to make this information as accurate and as up-to-date as possible. However, the accuracy and completeness of this information cannot be guaranteed. Despite our best efforts, this information may contain typographical errors and omissions. The contents are to be used only as a guide, and health care providers should employ sound clinical judgment in interpreting this information for individual patient care.

MCG Home | About Us | Acknowledgments | Give | Site Map | Legal | APIs and Licensing

My Cancer Genome is managed by the Vanderbilt-Ingram Cancer Center Copyright © 2010 - 2016 MY CANCER GENOME

- Acute Lymphoblastic Leukemia
- Acute Myeloid Leukemia
- Anaplastic Large Cell Lymphoma
- Basal Cell Carcinoma
- Bladder Cancer
- Breast Cancer
- Chronic Lymphocytic Leukemia
- Chronic Myeloid Leukemia
- Colorectal Cancer
- GIST
- Gastric Cancer
- Glioma
- Inflammatory Myofibroblastic Tumor
- Lung Cancer
- Medulloblastoma
- Melanoma
- Myelodysplastic Syndromes
- Neuroblastoma
- Ovarian Cancer
- Prostate Cancer
- Rhabdomyosarcoma
- Thymic Carcinoma
- Thyroid Cancer

- Molecular Medicine

- Take Our Survey
- Glossary
- News
- Our Team
- Acknowledgements

Palbociclib in Breast Cancer

Target	Development Name	Generic Name	Trade Name	Status
CDK4/6	PD0332991	palbociclib	Ibrance	FDA approved in combination with letrozole for ER-positive breast cancer[a]

[a] ER-positive, HER2-negative first-line metastatic breast cancer patients treated with palbociclib in combination with the aromatase inhibitor letrozole demonstrated an improvement in median progression-free survival compared with letrozole alone in a randomized phase 2 trial (Table 1; PALOMA-1; Finn et al. 2015). Based on the outcomes from this trial, the combination of palbociclib and letrozole received accelerated FDA approval for ER-positive, HER2-negative first-line metastatic breast cancer in 2015 (FDA 2015). The phase 3 trial of this combination is still ongoing.

A phase III trial evaluating palbociclib in combination with fulvestrant in metastatic ER-positive, HER2-negative breast cancer following disease progression during or after endocrine therapy showed improved progression-free survival relative to patients treated with placebo plus fulvestrant (Table 1; PALOMA-3; Turner et al. 2015). The trial was stopped early due to efficacy. In a subgroup analysis, premenopausal/perimenopausal patients with ovarian suppression demonstrated equivalent efficacy benefit with combination therapy as postmenopausal breast cancer patients (Turner et al. 2015).

In a preliminary report of a phase 1 study of palbociclib in combination with paclitaxel in RB+ third-line metastatic breast cancer (any ER status), patients experienced a 73% clinical benefit rate treated with combination therapy (Table 1; Clark et al. 2014).

RB1-positive metastatic breast cancer patients treated with single-agent palbociclib demonstrated a 19% clinical benefit rate and a median progression-free survival of 3.7 months. In a hormone receptor positive patient subset with greater than or equal to two prior lines of hormone therapy, the clinical benefit rate was 29% and the median progression-free survival was 5.0 months (Table 1; DeMichele et al. 2014).

Table 1. Reported Trials with Palbociclib in Breast Cancer.

Reference	Study Type / Phase	Therapeutic setting	Treatment Agent	Mutation Status / Group	# Pts in Study	RR	PFS (months)	OS (months)
Turner et al. 2015 (PALOMA-3)	Phase 3	≥1st (no limit to prior therapies) metastatic or advanced breast cancer	palbociclib + fulvestrant	HR+	347	10.4%	9.2	
			placebo + fulvestrant		174	6.3%	3.8	
Finn et al. 2015 (PALOMA-1; TRIO-18)	Phase 2	1st line metastatic breast cancer	letrozole	ER+ / HER2– (cohort 1+2)	81	27%	10.2	33.3
				ER+ / HER2– (cohort 1)	32		5.7	
				ER+ / HER2– / CCND1+ or p16+ (cohort 2)	49		11.1	
			letrozole + palbociclib	ER+ / HER2– (cohort 1+2)	84	43%	20.2	37.5
				ER+ / HER2– (cohort 1)	34		26.1	
				ER+ / HER2– /	50		18.1	

				CCND1+ or p16+ (cohort 2)				
DeMichele et al. 2014	Phase 2	≥1st (no limit to prior therapies) metastatic breast cancer	palbociclib	RB1+	37		3.7	
				HR+ / RB1+ subset	33		5.1	
		≥2 prior lines of hormone therapy		HR+ / RB1+ subset	24		5.0	
Clark et al. 2014	Phase 1	≥3rd line metastatic breast cancer	palbociclib + paclitaxel	RB1+	15	40%		

NOTE: CR = complete response; ER = estrogen receptor; OS = overall survival; PFS = progression-free survival; PR = partial response; Pts = patients; RR = response rate (CR + PR); RB1 = retinoblastoma.

Table 2. Ongoing and Recruiting Clinical Investigation with Palbociclib in Breast Cancer.

**Study Type / Phase / ID**	**Therapeutic setting**	**Prior therapy requirement**	**Treatment Agent**	**Mutation Status / Group**	**# Pts in Study**	**Study Start Date**
Phase 1b (NCT01976169)	2nd line or greater, recurrent or metastatic breast cancer	Prior trastuzumab or HER2 targeted therapies required	palbociclib + Trastuzumab-DM1	HER2+ RB1-proficient	17	January 2014
Phase 3 (PEARL, NCT02028507)	Any line, locally advanced or metastatic breast cancer	Recurrence during or within 12 months of adjuvant letrozole or anastrozole or during or within 1 month of letrozole or anastrozole for metastatic disease	palbociclib + exemestane	ER+ and/or PR+ HER2–	348	March 2014
			capecitabine			
Phase 3 (PALOMA-2, NCT01740427)	1st line	No prior systemic anti-cancer therapy for advanced ER+ disease	palbociclib + letrozole	ER+ HER2–	650	February 2013
			placebo + letrozole			
Phase 3 (PENELOPE-B, NCT01864746)	Early breast cancer at high risk of relapse after showing less than pathological complete response to neoadjuvant taxane-containing chemotherapy	Prior neoadjuvant chemotherapy including taxane of at least 16 weeks	palbociclib + standard anti-hormonal therapy	ER+ and/or PR+ HER2–	800	November 2013
			Placebo + standard anti-hormonal therapy			
Phase 2 (NCT02040857)	Adjuvant setting, breast cancer	One month of adjuvant tamoxifen or aromatase inhibitor; at least two more years of adjuvant therapy planned	palbociclib + tamoxifen or letrozole or anastrozole or exemestane	ER+ and/or PR+ HER2–	120	January 2014
Phase 2 (NCT01723774)	Neoadjuvant setting		palbociclib + anastrozole or anastrozole + goserelin	ER+ and/or PR+ HER2– PIK3CA mutation cohort	29	June 2013

NOTE: ER = estrogen receptor; HER2 = human epidermal growth factor receptor 2; PR = progesterone receptor; RB1 = retinoblastoma.

Contributors: Justin M. Balko, Pharm. D., Ph.D., Ingrid A. Mayer, M.D., M.S.C.I., Mia Levy, M.D., Ph.D., Carlos L. Arteaga, M.D.

Suggested Citation: Balko, J., I. Mayer, M. Levy, C. Arteaga. 2015. Palbociclib in Breast Cancer. *My Cancer Genome* http://www.mycancergenome.org/content/molecular-medicine/palbociclib-breast-cancer/ (Updated June 17).

Last Updated: July 24, 2015

Disclaimer: The information presented at MyCancerGenome.org is compiled from sources believed to be reliable. Extensive efforts have been made to make this information as accurate and as up-to-date as possible. However, the

accuracy and completeness of this information cannot be guaranteed. Despite our best efforts, this information may contain typographical errors and omissions. The contents are to be used only as a guide, and health care providers should employ sound clinical judgment in interpreting this information for individual patient care.

MCG Home | About Us | Acknowledgments | Give | Site Map | Legal | APIs and Licensing

My Cancer Genome is managed by the Vanderbilt-Ingram Cancer Center Copyright © 2010 - 2016 MY CANCER GENOME

MY CANCER GENOME®
GENETICALLY INFORMED CANCER MEDICINE

Search My Cancer Genome | Go

Home | DIRECT | About Us

- Acute Lymphoblastic Leukemia
- Acute Myeloid Leukemia
- Anaplastic Large Cell Lymphoma
- Basal Cell Carcinoma
- Bladder Cancer
- Breast Cancer
- Chronic Lymphocytic Leukemia
- Chronic Myeloid Leukemia
- Colorectal Cancer
- GIST
- Gastric Cancer
- Glioma
- Inflammatory Myofibroblastic Tumor
- Lung Cancer
- Medulloblastoma
- Melanoma
- Myelodysplastic Syndromes
- Neuroblastoma
- Ovarian Cancer
- Prostate Cancer
- Rhabdomyosarcoma
- Thymic Carcinoma
- Thyroid Cancer

- Molecular Medicine

- Take Our Survey
- Glossary
- News
- Our Team
- Acknowledgements

Ribociclib in Breast Cancer

Target	Development Name	Generic Name	Trade Name	Status
CDK4/6	LEE011	ribociclib		Trials completed with results[a]

[a] In a phase 1 study, an ER-positive breast cancer patient demonstrated a partial response when treated with single-agent ribociclib, one of two partial responses in a 70-patient cohort. The most common toxicities reported were neutropenia, leukopenia, nausea, and fatigue (Infante et al. 2014). In preliminary results from a phase 1b study, six patients with ER-positive, HER2-negative locally advanced or metastatic breast cancer were treated with ribociclib in combination with letrozole with one dose-limiting toxicity (neutropenia) reported. A 67% clinical benefit rate was reported in six patients (Table 1; Munster et al. 2014a, 2014b).

Table 1. Reported Trials with Ribociclib in Breast Cancer.

Reference	Study Type / Phase	Therapeutic setting	Treatment Agent	Mutation Status / Group	# Pts in Study	RR	PFS (months)	OS (months)
Munster et al. 2014a, 2014b (NCT01872260)	Phase 1b	1st line metastatic breast cancer	ribociclib + letrozole	ER+ / HER2–	6	67%		

NOTE: CR = complete response; ER = estrogen receptor; OS = overall survival; PFS = progression-free survival; PR = partial response; Pts = patients; RR = response rate (CR + PR).

Table 2. Ongoing Clinical Investigation with Ribociclib in Breast Cancer.

Study Type / Phase / ID	Therapeutic setting	Prior therapy requirement	Treatment Agent	Mutation Status/Group	# Patients in Study	Study Start Date
Phase 2 (SIGNATURE, NCT02187783)	2nd line or greater, any cancer except non-triple negative breast cancer		ribociclib	CDK4/6, cyclin D1/3, or p16 aberrations	90	August 2014
Phase 1b/2 (NCT01857193)	1st or greater, locally advanced or metastatic breast cancer, postmenopausal	Recurrence during or within 12 months of adjuvant letrozole or anastrozole, or during or within 1 month of letrozole or anastrozole for metastatic disease	ribociclib + everolimus + exemestane	ER+ HER2–	185	September 2013
			ribociclib + exemestane			
			everolimus + exemestane			
Phase 1b/2 (NCT02088684)	Any line, locally advanced or metastatic breast cancer, postmenopausal		ribociclib + BKM120 + fulvestrant	ER+ HER2–	216	May 2014
			ribociclib + BYL719 + fulvestrant			
			ribociclib + fulvestrant			
Phase 1 (NCT02154776)	1st line, locally advanced or metastatic breast cancer; 2nd line or greater in the dose escalation phase		ribociclib + buparlisib + letrozole	ER+ HER2–	50	June 2014
Phase 1b/2 (NCT01872260)	1st line, locally advanced or	No progression within 12 months	ribociclib + letrozole	ER+ HER2–	300	October 2013

	metastatic breast cancer, postmenopausal (phase 2, phase 1b dose expansions); Any line (Phase 1b dose escalation)	of completing adjuvant letrozole	BYL719 + letrozole<br>ribociclib + BYL719 + letrozole			
Phase 2 (MONA LEESA-1, NCT01919229)	1st line, locally advanced or metastatic breast cancer, postmenopausal		ribociclib 400 mg + letrozole<br>ribociclib 600 mg + letrozole<br>letrozole	ER+ and/or PR+ HER2–	120	October 2013
Phase 3 (MONA LEESA-2, NCT01958021)	1st line, locally advanced or metastatic breast cancer, postmenopausal	No progression within 12 months of completing adjuvant letrozole or anastrole	ribociclib + letrozole<br>placebo + letrozole	ER+ and/or PR+ HER2–	500	December 2013
Phase 2 (SIGNATURE, NCT02187783)	2nd line or greater, any cancer, triple negative breast cancer		ribociclib	CDK4/6, cyclin D1/3, or p16 aberrations	90	August 2014

NOTE: ER = estrogen receptor; HER2 = human epidermal growth factor receptor 2; PR = progesterone receptor.

Contributors: Justin M. Balko, Pharm. D., Ph.D., Ingrid A. Mayer, M.D., M.S.C.I., Mia Levy, M.D., Ph.D., Carlos L. Arteaga, M.D.

Suggested Citation: Balko, J., I. Mayer, M. Levy, C. Arteaga. 2015. Ribociclib in Breast Cancer. *My Cancer Genome* http://www.mycancergenome.org/content/molecular-medicine/ribociclib-in-breast-cancer/ (Updated June 17).

Last Updated: July 22, 2015

Disclaimer: The information presented at MyCancerGenome.org is compiled from sources believed to be reliable. Extensive efforts have been made to make this information as accurate and as up-to-date as possible. However, the accuracy and completeness of this information cannot be guaranteed. Despite our best efforts, this information may contain typographical errors and omissions. The contents are to be used only as a guide, and health care providers should employ sound clinical judgment in interpreting this information for individual patient care.

MCG Home | About Us | Acknowledgments | Give | Site Map | Legal | APIs and Licensing

My Cancer Genome is managed by the Vanderbilt-Ingram Cancer Center Copyright © 2010 - 2016 MY CANCER GENOME

Abemaciclib in Breast Cancer

Target	Development Name	Generic Name	Trade Name	Status
CDK4/6	LY2835219	abemaciclib		Trials completed with results[a]

[a] In a phase 1 trial with several expansion cohorts, abemaciclib showed activity in several tumor types, including lung cancer and breast cancer, in preliminary reports. In an ER-positive metastatic breast cancer cohort, 36 patients treated with the combination of abemaciclib and fulvestrant demonstrated a clinical benefit rate of 81% (Table 1; Goldman et al. 2014; Patnaik et al. 2014a, 2014b; Shapiro et al. 2013).

Table 1. Reported Trial with Abemaciclib in Breast Cancer.

Reference	Study Type / Phase	Therapeutic setting	Treatment Agent	Mutation Status / Group	# Pts in Study	RR	PFS (months)	OS (months)
Patnaik et al. 2014a, 2014b	Phase 1	Metastatic breast cancer	abemaciclib	HR+	36	25%		
			abemaciclib + fulvestrant (expanded cohort)	HR+	13	62%		

NOTE: CR = complete response; ER = estrogen receptor; HR = hormone receptor (ER and/or PR); OS = overall survival; PFS = progression-free survival; PR = partial response; PR = progesterone receptor; Pts = patients; RR = response rate (CR + PR).

Table 2. Ongoing and Recruiting Clinical Investigation with Abemaciclib in Breast Cancer.

Study Type / Phase / ID	Therapeutic setting	Prior therapy requirement	Treatment Agent	Mutation Status/Group	# Patients in Study	Study Start Date
Phase 1 (NCT02057133)	1st line, locally advanced or metastatic breast cancer, pre- or postmenopausal	No prior prior systemic endocrine therapy	abemaciclib + letrozole	ER+ and/or PR+ HER2–	81	March 2014
	1st line, locally advanced or metastatic breast cancer, pre- or postmenopausal	No prior prior systemic endocrine therapy	abemaciclib + anastrozole			
	1st line or greater, locally advanced or metastatic breast cancer, pre- or postmenopausal		abemaciclib + tamoxifen			
	2nd line or greater, locally advanced or metastatic breast cancer, pre- or postmenopausal	Prior therapy with anastrozole or letrozole required	abemaciclib+ exemestane			
	2nd line or greater, locally advanced or metastatic breast cancer,	Prior therapy with anastrozole or letrozole required	abemaciclib + exemestane + everolimus			

	pre- or postmenopausal					
Phase 2 (MONARCH 1, NCT02102490)	1st line or greater, locally advanced or metastatic breast cancer	Disease progression after anti-estrogen therapy and 2 prior chemotherapy regimens required	abemaciclib	ER+ and/or PR+ HER2–	128	June 2014
Phase 3 (MONARCH 2, NCT02107703)	1st or 2nd line, locally advanced or metastatic breast cancer, postmenopausal	Either no prior endocrine therapy or progression during or within 12 months of adjuvant endocrine therapy or greater than 12 months and progressed after first-line endocrine therapy for metastatic disease, or progression after first-line therapy (no adjuvant therapy)	abemaciclib + fulvestrant	ER+ and/or PR+ HER2–	550	July 2014
			placebo + fulvestrant			
Phase 3 (MONARCH 3, NCT02246621)	1st line or greater, locally advanced or metastatic breast cancer, postmenopausal		abemaciclib + anastrozole or letrozole	ER+ and/or PR+ HER2–	450	October 2014, not yet recruiting
			anastrozole or letrozole + placebo			

NOTE: ER = estrogen receptor; HER2 = human epidermal growth factor receptor 2; PR = progesterone receptor.

Contributors: Justin M. Balko, Pharm. D., Ph.D., Ingrid A. Mayer, M.D., M.S.C.I., Mia Levy, M.D., Ph.D., Carlos L. Arteaga, M.D.

Suggested Citation: Balko, J., I. Mayer, M. Levy, C. Arteaga. 2015. Abemaciclib in Breast Cancer. *My Cancer Genome* http://www.mycancergenome.org/content/molecular-medicine/abemaciclib-in-breast-cancer/ (Updated June 17).

Last Updated: June 22, 2015

Disclaimer: The information presented at MyCancerGenome.org is compiled from sources believed to be reliable. Extensive efforts have been made to make this information as accurate and as up-to-date as possible. However, the accuracy and completeness of this information cannot be guaranteed. Despite our best efforts, this information may contain typographical errors and omissions. The contents are to be used only as a guide, and health care providers should employ sound clinical judgment in interpreting this information for individual patient care.

MCG Home | About Us | Acknowledgments | Give | Site Map | Legal | APIs and Licensing

My Cancer Genome is managed by the Vanderbilt-Ingram Cancer Center Copyright © 2010 - 2016 MY CANCER GENOME

MY CANCER GENOME®
GENETICALLY INFORMED CANCER MEDICINE

Search My Cancer Genome Go

Home DIRECT About Us

- Acute Lymphoblastic Leukemia
- Acute Myeloid Leukemia
- Anaplastic Large Cell Lymphoma
- Basal Cell Carcinoma
- Bladder Cancer
- Breast Cancer
- Chronic Lymphocytic Leukemia
- Chronic Myeloid Leukemia
- Colorectal Cancer
- GIST
- Gastric Cancer
- Glioma
- Inflammatory Myofibroblastic Tumor
- Lung Cancer
- Medulloblastoma
- Melanoma
- Myelodysplastic Syndromes
- Neuroblastoma
- Ovarian Cancer
- Prostate Cancer
- Rhabdomyosarcoma
- Thymic Carcinoma
- Thyroid Cancer

- Molecular Medicine

- Take Our Survey
- Glossary
- News
- Our Team
- Acknowledgements

MTOR Inhibition and MTOR Inhibitors in Breast Cancer

MTOR is a member of the phosphatidylinositol-3-kinase (PI3K)/AKT1/mechanistic target of rapamycin (MTOR) cell signaling pathway that affects cell growth and proliferation, protein translation and synthesis, and the regulation of apoptosis. The PI3K pathway may be activated by the binding of extracellular growth factors (e.g., IGF1, insulin-like growth factor 1) to their corresponding receptor tyrosine kinases or by activating mutations in PIK3CA, AKT1, TSC1, and others. The pathway is inhibited by phosphatase and tensin homolog (PTEN), which dephosphorylates phosphoinositide phosphates.

In patients whose tumors have demonstrated resistance to nonsteroidal aromatase inhibitors, parallel inhibition of the PI3K/AKT1/MTOR signaling pathway and the estrogen signaling pathway may provide additional improvement in efficacy.

Everolimus is a kinase inhibitor targeting MTOR approved in combination with exemestane for the treatment of postmenopausal women with HER2-negative hormone receptor positive breast cancer after failure of treatment with letrozole or anastrozole (FDA 2012). Temsirolimus in combination with letrozole has been evaluated in a randomized phase 3 trial (HORIZON) in aromatase inhibitor-naïve, hormone receptor positive breast cancer patients. The trial was stopped at the second interim analysis due to futility, and no improvement in progression-free survival was demonstrated in the temsirolimus and letrozole combination arm in comparison with letrozole plus placebo arm (Wolff et al. 2012).

MTOR Inhibitors in Breast Cancer

- everolimus
- temsirolimus

TORC1/2 Inhibitors in Breast Cancer

- MLN0128

Contributors: Justin M. Balko, Pharm. D., Ph.D., Ingrid A. Mayer, M.D., M.S.C.I., Mia Levy, M.D., Ph.D., Carlos L. Arteaga, M.D.

Suggested Citation: Balko, J., I. Mayer, M. Levy, C. Arteaga. 2015. MTOR Inhibition and MTOR Inhibitors in Breast Cancer. *My Cancer Genome* http://www.mycancergenome.org/content/molecular-medicine/mtor-inhibition-and-mtor-inhibitors-in-breast-cancer/ (Updated June

Last Updated: June 24, 2015

Disclaimer: The information presented at MyCancerGenome.org is compiled from sources believed to be reliable. Extensive efforts have been made to make this information as accurate and as up-to-date as possible. However, the accuracy and completeness of this information cannot be guaranteed. Despite our best efforts, this information may contain typographical errors and omissions. The contents are to be used only as a guide, and health care providers should employ sound clinical judgment in interpreting this information for individual patient care.

MCG Home | About Us | Acknowledgments | Give | Site Map | Legal | APIs and Licensing
My Cancer Genome is managed by the Vanderbilt-Ingram Cancer Center Copyright © 2010 - 2016 MY CANCER GENOME

MY CANCER GENOME®
GENETICALLY INFORMED CANCER MEDICINE

Search My Cancer Genome Go

Home DIRECT About Us

- Acute Lymphoblastic Leukemia
- Acute Myeloid Leukemia
- Anaplastic Large Cell Lymphoma
- Basal Cell Carcinoma
- Bladder Cancer
- Breast Cancer
- Chronic Lymphocytic Leukemia
- Chronic Myeloid Leukemia
- Colorectal Cancer
- GIST
- Gastric Cancer
- Glioma
- Inflammatory Myofibroblastic Tumor
- Lung Cancer
- Medulloblastoma
- Melanoma
- Myelodysplastic Syndromes
- Neuroblastoma
- Ovarian Cancer
- Prostate Cancer
- Rhabdomyosarcoma
- Thymic Carcinoma
- Thyroid Cancer

- Molecular Medicine

- Take Our Survey
- Glossary
- News
- Our Team
- Acknowledgements

Everolimus in Breast Cancer

Target	Development Name	Generic Name	Trade Name	Status
MTOR	RAD001	everolimus	Afinitor	FDA approved in combination with exemestane for ER-positive breast cancer[a]

[a] Everolimus is a kinase inhibitor targeting MTOR that has been FDA approved as a combination therapy with exemestane for the treatment of postmenopausal women with HER2-negative, hormone receptor positive breast cancer after failure of treatment with letrozole or anastrozole (FDA 2012). The FDA approval of this drug combination was based on the results of the phase 3 trial BOLERO-2. In this study, 485 patients received combination therapy. The study showed an improvement in progression-free survival by 4.6 months; however, there was no change in overall survival (Baselga et al. 2012; Piccart et al. 2014; Yardley et al. 2013).

Table 1. Phase 3 Trials with Reported or Preliminary Results with Everolimus in Breast Cancer.

Reference	Study Type / Phase	Therapeutic Setting	Treatment Agent	Mutation Status / Group	# Pts in Study	RR	PFS (months)	OS (months)
Hurvitz et al. 2014 (BOLERO-1)	Phase 3	First-line metastatic breast cancer	everolimus + trastuzumab + paclitaxel	HER2+	480		15	
			placebo + trastuzumab + paclitaxel		239		14.5	
Baselga et al. 2012; Piccart et al. 2014; Yardley et al. 2013 (BOLERO-2)	Phase 3	Second-line metastatic breast cancer	everolimus + exemestane	HR+ / HER2–	485	13%	7.8	31
			placebo + exemestane		239	2%	3.2	26.6
André et al. 2014 (BOLERO-3)	Phase 3	First-line or greater metastatic breast cancer	everolimus + trastuzumab + vinorelbine	HER2+	284	41%	7	
			placebo + trastuzumab + vinorelbine		285	37%	5.8	
von Minckwitz et al. 2014 (GeparQuinto)	Phase 3	Neoadjuvant	epirubicin + cyclophosphamide; followed by docetaxel	HER2–	969			88.7% (estimated 3-year survival)
			Epirubicin + cyclophosphamide + bevacizumab; followed by docetaxel + bevacizumab		969			90.7% (estimated 3-year survival)
			epirubicin + cyclophosphamide +/- bevacizumab (with no response); followed by paclitaxel		198			83.4% (estimated 3-year survival)
			epirubicin + cyclophosphamide +/- bevacizumab (with no response); followed by		197			81.4% (estimated 3-year survival)

			paclitaxel + everolimus					

NOTE: CR = complete response; ER = estrogen receptor; HR = hormome receptor (ER and/or PR); OS = overall survival; PFS = progression-free survival; PR = partial response; PR = progesterone receptor; Pts = patients; RR = response rate (CR + PR).

Table 2. Ongoing and Recruiting Clinical Investigation with Everolimus in Breast Cancer.

Study Type / Phase / ID	Therapeutic Setting	Prior Therapy Requirement	Treatment Agent	Mutation Status/Group	# Patients in Study	Study Start Date
Phase 3 (FEVEX, NCT02404051)	2nd line	Refractory to NSAI	fulvestrant followed by everolimus + exemestane	HR+ HER2–	745	May 2015
			exemestane + everolimus followed by fulvestrant	HR+ HER2–		
Phase 3 (NCT01674140)	Adjuvant	completed standard neoadjuvant or adjuvant chemotherapy	endocrine therapy + everolimus	HR+ HER2–	3500	April 2013
			endocrine therapy + placebo			
Phase 3 (NCT01805271)	Adjuvant	At least 1 year but not more than 4 years of adjuvant hormone therapy	Adjuvant hormone therapy + everolimus	HR+ HER2–	1984	March 2013
			adjuvant hormone therapay + everolimus			

NOTE: ER = estrogen receptor; HR = hormome receptor (ER and/or PR); NSAI = nonsteroidal aromatase inhibitor; PR = progesterone receptor.

Inhibition of CDK4/6 is a therapeutic strategy being investigated in patients with hormone receptor positive breast cancer in the adjuvant and neoadjuvant settings, as first-line therapy, and after resistance develops to endocrine therapy.

Cyclin D1 is a transcriptional target of the estrogen receptor and is involved in regulating entry into the synthesis phase (S phase) of the cell cycle. Cyclin D1 binds to cyclin dependent kinases 4 and 6 (CDK4/6), and this complex phosphorylates the retinoblastoma (RB1) tumor suppressor protein. The RB1 protein releases the transcription factors required for S phase entry in the cell cycle. CDK4/6 inhibitors block CDK4/6 from activating RB1 to promote the cell growth cycle.

Combining ER-targeting agents such as letrozole with agents that target downstream components of the ER pathway including CDK4/6 inhibitors is a vertical targeting strategy, and improves benefit cooperatively over standard of care single-agent therapy alone in clinical studies (Finn et al. 2015; Patnaik et al. 2014a, 2014b).

Several cell cycle inhibitors have been developed which target CDK4/6. Palbociclib is a kinase inhibitor targeting CDK4/6 that is FDA approved as a combination therapy with letrozole for the treatment of postmenopausal women with HER2-negative, hormone receptor positive breast cancer (FDA 2015; Finn et al. 2015). Ribociclib (LEE011) and abemaciclib (LY2835219) are two other kinase inhibitors targeting CDK4/6.

Contributors: Justin M. Balko, Pharm. D., Ph.D., Ingrid A. Mayer, M.D., M.S.C.I., Mia Levy, M.D., Ph.D., Carlos L. Arteaga, M.D.

Suggested Citation: Balko, J., I. Mayer, M. Levy, C. Arteaga. 2015. Everolimus in Breast Cancer. *My Cancer Genome* http://www.mycancergenome.org/content/molecular-medicine/everolimus-breast-cancer/ (Updated June 17).

Last Updated: June 22, 2015

Disclaimer: The information presented at MyCancerGenome.org is compiled from sources believed to be reliable. Extensive efforts have been made to make this information as accurate and as up-to-date as possible. However, the accuracy and completeness of this information cannot be guaranteed. Despite our best efforts, this information may contain typographical errors and omissions. The contents are to be used only as a guide, and health care providers should employ sound clinical judgment in interpreting this information for individual patient care.

My Cancer Genome is managed by the Vanderbilt-Ingram Cancer Center Copyright © 2010 - 2016 MY CANCER GENOME
