# Supplementary material for: Internet-Based Assessment of Oncology Health Care Professional Learning Style and Optimization of Materials for Web-Based Learning: Controlled Trial With Concealed Allocation
Source: J Med Internet Res. 2017 Jul 25;19(7):e265. doi: 10.2196/jmir.7506 (PMC5548983; doi:10.2196/jmir.7506)
Supplement: Multimedia Appendix 7 [file jmir_v19i7e265_app7.pdf]

Multimedia Appendix 7

Feedback Survey Results

Table 1. Results of the question, "was the information easy to understand?" by arm and learning style.

Participant Group (N)	Was the Information Easy to Understand? n (%)				
	Strongly Disagree	Disagree	Neither Agree Nor Disagree	Agree	Strongly Agree
Control (292)	10 (3.4)	19 (6.5)	41 (14.0)	170 (58.2)	52 (17.8)
Intervention (253)	18 (7.1)	13 (5.1)	19 (7.5)	127 (50.2)	76 (30.0)
Watching (19)	0 (0)	4 (21)	3 (16)	8 (42)	4 (21)
Listening (12)	1 (8)	1 (8)	1 (8)	7 (58)	2 (17)
Reading (80)	2 (3)	6 (8)	8 (10)	41 (51)	23 (29)
Watching & Listening (53)	4 (8)	2 (4)	2 (4)	31 (59)	14 (26)
Watching & Reading (101)	3 (3.0)	5 (5.0)	6 (5.9)	65 (64.4)	22 (21.8)
Listening & Reading (67)	5 (8)	1 (2)	11 (16)	38 (57)	12 (18)
Watching & Listening & Reading (213)	13 (6.1)	13 (6.1)	29 (13.6)	107 (50.2)	51 (23.9)

Table 2. Results of the question, "was the information confusing?" by arm and learning style.

Participant Group (N)	Was the Information Confusing? n (%)				
	Strongly Disagree	Disagree	Neither Agree Nor Disagree	Agree	Strongly Agree
Control (291)	20 (6.9)	97 (33.3)	61 (21.0)	105 (36.1)	8 (2.8)
Intervention (252)	39 (15.5)	105 (41.7)	47 (18.6)	55 (21.8)	6 (2.4)
Watching (19)	3 (16)	7 (37)	4 (21)	5 (26)	0 (0)
Listening (12)	1 (8)	3 (25)	1 (8)	7 (58)	0 (0)
Reading (82)	12 (15)	30 (37)	17 (21)	22 (27)	1 (1)
Watching & Listening (52)	8 (15)	18 (35)	13 (25)	12 (23)	1 (2)
Watching & Reading (100)	8 (8.0)	38 (38.0)	20 (20.0)	30 (30.0)	4 (4.0)
Listening & Reading (66)	4 (6)	27 (41)	13 (20)	21 (32)	1 (2)

Watching & Listening & Reading (212)	23 (10.8)	79 (37.3)	40 (18.9)	63 (29.7)	7 (3.3)

Table 3. Results of the question, "was the information satisfying?" by arm and learning style.

Participant Group (N)	Was the Information Satisfying? n (%)				
	Strongly Disagree	Disagree	Neither Agree Nor Disagree	Agree	Strongly Agree
Control (294)	4 (1.4)	15 (5.1)	54 (18.4)	175 (59.5)	46 (15.6)
Intervention (252)	13 (5.2)	6 (2.4)	18 (7.1)	150 (59.5)	65 (25.8)
Watching (19)	0 (0)	2 (11)	3 (16)	10 (53)	4 (21)
Listening (12)	1 (8)	0 (0)	2 (17)	7 (58)	2 (17)
Reading (82)	1 (1)	3 (4)	10 (12)	50 (61)	18 (22)
Watching & Listening (53)	0 (0)	0 (0)	10 (19)	33 (62)	10 (19)
Watching & Reading (101)	2 (2.0)	7 (6.9)	9 (8.9)	61 (60.4)	22 (21.8)
Listening & Reading (67)	2 (3)	1 (2)	9 (13)	45 (67)	10 (15)
Watching & Listening & Reading (212)	11 (5.2)	8 (3.8)	29 (13.7)	119 (56.1)	42 (21.2)

Table 4. Results of the question, "was the information new?" by arm and learning style.

Participant Group (N)	Was the Information New? n (%)				
	Strongly Disagree	Disagree	Neither Agree Nor Disagree	Agree	Strongly Agree
Control (293)	5 (1.7)	1 (0.3)	25 (8.5)	173 (59.0)	89 (30.4)
Intervention (252)	10 (4.0)	4 (1.6)	11 (4.4)	127 (50.4)	100 (39.7)
Watching (19)	0 (0)	1 (5)	2 (11)	10 (53)	6 (32)
Listening (12)	1 (8)	0 (0)	2 (17)	7 (58)	2 (17)
Reading (82)	1 (1)	2 (2)	7 (9)	44 (54)	28 (34)
Watching & Listening (52)	0 (0)	0 (0)	2 (4)	34 (65)	16 (31)
Watching & Reading (101)	3 (3.0)	0 (0.0)	5 (5.0)	60 (59.4)	33 (32.7)
Listening & Reading (67)	2 (3)	0 (0)	2 (3)	39 (58)	24 (36)
Watching & Listening & Reading (212)	8 (3.8)	2 (0.9)	16 (7.6)	106 (50.0)	80 (37.7)

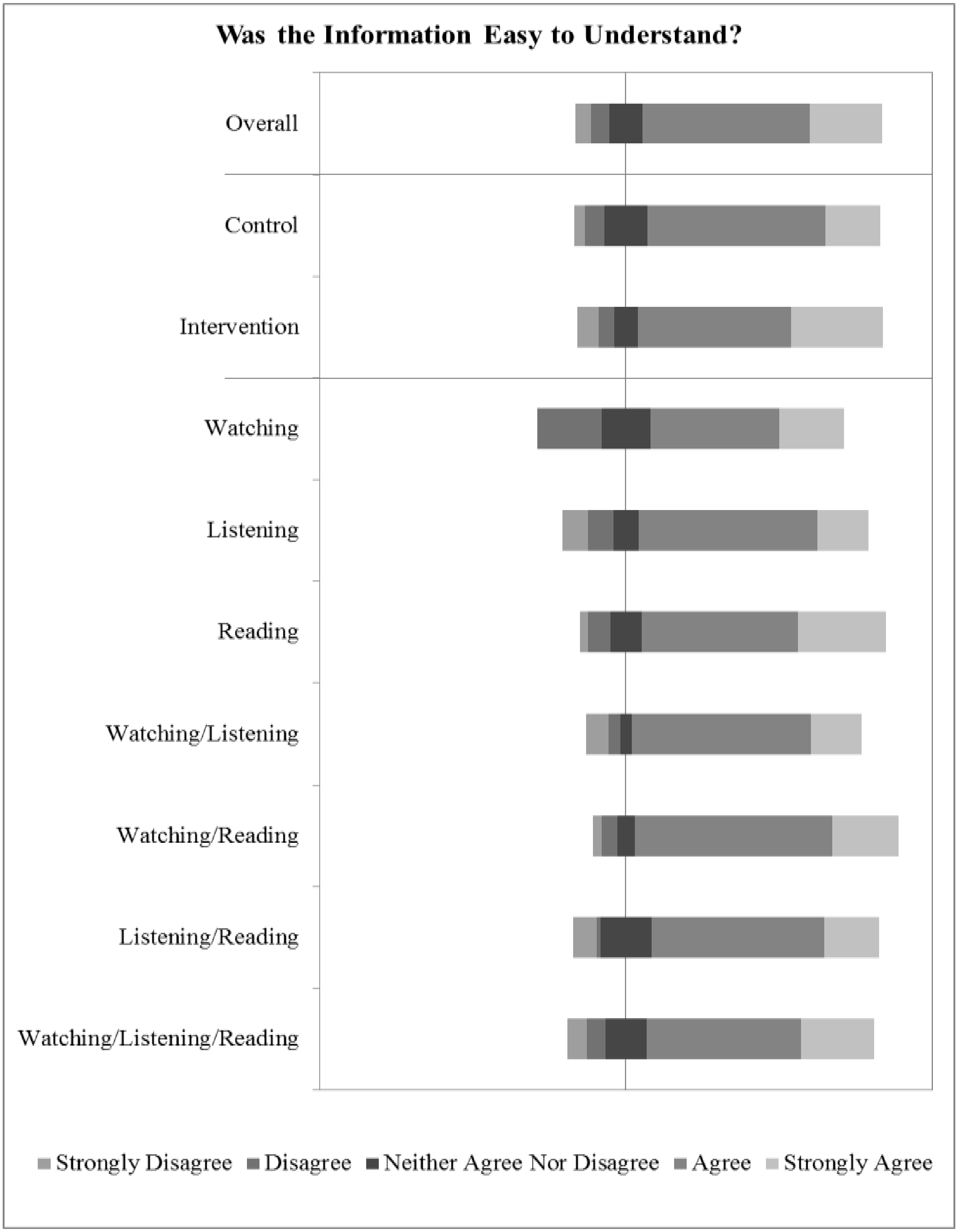

Figure 1. Feedback survey results on question "was the information easy to understand," by study arm and learning style.

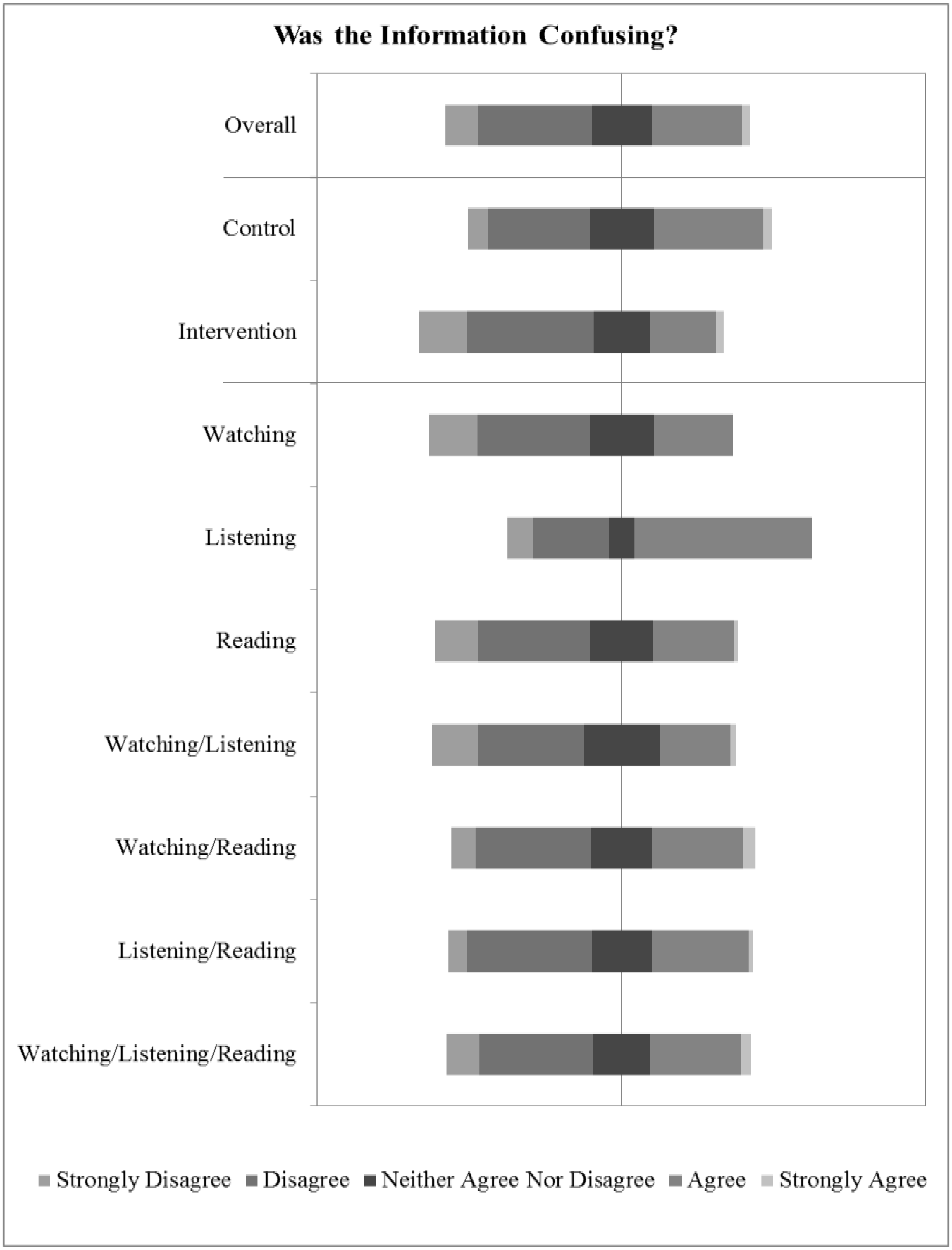

Figure 2. Feedback survey results on question "was the information confusing," by study arm and learning style.

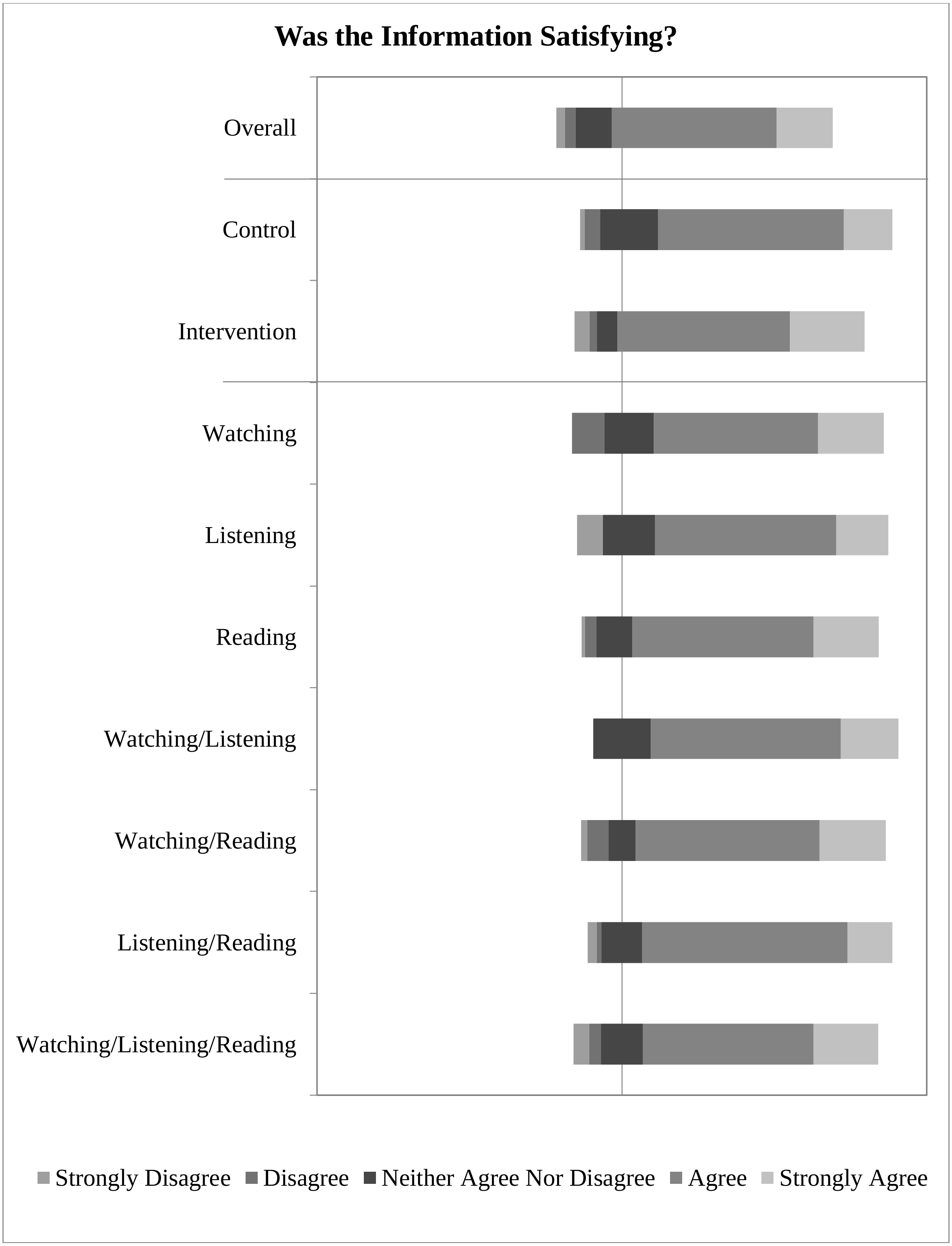

Figure 3. Feedback survey results on question "was the information satisfying," by study arm and learning style.

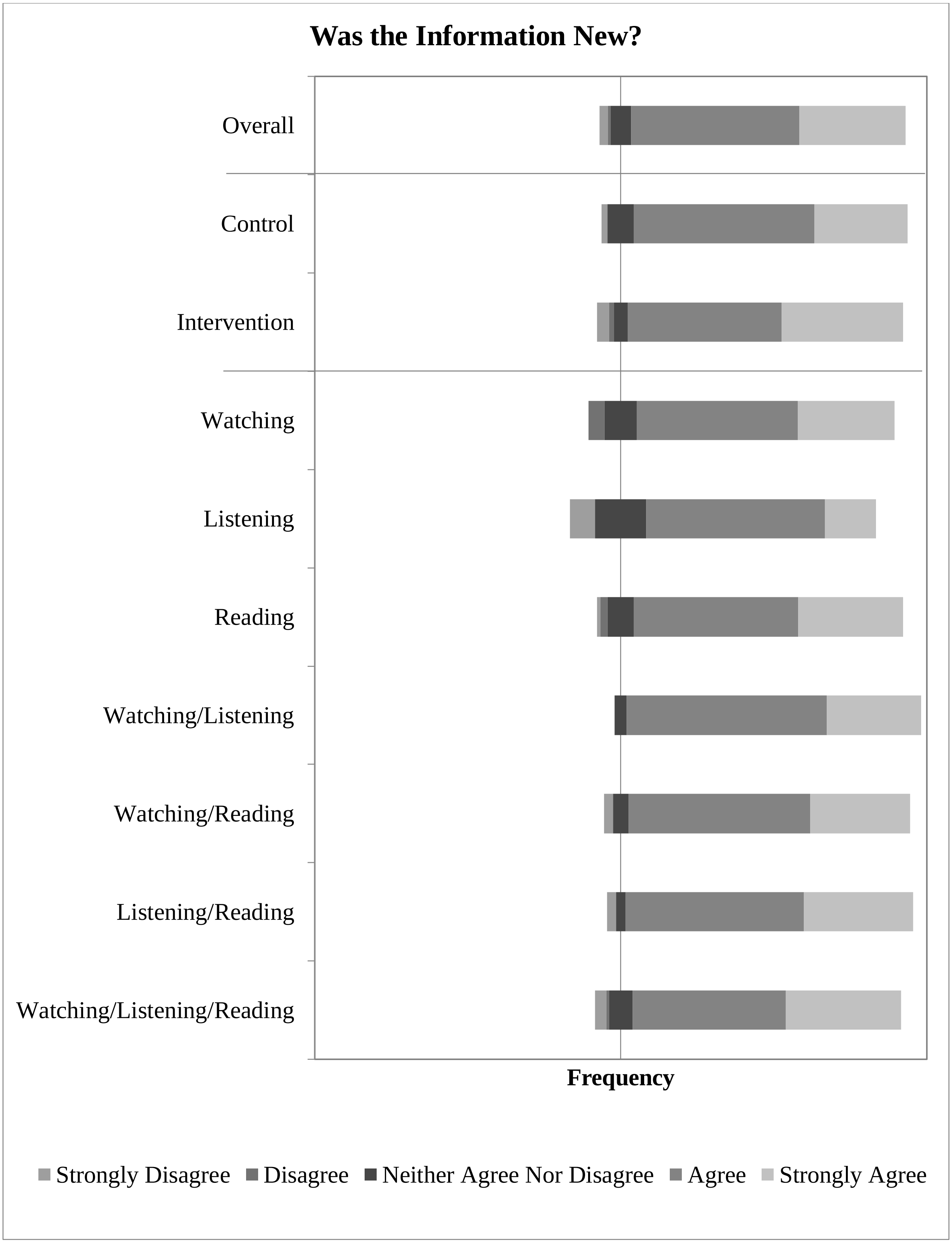

Figure 4. Feedback survey results on question "was the information new," by study arm and learning style.
