# Supplementary material for: Internet-Based Assessment of Oncology Health Care Professional Learning Style and Optimization of Materials for Web-Based Learning: Controlled Trial With Concealed Allocation
Source: J Med Internet Res. 2017 Jul 25;19(7):e265. doi: 10.2196/jmir.7506 (PMC5548983; doi:10.2196/jmir.7506)
Supplement: Multimedia Appendix 8 [file jmir_v19i7e265_app8.pdf]

Multimedia Appendix 8

Variance Analysis and Parameter Estimates

Table 1. Analysis of variance for knowledge posttest score regression model.

Variable	*df*	Partial SS	MS	F	*P*
Knowledge pretest score	1	293.033	293.033	114.58	<.0001
Group	1	21.365	21.365	8.35	0.0040
Practice type	2	10.137	5.069	1.98	0.1388
Percentage breast cancer patients seen	3	14.325	4.775	1.87	0.1341
Age	1	0.864	0.864	0.34	0.5613
Gender	1	2.387	2.387	0.93	0.3344
Race	2	1.745	0.872	0.34	0.7111
Physician specialty	6	27.199	4.533	1.77	0.1026
REGRESSION	17	347.448	20.438	7.99	<.0001
ERROR	534	1365.636	2.557		

NOTE: *df* = degrees of freedom; partial SS = variation, sum of squares; MS = mean of the squares (MS = SS/*df*); F = F test statistic (F = MS/MS_{ERROR}); *P* = *P*-value.

Table 2. Parameter estimates for knowledge posttest score regression model.

Variable	Coef	S.E.	t	*P*
Intercept	5.599	0.521	10.747	<0.001
Knowledge pretest score	0.358	0.033	10.704	<0.001
Group = intervention	0.401	0.139	2.890	0.004
Practice type = community	-0.176	0.169	-1.042	0.2980
Practice type = both/other	-0.500	0.255	-1.960	0.0500
Percentage breast cancer patients seen = 25-50 percent	-0.022	0.171	-0.129	0.8970
Percentage breast cancer patients seen = greater than 50 percent	-0.511	0.226	-2.264	0.0240
Percentage breast cancer patients seen = unknown	-0.284	0.520	-0.546	0.5850
Age	-0.005	0.008	-0.581	0.5610
Gender = female	0.152	0.158	0.966	0.3340
Race = Asian	-0.151	0.196	-0.770	0.4410
Race = other/multiracial	-0.123	0.308	-0.399	0.6900
Physician specialty = fellow	0.767	0.455	1.686	0.0920
Physician specialty = medical oncologist and/or hematologist	0.455	0.416	1.091	0.2750
Physician specialty = surgical oncologist	1.177	0.555	2.121	0.0340
Physician specialty = radiation oncologist	0.896	0.453	1.980	0.0480
Physician specialty = pathologist	0.630	0.508	1.239	0.2150
Physician specialty = None of the above	1.069	0.628	1.702	0.0890

NOTE: coef = regression coefficient; S.E. = standard error of the regression coefficient; t = t statistic; *P* = *P*-value.

Table 3. Analysis of variance for knowledge follow-up test score regression model.

Variable	*df*	Partial SS	MS	F	*P*
Knowledge pretest score	1	376.822	376.822	154.44	<.0001
Group	1	14.225	14.225	5.83	0.0161
Practice type	2	5.335	2.667	1.09	0.3359
Percentage breast cancer patients seen	3	0.970	0.323	0.13	0.9407
Age	1	0.121	0.121	0.05	0.8240
Gender	1	2.091	2.091	0.86	0.3550
Race	2	2.269	1.134	0.46	0.6284
Physician specialty	6	14.069	2.345	0.96	0.4510
REGRESSION	17	456.446	26.850	11.00	<.0001
ERROR	534	1302.936	2.440		

NOTE: *df* = degrees of freedom; partial SS = variation, sum of squares; MS = mean of the squares (MS = SS/*df*); F = F test statistic ($F = MS/MS_{ERROR}$); *P* = *P*-value.

Table 4. Parameter estimates for knowledge follow-up test score regression model.

Variable	Coef	S.E.	t	P
Intercept	4.376	0.505	8.668	<0.001
Knowledge pretest score	0.404	0.033	12.427	<0.001
Group = intervention	0.327	0.135	2.415	0.016
Practice type = community	-0.190	0.164	-1.154	0.2490
Practice type = both/other	-0.317	0.248	-1.279	0.2010
Percentage breast cancer patients seen = 25-50 percent	-0.103	0.170	-0.607	0.5440
Percentage breast cancer patients seen = greater than 50 percent	-0.072	0.224	-0.320	0.7490
Percentage breast cancer patients seen = unknown	0.005	0.508	0.009	0.9920
Age	0.002	0.008	0.222	0.8240
Gender = female	0.142	0.153	0.926	0.3550
Race = Asian	-0.124	0.181	-0.686	0.4930
Race = other/multiracial	-0.242	0.293	-0.825	0.4090
Physician specialty = fellow	0.908	0.459	1.981	0.0480
Physician specialty = medical oncologist and/or hematologist	0.588	0.411	1.431	0.1520
Physician specialty = surgical oncologist	0.556	0.563	0.988	0.3230
Physician specialty = radiation oncologist	0.536	0.436	1.230	0.2190
Physician specialty = pathologist	0.531	0.496	1.071	0.2840
Physician specialty = None of the above	1.179	0.607	1.944	0.0520

NOTE: coef = regression coefficient; S.E. = standard error of the regression coefficient; t = t statistic; *P* = *P*-value.
