# Supplementary material for: Internet-Based Assessment of Oncology Health Care Professional Learning Style and Optimization of Materials for Web-Based Learning: Controlled Trial With Concealed Allocation
Source: J Med Internet Res. 2017 Jul 25;19(7):e265. doi: 10.2196/jmir.7506 (PMC5548983; doi:10.2196/jmir.7506)
Supplement: Multimedia Appendix 3 [file jmir_v19i7e265_app3.pdf]

Multimedia Appendix 3

Table of Contents

Invitation email…… 2
Backup link to educational materials and invitation to complete knowledge posttest…… 4
Invitation to knowledge follow-up test…… 5
Confirmation of completion of the Amazon.com Gift Card survey…… 6
Email with the Amazon.com Gift Card…… 7

From: Mia Levy
To: Micheel, Christine M
Subject: My Cancer Genome survey - $100
Date: Monday, July 13, 2015 12:00:41 PM

This message contains graphics. If you do not see the graphics, click here to view.

MY CANCER GENOME®
GENETICALLY INFORMED CANCER MEDICINE

Dear Dr. Christine Micheel,

The MyCancerGenome.org team at Vanderbilt-Ingram Cancer Center is conducting a study to improve cancer genetics web pages for oncology physicians and nurses. My Cancer Genome invites you to participate in this study and receive a $100 Amazon.com Gift Card.* To participate, please click here.

For more information, click here.

Your PIN number is: fiHMzVjTOh

Thank you for your participation!

Mia Levy, MD, PhD

Co-Editor in Chief, My Cancer Genome
Assistant Professor of Biomedical Informatics and Medicine
Vanderbilt University Medical Center
2220 Pierce Avenue
691 Preston Research Building
Nashville, TN 37232-6838

**Amazon.com is not a sponsor of this offer. Except as required by law, Amazon.com Gift Cards ("GCs") cannot be transferred for value or redeemed for cash. GCs may be used only for purchases of eligible goods at Amazon.com or certain of its affiliated websites. For complete terms and conditions, see www.amazon.com/gc-legal. GCs are issued by ACI Gift Cards, Inc., a Washington corporation. All Amazon ®, ™ & © are IP of Amazon.com, Inc. or its affiliates. No*

expiration date or service fees.

This e-mail powered by Med-E-Mail connects healthcare professionals with relevant commercial messages germane to the practice of medicine.
If you wish to no longer receive messages from this sender please review your preference page here.

**From:**	mialevy.mycancergenome@vanderbilt.edu
**To:**	Micheel, Christine M
**Subject:**	Next Step - My Cancer Genome Survey - $100
**Date:**	Wednesday, April 13, 2016 7:51:19 PM

Thank you for participating in the My Cancer Genome study to improve cancer genetics web pages for oncology physicians and nurses.

We are sending the links below in case you closed the page with the link to your learning materials or to your "post-learning material review survey" in your browser. If you have already completed the "post-learning material review survey", please ignore this email.

Here is your link to the learning materials: Link to Learning Materials (http://www.mycancergenome.org/learning-material/?record=276&ls=I learn it by reading, listening, and watching&pin=jAhoij89A).

When you are finished reviewing the learning materials, please click on your personalized link below to continue the study by taking the "post-learning material review survey."

You may open the survey in your web browser by clicking the link below:
Post-Learning Material Review Survey

If the link above does not work, try copying the link below into your web browser:
https://redcap.vanderbilt.edu/surveys/?s=Rc3Dy4kkgU

This link is unique to you and should not be forwarded to others.

From: mialevy.mycancergenome@vanderbilt.edu
To: Micheel, Christine M
Subject: Final Step - My Cancer Genome survey - $100
Date: Wednesday, April 13, 2016 8:02:45 PM

Thank you for participating in the My Cancer Genome study to improve cancer genetics web pages for oncology physicians and nurses.

To continue, please click on your personalized survey link at the bottom of this email. After you answer these questions, you will receive your $100 Amazon.com Gift Card.* Please note that the survey will close if the study reaches its accrual limit.

Disclaimer:
*Amazon.com is not a sponsor of this offer. Except as required by law, Amazon.com Gift Cards ("GCs") cannot be transferred for value or redeemed for cash. GCs may be used only for purchases of eligible goods at Amazon.com or certain of its affiliated websites. For complete terms and conditions, see www.amazon.com/gc-legal. GCs are issued by ACI Gift Cards, Inc., a Washington corporation. All Amazon ®, ™ & © are IP of Amazon.com, Inc. or its affiliates. No expiration date or service fees.

SURVEY LINK:

You may open the survey in your web browser by clicking the link below:
Follow-up Survey

If the link above does not work, try copying the link below into your web browser:
https://redcap.vanderbilt.edu/surveys/?s=VzwFDCtYaa

This link is unique to you and should not be forwarded to others.

From: mialevy.mycancergenome@vanderbilt.edu
To: Micheel, Christine M
Subject: Gift Card Information - My Cancer Genome survey - $100
Date: Wednesday, April 13, 2016 8:51:21 PM

The MyCancerGenome.org team thanks you for your participation in the study. If you indicated that you want to receive the Amazon Gift Card,* we will now verify the information you provided and issue your Amazon.com Gift Card. Participants who provided verifiable information will receive their gift cards within 6-8 weeks.

If you would like a copy of the slides, please download them here (http://www.mycancergenome.org/learning-material/slides).

If you have questions or comments contact us via email at mialevy.mycancergenome@vanderbilt.edu or by phone by calling Christine Micheel, Ph.D. (study coordinator and My Cancer Genome managing editor), at 615-933-9368.

Regards,

Mia Levy, MD, PhD

Co-Editor in Chief, My Cancer Genome
Assistant Professor of Biomedical Informatics and Medicine
Vanderbilt University Medical Center
2220 Pierce Avenue
691 Preston Research Building
Nashville, TN 37232-6838

*Amazon.com is not a sponsor of this offer. Except as required by law, Amazon.com Gift Cards ("GCs") cannot be transferred for value or redeemed for cash. GCs may be used only for purchases of eligible goods at Amazon.com or certain of its affiliated websites. For complete terms and conditions, see www.amazon.com/gc-legal. GCs are issued by ACI Gift Cards, Inc., a Washington corporation. All Amazon ®, ™ & © are IP of Amazon.com, Inc. or its affiliates. No expiration date or service fees.

From: Mia Levy MyCancerGenome
To: Micheel, Christine M
Subject: Amazon.com Gift Card - My Cancer Genome survey - $100
Date: Thursday, January 19, 2017 2:19:21 PM
Attachments: image001.png
image002.png

MY CANCER GENOME®
GENETICALLY INFORMED CANCER MEDICINE

Thank you for participating in the My Cancer Genome study. It is our pleasure to send you this Amazon.com Gift Card* that can be redeemed towards millions of items at www.amazon.com. Don't delete this message—you'll need the gift card claim code below. You may want to print a copy of this message for easy reference later.

amazon.com gift card

Gift Card Amount:
$100.00

Claim Code:
@@@@-#@#@#@-@##@
Redeem Now

| **Apply to Account** | **How to Use** |

To redeem your gift card, follow these steps:

1. Visit www.amazon.com/gc.
2. Click Apply to Account and enter the claim code when prompted.
3. Gift card funds will be applied automatically to eligible orders during the checkout process.
4. You must pay for any remaining balance on your order with another payment method.

Your gift card claim code may also be entered when prompted during checkout. To redeem your gift card using the Amazon.com 1-Click® service, first add the gift card funds to Your Account.

If you have questions about redeeming your gift card, please visit www.amazon.com/gc-redeem. If you have questions regarding the My Cancer Genome study, please contact us via email at mialevy.mycancergenome@vanderbilt.edu or by phone by calling Christine Micheel, Ph.D. (study coordinator and My Cancer Genome managing editor), at 615-933-9368.

*Amazon.com is not a sponsor of this offer. Except as required by law, Amazon.com Gift Cards ("GCs") cannot be transferred for value or redeemed for cash. GCs may be used only for purchases of eligible goods on Amazon.com or certain of its affiliated websites. GCs cannot be redeemed for purchases of gift cards. Purchases are deducted from the GC balance. To redeem or view a GC balance, visit "Your Account" on Amazon.com. Amazon is not responsible if a GC is lost, stolen, destroyed or used without permission. For complete terms and conditions, see www.amazon.com/gc-legal. GCs are issued by ACI Gift Cards, Inc., a Washington corporation. All Amazon ™ & © are IP of Amazon.com, Inc. or its affiliates. No expiration date or service fees.
